# A comprehensive investigation of starch degradation process and identification of a transcriptional activator MabHLH6 during banana fruit ripening

**DOI:** 10.1111/pbi.12756

**Published:** 2017-06-30

**Authors:** Yun‐yi Xiao, Jian‐fei Kuang, Xin‐na Qi, Yu‐jie Ye, Zhen‐Xian Wu, Jian‐ye Chen, Wang‐jin Lu

**Affiliations:** ^1^ State Key Laboratory for Conservation and Utilization of Subtropical Agro‐bioresources/Guangdong Provincial Key Laboratory of Postharvest Science of Fruits and Vegetables College of Horticulture South China Agricultural University Guangzhou China

**Keywords:** banana fruit, starch degradation, iTRAQ, bHLH, transcriptional activator

## Abstract

Although starch degradation has been well studied in model systems such as *Arabidopsis* leaves and cereal seeds, this process in starchy fruits during ripening, especially in bananas, is largely unknown. In this study, 38 genes encoding starch degradation‐related proteins were identified and characterized from banana fruit. Expression analysis revealed that 27 candidate genes were significantly induced during banana fruit ripening, with concomitant conversion of starch‐to‐sugars. Furthermore, iTRAQ‐based proteomics experiments identified 18 starch degradation‐associated enzymes bound to the surface of starch granules, of which 10 were markedly up‐regulated during ripening. More importantly, a novel bHLH transcription factor, MabHLH6, was identified based on a yeast one‐hybrid screening using *MaGWD1* promoter as a bait. Transcript and protein levels of MabHLH6 were also increased during fruit ripening. Electrophoretic mobility shift assays, chromatin immunoprecipitation and transient expression experiments confirmed that MabHLH6 activates the promoters of 11 starch degradation‐related genes, including *Ma* *GWD* *1*,* Ma* *LSF* *2*,* Ma* *BAM* *1*,* Ma* *BAM* *2*,* Ma* *BAM* *8*,* Ma* *BAM* *10*,* Ma* *AMY* *3*,* Ma* *AMY3* *C*,* Ma* *ISA* *2*,* Ma* *ISA* *3* and *MapGlcT2‐2* by recognizing their E‐box (CANNTG) motifs present in the promoters. Collectively, these findings suggest that starch degradation during banana fruit ripening may be attributed to the complex actions of numerous enzymes related to starch breakdown at transcriptional and translational levels, and that MabHLH6 may act as a positive regulator of this process via direct activation of a series of starch degradation‐related genes.

## Introduction

Starch is the major storage carbohydrate in higher plants and is made up of glucose polymers, amylose and amylopectin, forming complex semi‐crystalline structures and starch granules in plastids (Hostettler *et al*., 2011; Monroe and Preiss, 1990). Starch degradation has been mainly investigated in *Arabidopsis* leaves where starch accumulates in chloroplasts during the day and is broken down at night to supply substrates for local use and export (Santelia *et al*., 2015). The pathway of starch degradation in *Arabidopsis* leaves at night is initiated through reversible glucan phosphorylation mediated by glucan water dikinase (GWD) and phosphoglucan water dikinase (PWD) to disrupt the semi‐crystalline starch structure at the granule surface (Hejazi *et al*., 2014; Ritte *et al*., 2006) and then the phosphoglucan phosphatases starch excess 4 (SEX4) and like‐SEX4 2 (LSF2) dephosphorylate glucans to provide access for hydrolytic enzymes to release maltose and glucose from starch. Another phosphatase, LSF1, is a putative inactive scaffold protein that may act as a regulator of starch degradative enzymes at the granule surface (Comparot‐Moss *et al*., 2010; Meekins *et al*., 2014; Silver *et al*., 2014). In parallel with the phosphorylation and dephosphorylation actions, a series of hydrolytic enzymes, including β‐amylases (BAM1/3) (Fulton *et al*., 2008), starch debranching enzymes (LSA3, LDA) (Delatte *et al*., 2005; Wattebled *et al*., 2005), disproportionating enzyme (DPE1/2) (Chia *et al*., 2004; Critchley *et al*., 2001), also digest starch to maltose and glucose. Finally, the degradation products (maltose and glucose) are transported to cytosol by the membrane channel protein Maltose Excess Protein (MEX1) and plastidic Glucose Transporter (pGlcT) (Cho *et al*., 2011; Niittylä *et al*., 2004), providing a steady supply of substrates for respiration. These findings in *Arabidopsis* leaves point out that the pathway of starch degradation is highly complex and is mediated by the changes of putative enzymes at transcript and protein levels. However, the regulation of these processes in other plant organs such as fleshy fruits is largely unknown, especially in banana fruits.

Banana is the world's most important fruit crop and one of the top 10 crops by production (Paul *et al*., 2016). It is also one of the major international trading commodities that are widely distributed throughout the world (Kuan *et al*., 2015). During fruit development, bananas accumulate large amounts of starch, with 20%–25% by fresh weight, in the pulp of the fruit (Do Nascimento *et al*., 2006). In general, bananas are harvested at mature green stage and then the ripening will be initiated by ethylene or ethylene‐releasing compounds before marketing. During ripening, the peel colour changes from green to yellow and the pulp softens (Meng and Slaughter, 1997). Fruit firmness is an important criterion for determining the edible quality and ripeness of bananas. It has been reported that coordinated degradation of pectic, hemicellulosic polysaccharides in the cell wall and starch is the main cause of banana fruit softening (Mbeguie‐A‐Mbeguie *et al*., 2009; Shiga *et al*., 2011). Starch degradation during the postharvest ripening provides carbon for the production of sucrose and flavour‐producing volatile compounds (Saraiva *et al*., 2013). To date, several genes involved in starch‐to‐sugars conversion during banana ripening have been reported, which include those encoding amylases such as MAmy (Junior *et al*., 2006), Ma‐bmy (Do Nascimento *et al*., 2006) and the starch‐debranching enzymes such as Maisa (Bierhals *et al*., 2004) and MaDEBs (Jourda *et al*., 2016). For instance, the activities of α‐amylase and β‐amylase associated with the starch granules in ripening bananas were confirmed by immunofluorescence microscopy (Peroni‐Okita *et al*., 2013). Recent analysis of gene expression suggests that β‐amylases play a key role in starch degradation in both banana species, Cavendish and Plantain (Gao *et al*., 2016). Although some genes involved in starch degradation during banana fruit ripening have been isolated, a comprehensive understanding of regulatory networks operating in this process is still lacking, especially for the regulatory mechanisms mediated by transcription factors (TFs).

Transcription factors play essential roles in gene regulation during various developmental processes including fruit ripening through their interaction with specific DNA sequence motifs in the promoters of target genes. For examples, MaERF9/11 regulates ethylene biosynthetic genes *MaACS1* and *MaACO1* in banana (Xiao *et al*., 2013), ripening inhibitor RIN directly binds to the promoters of miRNAs during tomato fruit development and ripening (Gao *et al*., 2015), and MdERF2 negatively affects ethylene biosynthesis and fruit ripening by suppressing the transcription of *MdACS1* via multiple mechanisms in apple (Li *et al*., 2016). Several TFs have been reported to be involved in fruit softening. In tomato, RIN governs fruit ripening, at least partially, by regulating genes involved in cell wall degradation (Irfan *et al*., 2016; Kumar *et al*., 2012). In kiwifruit, AdEILs and AdERFs, two TFs in the ethylene signalling pathway, control fruit ripening via transcriptional regulation of cell wall modifying genes (Yin *et al*., 2010). Recently, MaERF11 was found to be involved in banana ripening by modulating *MaEXP2/7/8* transcription (Han *et al*., 2016), and the overexpression of the ABA‐Stress‐Ripening (ASR) TF promoted tomato fruit softening and ripening (Jia *et al*., 2016). These findings indicate that TFs affect fruit softening by regulating cell‐wall modifying enzyme genes. However, little is known about TFs involved in controlling genes related to starch degradation during fruit ripening.

In the present work, 38 genes encoding starch degradation enzymes were identified from banana fruit, of which 27 genes were up‐regulated during fruit ripening paralleling starch degradation and the accumulation of soluble sugars. Moreover, iTRAQ‐based proteomics experiments revealed that 18 starch degradation‐associated enzymes were bound to the surface of starch granules, of which 10 showed increased amounts during fruit ripening. More importantly, we also identified a positive transcriptional activator, MabHLH6, that binds to and activates the promoters of 11 starch degradation‐related genes. These findings advance the understanding of complex regulatory networks of starch degradation, a critical process of fruit ripening and fruit quality in banana.

## Results

### Change in ripening parameters in bananas with three different ripening behaviours

**Figure 1:**
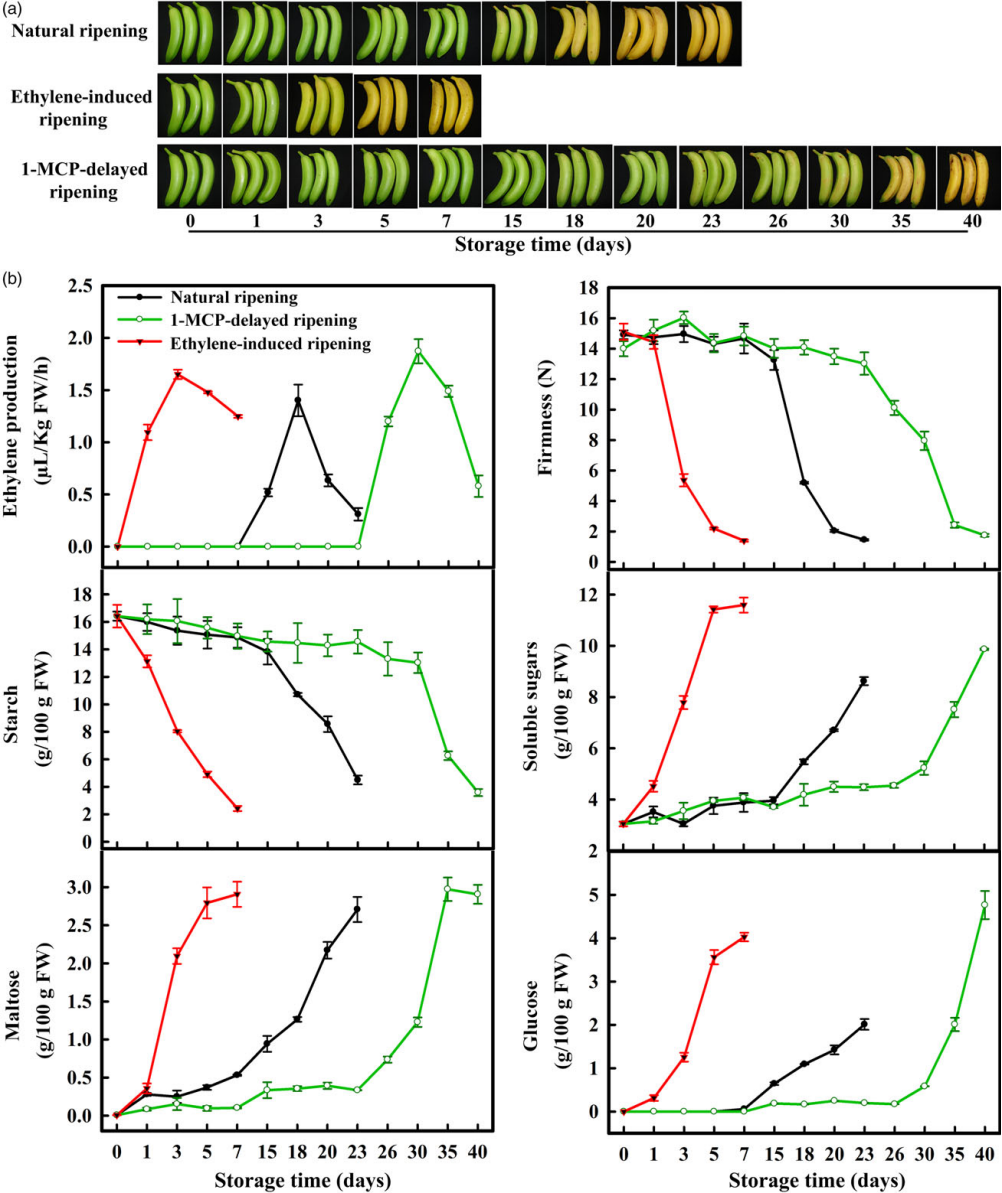
(a) Bananas with three different ripening behaviours: natural (control), ethylene‐induced, and 1‐MCP‐delayed ripening. (b) Changes in fruit firmness, ethylene production and contents of starch, total soluble sugars, maltose and glucose in pulp during banana fruit ripening. Each value represents the mean ± SE of three replicates.

The important ripening parameters such as ethylene production and fruit firmness were measured in banana fruit under natural, ethylene‐induced and 1‐MCP‐delayed ripening (Figure 1a). As shown in Figure 1b, ethylene production in natural ripening fruit was increased significantly after the storage for 15 days, reaching a maximum value on day 18 and declined thereafter. The pulp firmness began to decline on the 18th day of storage, with firmness reaching a minimum value of ~2 N on day 23. Ethylene treatment hastened banana fruit ripening, as evidenced by the early appearance of ethylene production on day 1 and sharp decrease in pulp firmness on day 3. By contrast, treatment of 1‐MCP delayed ripening and ethylene production, with firmness declining and ethylene production peaking on day 30 (Figure 1b).

Starch‐to‐sugars conversion during banana fruit ripening is associated with the transformation of inedible‐to‐edible. Maltose and glucose are the products of starch degradation. Accordingly, the starch content of natural ripening fruit was decreased by day 15, with the content declining to 4% by day 23 and a corresponding increase in soluble sugar content (Figure 1b). In ethylene‐treated and 1‐MCP‐treated fruits, the decrease in starch content and increase in soluble sugars (maltose and glucose) showed similar patterns paralleling their ripening behaviour (Figure 1b). Overall, these data indicate that the conversion of starch‐to‐sugars is in good agreement with fruit softening and ethylene production during banana ripening.

### Morphology of starch granules in unripe and ripe banana fruits

**Figure 2:**
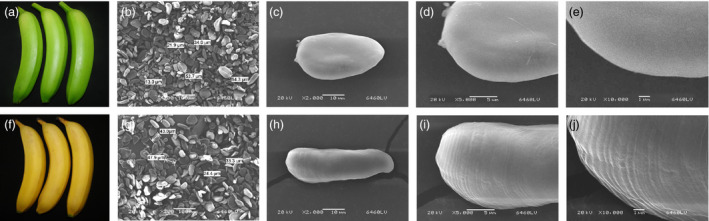
(a, f) Unripe and ripe banana fruits. (b–e) High‐magnification SEM image of starch granules isolated from the unripe bananas. (g‐j) High‐magnification SEM image of starch granules from ripe bananas.

As presented in Figure 2, the scanning electron microscopy (SEM) assays showed clear differences in shape and size of starch granules in unripe and ripe banana fruits. The starch granules in green bananas had a smooth surface (Figure 2c–e), while those of ripe bananas exhibited distinct parallel grooves indicating starch degradation on their surfaces (Figure 2h–j).

### Expression patterns of starch degradation‐related genes in pulp during banana fruit ripening

**Figure 3:**
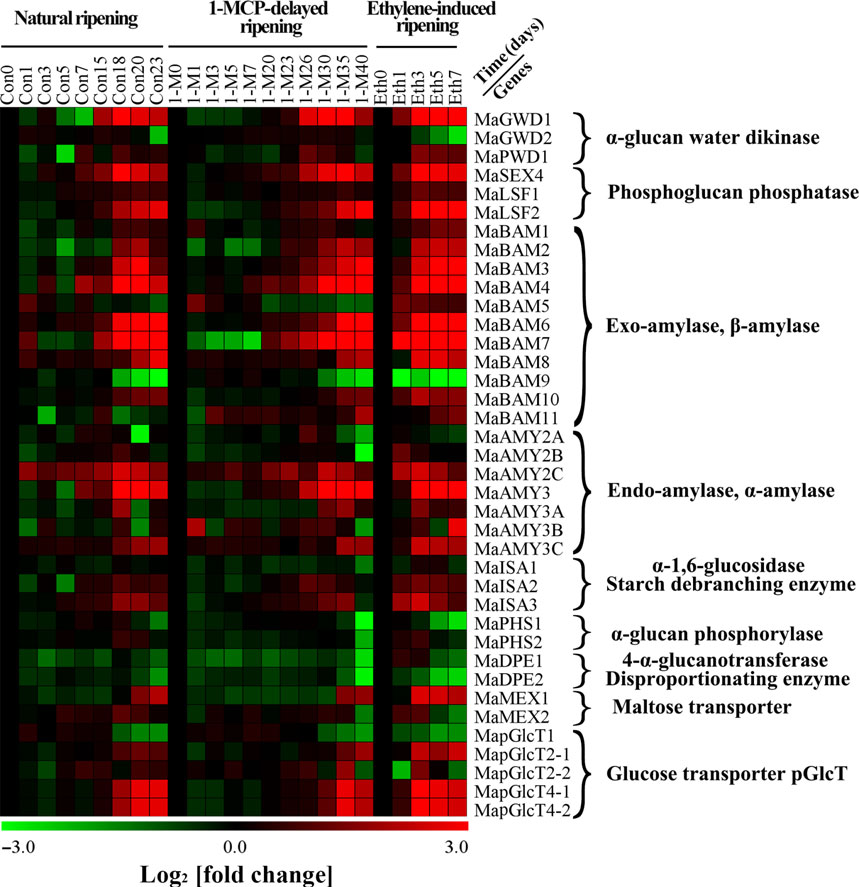
Multiple array analysis of 38 starch degradation‐related genes in banana fruit pulp with three different ripening characteristics, including natural (control), ethylene‐induced, and 1‐MCP‐delayed ripening. Data are log_2_‐transformed value of gene expression of each time point compared to day 0 of control fruit. The expression image was generated using MeV software and the scale indicates log2 variations: red, increase; green, decrease. Means ± SE from three repeats are provided in Figure S1.

Based on the banana genome sequence (http://banana-genome.cirad.fr/) and the NCBI database (ftp://ftp.ncbi.nih.gov/genomes/Musa_acuminata/), 38 genes encoding starch degradation‐related enzymes were identified and characterized. These include the gene encoding α‐glucan water dikinases (*MaGWD1*,* MaGWD2*, and *MaPWD1*), phosphoglucan phosphatases (*MaSEX4*,* MaLSF1*, and *MaLSF2*), β‐amylase (*MaBAM1‐MaBAM11*), α‐amylases (*MaAMY2A*,* MaAMY2B*,* MaAMY2C*,* MaAMY3*,* MaAMY3A*,* MaAMY3B*, and *MaAMY3C*), starch debranching enzymes (*MaISA1‐MaISA3*), α‐glucan phosphorylases (*MaPHS1* and *MaPHS2*), disproportionating enzymes (*MaDPE1* and *MaDPE2*), maltose excess proteins (*MaMEX1* and *MaMEX2*) and plastidic glucose transporters (*MapGlcT1*,* MapGlcT2‐1*,* MapGlcT2‐2*,* MapGlcT4‐1* and *MapGlcT4‐2*). The banana genome IDs and the NCBI accession numbers of these genes are shown in Table S1. It should be pointed out that 15 genes are firstly reported in our works, which include *MaGWD1*,* MaGWD2*,* MaPWD1*,* MaSEX4*,* MaLSF1*,* MaLSF2*,* MaDPE1*,* MaDPE2*,* MaMEX1*,* MaMEX2*,* MapGlcT1*,* MapGlcT2‐1*,* MapGlcT2‐2*,* MapGlcT4‐1* and *MapGlcT4‐2* (Table S1). As shown in Figure 3 and Figure S1, during ripening in all three different treatments, transcripts of 27 genes (*MaGWD1*,* MaPWD1*,* MaSEX4*,* MaLSF1*,* MaLSF2*,* MaBAM1‐MaBAM4*,* MaBAM6‐MaBAM8*,* MaBAM10*,* MaAMY2B*,* MaAMY2C*,* MaAMY3*,* MaAMY3A*,* MaAMY3C*,* MaISA2*,* MaISA3*,* MaPHS2*,* MaMEX1*,* MaMEX2*,* MapGlcT2‐1*,* MapGlcT2‐2*,* MapGlcT4‐1* and *MapGlcT4‐2*) were dramatically increased with concomitant increase in ethylene production, while transcripts of *MaGWD2*,* MaAMY2A*,* MaBAM9*,* MaPHS1*,* MaDPE1*,* MaDPE2* and *MapGlcT1* were decreased (Figure 3 and Figure S1). However, 3 genes (*MaAMY3B*,* MaBAM5* and *MaBAM11*) had irregular changes and one gene (*MaISA1*) displayed no obvious variation in its activity.

### Identification of starch degradation‐related proteins bound to the starch granules’ surface and their accumulations by iTRAQ

**Figure 4:**
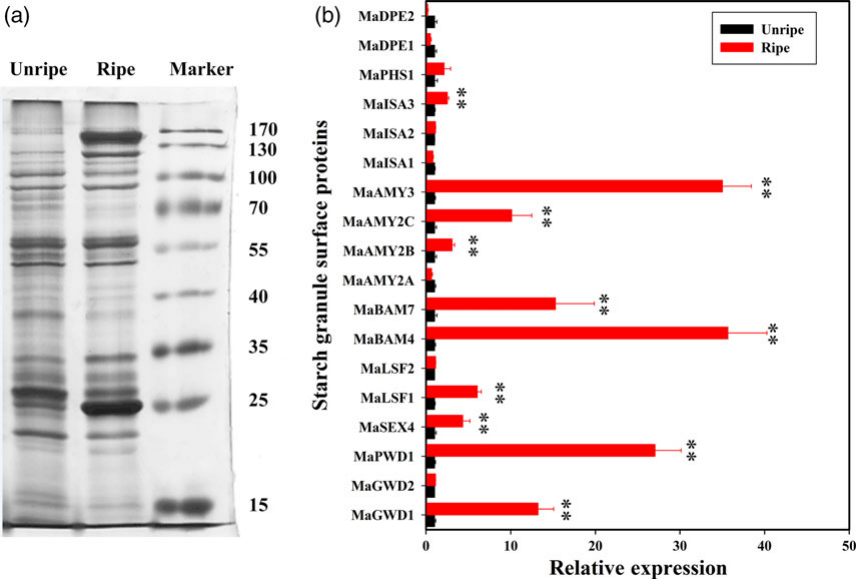
(a) SDS‐PAGE (7.5%) gel of proteins bound to the starch granule surface. The gel was stained with silver reagent, and the molecular‐weight markers ranged from 15 to 170 kDa. Unripe: starch‐bound protein extracted from banana fruit at day 0. Ripe: bananas starch bond protein extracted from banana fruit on day 3 of ethylene treatment. (b) iTRAQ showing that protein accumulations on the starch granule surface were differentially expressed in ripe and unripe banana fruits. The relative level of each protein was shown as a ratio relative to 0 day of control, which was set as 1. Each value represents the mean ± SE of three replicates. ** represents significant differences in *P* values <0.01.

**Figure 5:**
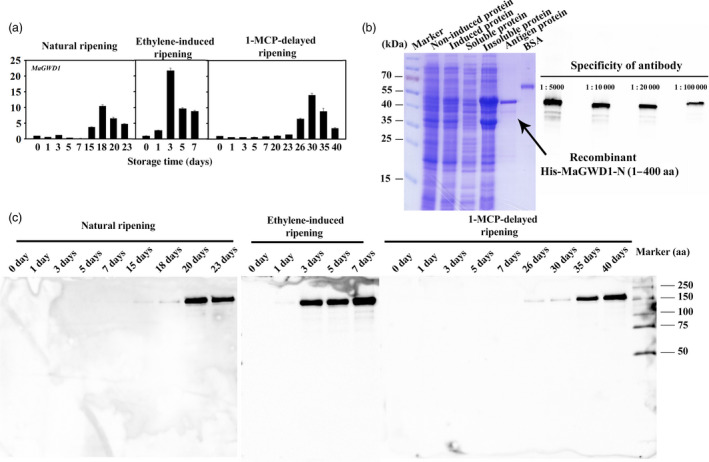
Accumulation of the MaGWD1 transcript and protein during banana fruit ripening. (a) The transcript levels of *MaGWD1* are expressed as a ratio relative to the harvest time (0 day of control), which was set as 1. Each value represents the mean ± SE of three replicates. (b) Preparation of polyclonal antibody against MaGWD1 and specificity test of the antibody by Western blot with four antibody gradient concentrations ranging from 1 : 5000 to 1 : 100 000. (c) Western blot analysis of protein level of MaGWD1 from the starch granule surface in banana fruits with three different ripening behaviours. Equal weight of starch was used to normalize the loading proteins.

To examine the changes of starch granule‐bound proteins during banana ripening, they were isolated from the starch granule of unripe and ripe banana fruits (Figure 4a) and then assayed using an iTRAQ‐based quantitative proteomics approach. As shown in Figure 4b, 18 starch degradation‐related candidate proteins were identified on the starch granule surface. Among these, MaGWD1, MaPWD1, MaSEX4, MaLSF1, MaBAM4, MaBAM7, MaAMY2B, MaAMY2C, MaAMY3 and MaISA3 accumulated considerably in ripe fruits paralleling their transcript levels except for MaGWD2 and MaPHS1. MaAMY2A, MaISA1, MaDPE1 and MaDPE2 were down‐regulated, while MaGWD2, MaLSF2, MaISA2 and MaPHS1 changed slightly. As AtGWD1 has been suggested to play an important role in transitory starch degradation in *Arabidopsis* leaves (Hejazi *et al*., 2014; Ritte *et al*., 2006), MaGWD1, the homologue of AtGWD1 (Figure S2) was selected for further investigation. Western blotting analysis using MaGWD1 antibody with good specificity (Figure 5b) showed that MaGWD1 protein was induced dramatically in the ripening stage of bananas with natural, ethylene‐induced or 1‐MCP‐delayed ripening (Figure 5c). This corresponds well with its transcript level (Figures 3 and 5a).

### Identification of a *MaGWD1* promoter‐interacting protein MabHLH6

**Figure 6:**
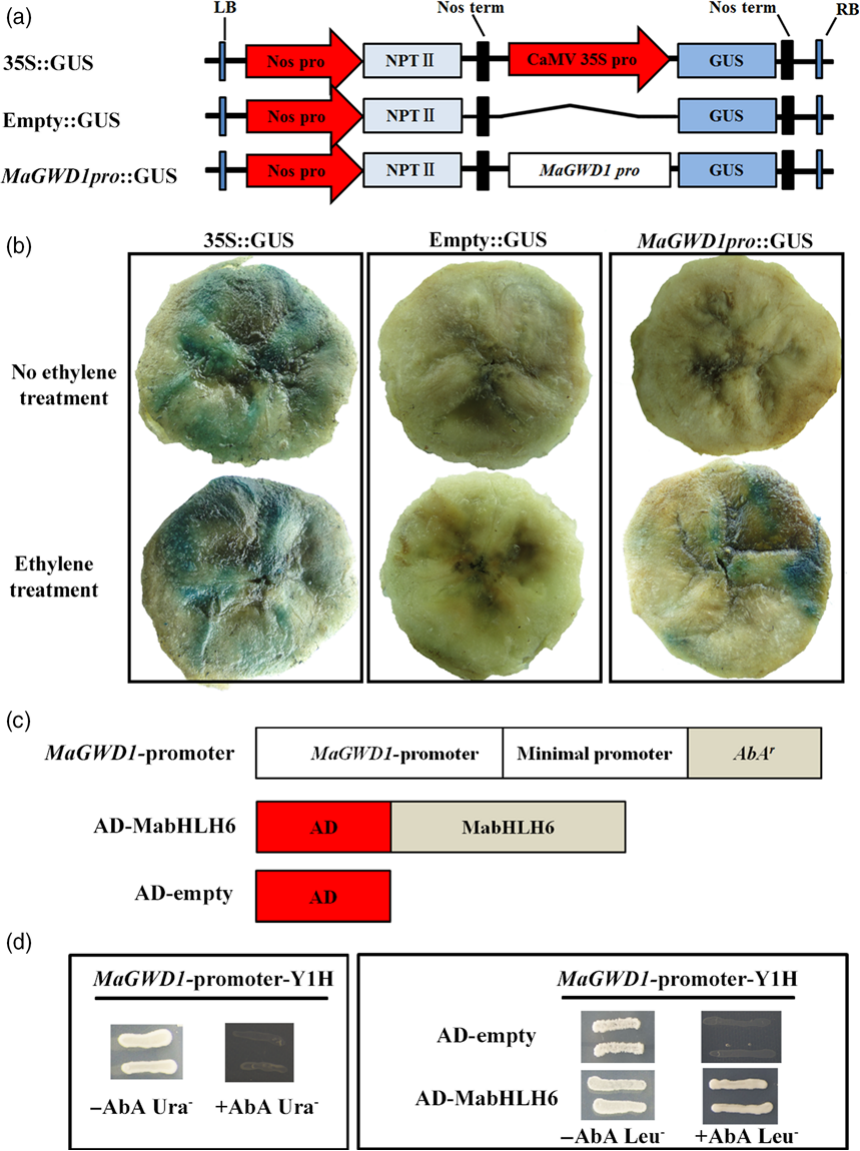
*MaGWD1* promoter activity in response to ethylene and binding of MabHLH6 to *MaGWD1* promoter by Yeast one‐hybrid assay. (a) GUS reporter constructs containing the *MaGWD1* promoter (*MaGWD1* pro::GUS), the CaMV 35S promoter (35S::GUS, positive control) and the GUS without promoter (empty::GUS, negative control). (b) Three reporters were transiently transformed into banana pulp using *Agrobacterium*‐mediated method. Transfected bananas were subjected to 0 (control) or 0.2% (v/v) ethrel (ethylene releaser) treatment and incubated at 23 °C for 60 h. GUS activity was localized histochemically by placing sliced banana pulp in the appropriate staining buffer containing 0.5 mm X‐Gluc and incubating in the dark at 37 °C for 12‐24 h. The tissue was decolourized using 70% ethanol for 1 h for clear visualization. (c) Schematic representation of the plasmids used in the yeast one‐hybrid (Y1H) assay. (d) Y1H analysis of MabHLH6 binding to *MaGWD1* promoters. Left: No basal activities of MaGWD1 promoter were detected in yeast grown on SD medium lacking Leu in the presence of aureobasidin A (AbA). Right: Yeast growth assay after the Y1H reporter strains was transformed with plasmids carrying cassettes constitutively expressing MabHLH6 effectors. Interaction was determined based on the ability of the transformed yeast to grow on SD medium lacking Leu in the presence of AbA.

Based on the transcript and protein expression of MaGWD1 during ripening, it seems that like AtGWD1, MaGWD1 might also play an important role in banana fruit starch degradation. To investigate the potential regulators of *MaGWD1*,* MaGWD1* promoter was isolated and its expression pattern was tested using a GUS reporter (Figure 6a) in a transient expression assay. As shown in Figure 6b, in banana pulp transfected with GUS reporter driven by the *MaGWD1* promoter, GUS signals were observed in pulp with ethylene treatment. As a positive control (35S::GUS), GUS signals were constitutively expressed in banana pulp with or without ethylene treatment, while no GUS signal was detected in negative control (empty::GUS) (Figure 6b).

Then, we performed a yeast one‐hybrid library screening using the *MaGWD1* promoter as a bait and a banana ripening associated cDNA library as prey. After high‐stringency screening, a cDNA encoding a bHLH TF was identified and was designated as MabHLH6 (XP_009408340.1) after the names of previous five *bHLH* genes (Peng *et al*., 2013). The interaction between MabHLH6 and *MaGWD1* promoter was further confirmed by yeast one‐hybrid assay (Figure 6c,d).

### Molecular characterization of *MabHLH6*

**Figure 7:**
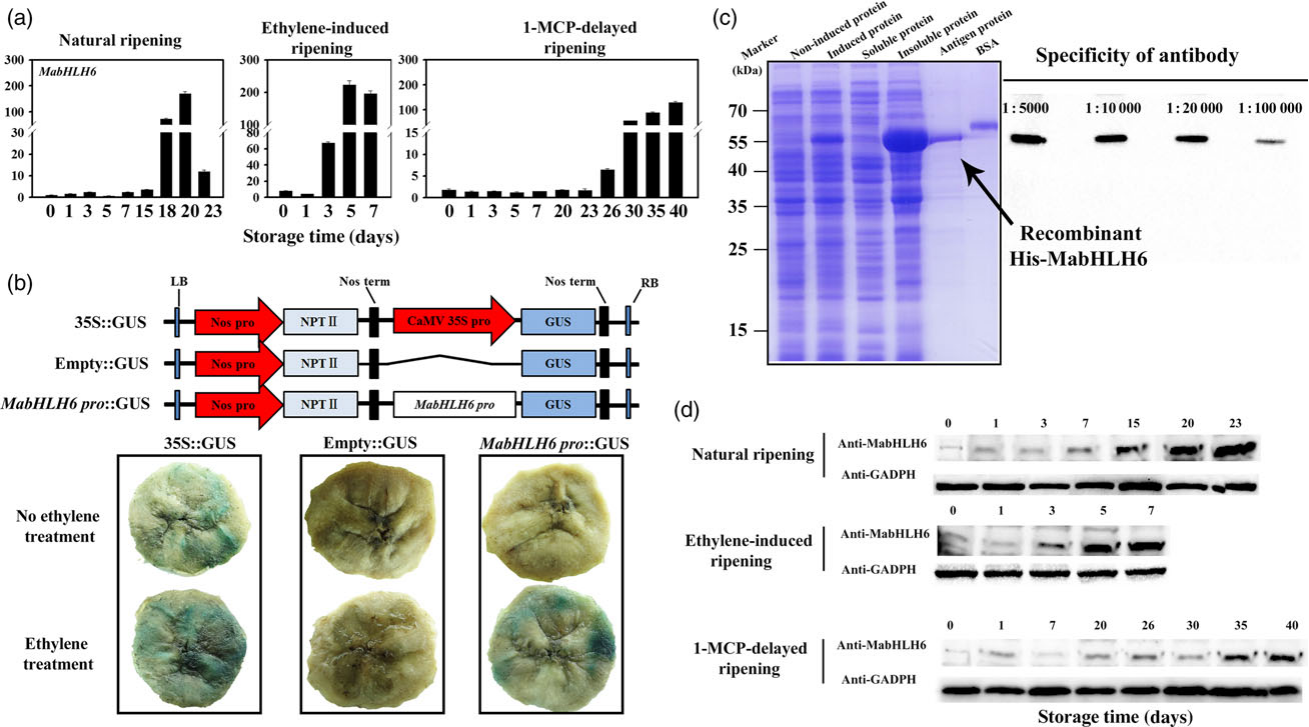
Gene expression and protein accumulation of MabHLH6 in banana fruit pulp with three different ripening behaviours and its promoter activity. (a) The transcript levels of *MabHLH6* are expressed as a ratio relative to the harvest time (0 day of control), which was set as 1. Each value represents the mean ± SE of three replicates. (b) MabHLH6 promoter activity in response to ethylene. GUS reporter constructs containing the MabHLH6 promoter (MabHLH6 pro::GUS), the CaMV35S promoter (35S::GUS, positive control) and the GUS without promoter (empty::GUS, negative control) were transiently transformed into different ripening banana pulp and subjected to ethrel treatment as described above, as well as GUS visualization. (c) Preparation of polyclonal antibody against MabHLH6 and the detection of specificity of antibody by Western blot with four antibody gradient concentrations ranging from 1 : 5000 to 1 : 100 000. (d) Western blot analysis of protein level of MabHLH6 in bananas with three different ripening behaviours. Anti‐GADPH antibody was used as a reference control to normalize the loading proteins.

The *MabHLH6* cDNA contains an Open Reading Frame (ORF) of 1437 bp in length, encoding a polypeptide of 478 amino acids with a calculated molecular weight of 51.84 kDa and a predicted isoelectric point (*p*I) of 6.40. MabHLH6 was clustered with AtbHLH62 and AtbHLH78 into GBOF subfamily (Figure S3) and consists of a typical helix‐loop‐helix domain (Figure S4) as other bHLH proteins (Buck and Atchley, 2003). As a TF, MabHLH6 protein is localized in the nucleus and exhibited transcriptional activation activity in tobacco leaf and yeast (Figures S5 and S6). Similarly to *MaGWD1*, the expression of *MabHLH6* showed strong positive correlation with starch degradation and fruit ripening, with highest levels at day 18 (~170‐fold), day 5 (~223‐fold) and day 40 (~128‐fold) in natural, ethylene‐induced and 1‐MCP‐delayed ripening bananas, respectively (Figure 7a). Meanwhile, its promoter activity (via GUS reporter) was induced by ethylene in a transient expression assay (Figure 7b). Furthermore, Western blotting using specific anti‐MabHLH6 antibody (Figure 7c) indicated that MabHLH6 protein was also increased at the ripening stage, paralleling the decrease in pulp firmness and starch content (Figure 7d).

### MabHLH6 activates the expression of genes encoding starch degradation‐related enzymes by directly binding to their promoters

**Figure 8:**
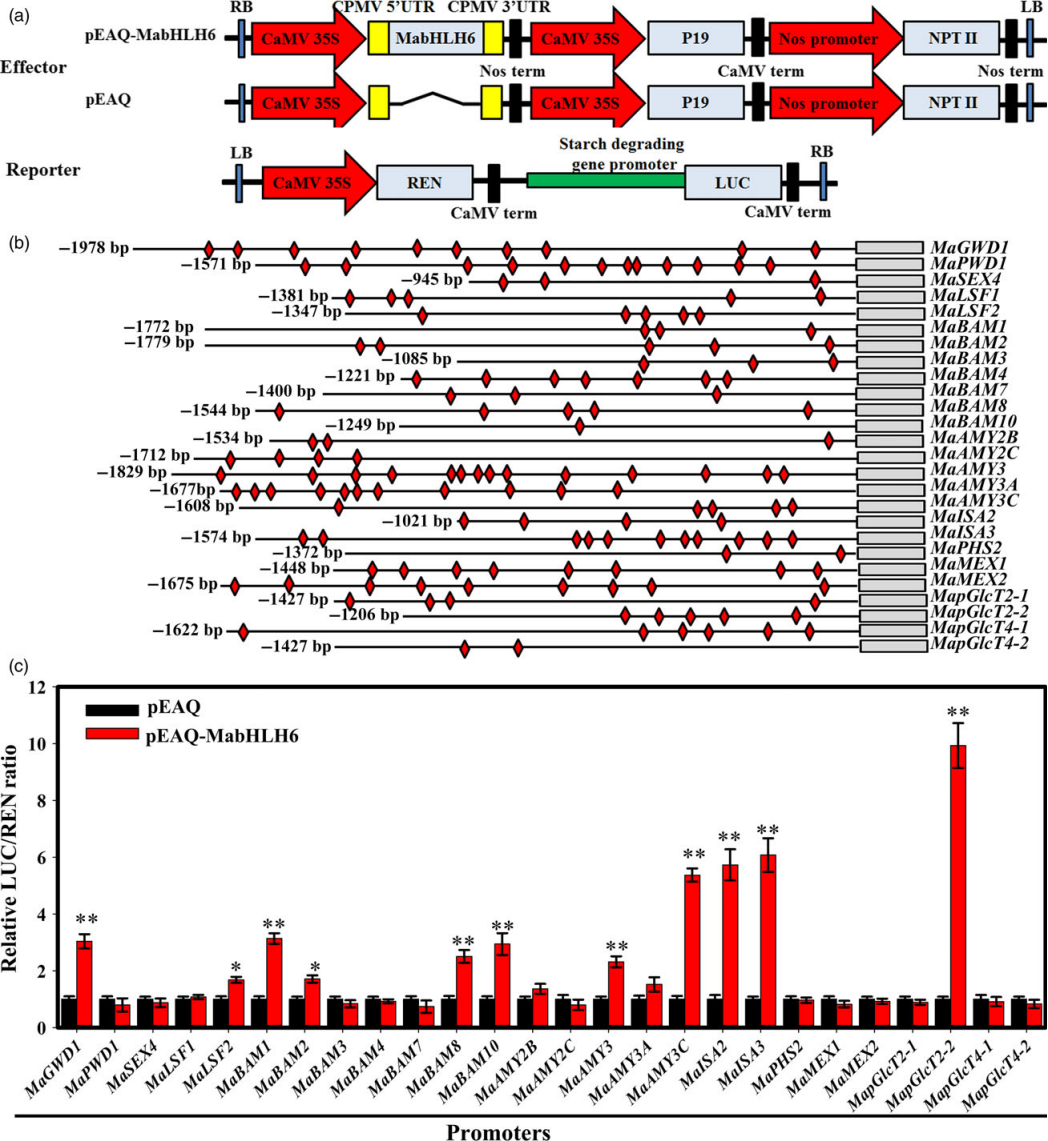
MabHLH6 activates the expression of genes involved in starch degradation. (a) Diagrams of the reporter and effector constructs used in the dual‐luciferase reporter assay. (b) Schematics of the 26 promoters of starch degradation‐related genes. Promoter length and E‐box motifs are indicated with lines and red diamonds, respectively. (c) *In vivo* interactions of MabHLH6 with the promoters obtained from transient assays in tobacco leaves. The ratio of LUC/REN of the empty vector (pEAQ) plus promoter was used as a calibrator (set as 1). Each value represents the means of six biological replicates, and vertical bars represent the SE * and ** represent significant differences in *P* values <0.05 and 0.01, respectively.

It is well‐known that bHLH TFs preferentially bind to the E‐box (CANNTG) motifs of their target promoters (Ji *et al*., 2014). As 27 genes (*MaGWD1*,* MaPWD1*,* MaSEX4*,* MaLSF1*,* MaLSF2*,* MaBAM1‐MaBAM4*,* MaBAM6‐MaBAM8*,* MaBAM10*,* MaAMY2B*,* MaAMY2C*,* MaAMY3*,* MaAMY3A*,* MaAMY3C*,* MaISA2*,* MaISA3*,* MaPHS2*,* MaMEX1*,* MaMEX2*,* MapGlcT2‐1*,* MapGlcT2‐2*,* MapGlcT4‐1* and *MapGlcT4‐2*) were up‐regulated during banana ripening (Figure 3 and Figure S1), their promoters were obtained except for *MaBAM6* due to failure of its promoter isolation, and the sequence analysis identified E‐box (CANNTG) motifs (DataS1). To test the capacity of MabHLH6 to regulate these promoters, transient assays were performed. For these analyses, tobacco leaves were co‐transformed with LUC reporter plasmids under the control of the promoters of starch degradation‐associated genes together with an overexpression vector carrying *MabHLH6* under the control of the CaMV 35S promoter (Figure 8a). As shown in Figure 8c, among the 26 promoters tested, activities of *MaGWD1*,* MaLSF2*,* MaBAM1*,* MaBAM2*,* MaBAM8*,* MaBAM10*,* MaAMY3*,* MaAMY3C*,* MaISA2*,* MaISA3* and *MapGlcT2‐2* promoters were significantly induced in the presence of MabHLH6, with relatively higher LUC/REN ratio compared to that of the control.

**Figure 9:**
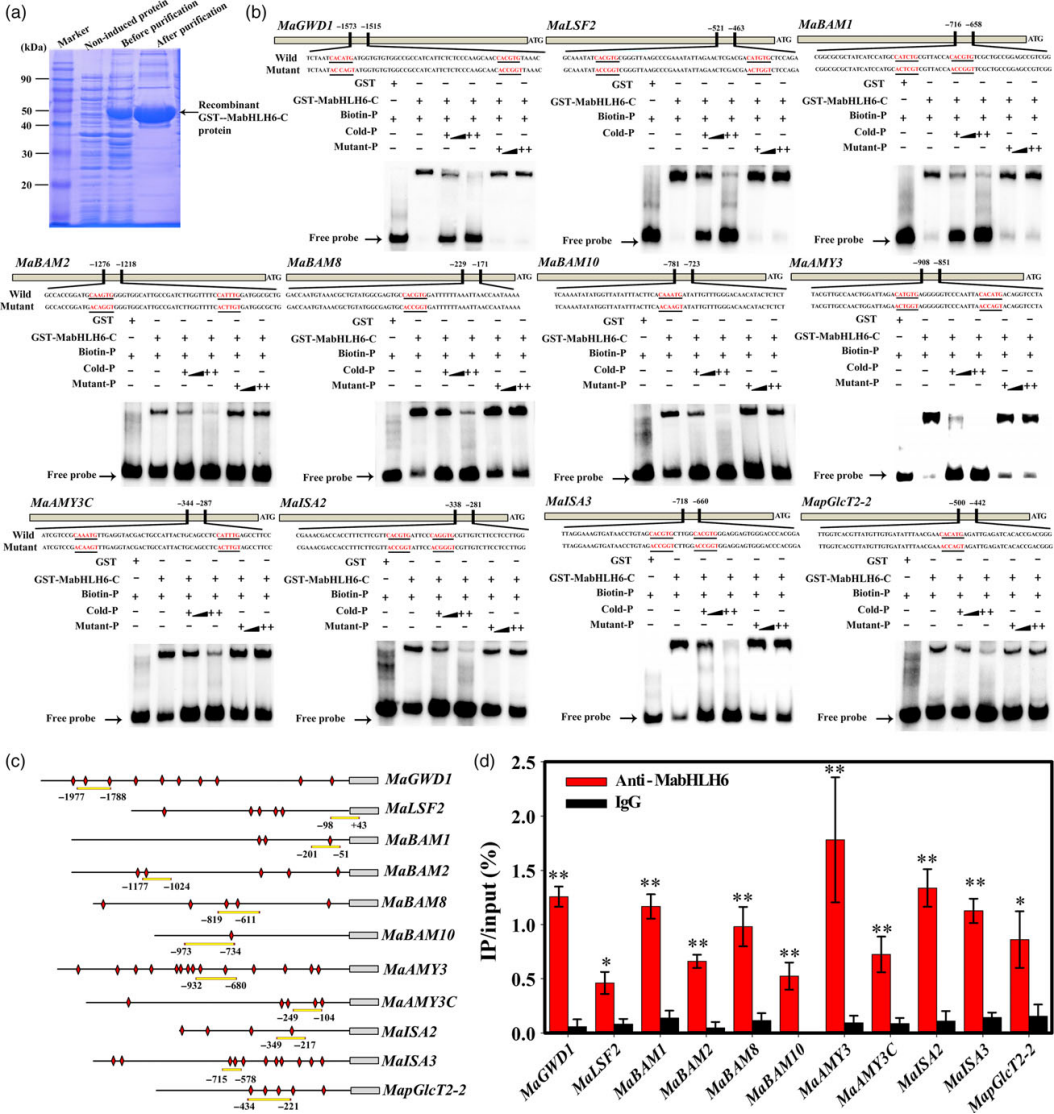
MabHLH6 binds to the promoters of 11 starch degradation‐related genes *in vitro* and *in vivo*. (a) SDS‐PAGE gel stained with coomassie brilliant blue demonstrating affinity purification of the recombinant GST‐MabHLH6‐C protein used for the EMSA assay. (b) EMSA showing MabHLH6 binding to the promoters of starch degradation‐related genes containing E‐box element. The probe sequences corresponding to each of the target gene promoters are shown, with red letters representing the E‐box and the mutant E‐box. The purified GST or recombinant GST‐MabHLH6‐C protein was mixed with probes, and the protein–DNA complexes were separated on native polyacrylamide gels. Triangles indicate increasing amounts of unlabelled or mutated probes for competition. (c) Schematic representation of selected regions for ChIP‐qPCR analyses. E‐box motifs and probes used for ChIP‐qPCR assay are indicated with red diamonds and yellow underlines, respectively. (d) ChIP‐qPCR assay showing the binding of MabHLH6 to the promoters of starch degradation‐associated genes. DNA samples were immunoprecipated with MabHLH6 antibody or IgG control. * and ** indicate significant differences in *P* values <0.05 and 0.01, respectively.

To further study whether MabHLH6 binds directly to the *MaGWD1*,* MaLSF2*,* MaBAM1*,* MaBAM2*,* MaBAM8*,* MaBAM10*,* MaAMY3*,* MaAMY3C*,* MaISA2*,* MaISA3* and *MapGlcT2‐2* promoters, electrophoretic mobility‐shift assays (EMSA) were conducted. As shown in Figure 9a,b, the recombinant MabHLH6 protein was able to bind to these 11 promoter fragments and caused mobility shifts, and the shifted bands were disappeared by the addition of increasing amounts of unlabeled competitors with the same sequence, but not by the mutated probes. The binding of MabHLH6 with these promoters *in vivo* were further verified by chromatin immunoprecipitation‐quantitative PCR (ChIP‐qPCR) analysis using polyclonal anti‐MabHLH6 antibody. As expected, the promoter regions containing the E‐box of *MaGWD1*,* MaLSF2*,* MaBAM1*,* MaBAM2*,* MaBAM8*,* MaBAM10*,* MaAMY3*,* MaAMY3C*,* MaISA2*,* MaISA3* and *MapGlcT2‐2* promoters were significantly enriched by the anti‐MabHLH6 compared with the negative control IgG (Figure 9c,d). Together, these data illustrate that MabHLH6 may act as a positive transcriptional activator of a subset of starch degradation related‐genes by directly binding to E‐box motifs of their promoters.

## Discussion

Softening and sweetening are two critical phenotypic indicators of banana fruit ripening, which are at least partially due to the degradation of starch. Bananas accumulate a large quantity of starch during fruit growth and development, and this starch abundance maintains firmness in unripe bananas. During ripening, the decline of pulp firmness correlates well with starch degradation (Kojima *et al*., 1994; Shiga *et al*., 2011). Our works also showed that the starch content decreases during banana fruit ripening, which is strongly related to the reduction in pulp firmness and the appearance of soluble sugars (Figure 1). Starch degradation occurs via a network of reactions rather than a linear pathway, which has been extensively studied in *Arabidopsis* leaves and cereal endosperms (Zeeman *et al*., 2010). In *Arabidopsis thaliana*, actions of glucan phosphorylation, glucan partial hydrolysis, phosphoglucan dephosphorylation, glucan complete hydrolysis and product export are mediated by a series of enzymes, including GWD1, PWD, BAM1/3, AMY3, ISA3, SEX4, LSF1/2, DPE1, PHS1, MEX1 and pGlcT (Blennow and Engelsen, 2010; Cho *et al*., 2011; Critchley *et al*., 2001; Edner *et al*., 2007; Kötting *et al*., 2005, 2009; Scheidig *et al*., 2002; Silver *et al*., 2014; Wattebled *et al*., 2005; Weise *et al*., 2005; Zeeman *et al*., 2004). In this study, we isolated and characterized 38 genes encoding starch breakdown‐related enzymes from banana fruit (Table S1), of which some were ethylene inducible at both transcriptional and translational levels (Figures 3 and 4). This paralleled the decrease in starch content and increase in total soluble sugars during ripening (Figure 1). Moreover, SEM analysis indicated clear change in starch granules morphology from oval with smooth surface to elongated with parallel grooves (Figure 2), indicating the enzymatic effects caused by fruit ripening. Our results, together with previous findings, provide evidence that transcriptional and translational changes of numerous starch breakdown‐related enzymes account for the degradation of starch. It should be noted that starch degradation in bananas, contrary to the previous findings from *Arabidopsis* leaves and cereal endosperms, may be depended on ethylene. Our results showed that the expression of genes associated with starch degradation were all increased by ethylene treatment and during fruit ripening (Figure 3), with a parallel reduction of starch content and increased soluble sugars in bananas (Figure 1). Based on the expression profile analysis, *MaGWD1*,* MaPWD1*,* MaISA1*,* MaSEX1*,* MaAMY1*,* MaAMY2* and *MaBAM4* may have a crucial role in the early phase of starch degradation, while *MaLSF1*,* MaBAM1*,* MaBAM2* and *MaBAM3* may be important in the late phase of the process (Figure 3). Interestingly, in apple, the role of ethylene in starch degradation differs between cultivars, as ethylene is partially involved in starch degradation in mature ‘Tsugaru’, but not in ‘Fuji’ (Thammawong and Arakawa, 2007). Similarly, various banana cultivars display distinct ripening behaviours and different patterns of starch metabolism, which determines the habit of its consumption. For example, dessert bananas with high amount of soluble sugars are usually consumed as fresh fruit, while plantains with lots of undigested starch are used as cooking bananas (Shiga *et al*., 2011). Therefore, it is interesting to investigate the expression of those genes involved in starch degradation in different cultivars of bananas during the ripening process in future.

In *Arabidopsis thaliana*, AtGWD1 was known to initiate the breakdown of starch through reversible glucan C‐6 phosphorylation to disrupt the semi‐crystalline starch structure at the granule surface (Edner *et al*., 2007; Silver *et al*., 2014); then, PWD enzyme executes C‐3 phosphorylation at these sites inducing local amorphization permitting hydrolytic cleavage by hydrolases such as β‐amylase (BAM) and isoamylase (ISA) (Blennow and Engelsen, 2010; Kötting *et al*., 2005). These findings suggest that GWD is critical for glucan phosphorylation during starch degradation, and hence, GWD is a good candidate for starch biotechnology strategies, as decreasing its activity can enhance starch contents and inhibit unwanted starch degradation, while increasing its activity could elevate granule‐bound phosphate content (Santelia and Zeeman, 2011). In the current work, iTRAQ and immuno‐blotting analyses of starch‐related proteins showed that MaGWD1, homologous of AtGWD1 (Figure S2), was present on the surface of starch granules and increased its content in the pulp of ripe bananas (Figure 5). Moreover, ethylene induced the activity of *MaGWD1* promoter (Figure 6b), indicating that MaGWD1 may play an important role in ethylene‐mediated starch breakdown.

Understanding the regulation of starch degradation during ripening of starchy fruits like bananas has practical implications, and several factors (proteins) participating in this process have been reported. For instance, in barley, several positive regulators such as RAMY (a zinc‐finger transcription factor), SAD (a DOF transcription factor), HvGAMYB and HvMYBS3 bind to the same *cis*‐acting elements of *Amy32b* gene in the presence of GA to form an ‘enhanceosome’ (Rubio‐Somoza *et al*., 2006a,b; Zou *et al*., 2008). *Poncirus trifoliate* PtrBAM1 which plays a role in cold tolerance by catalysing starch to soluble sugars was shown to be regulated by a member of the CBF regulon (Peng *et al*., 2014). In this work, we used *MaGWD1* promoter for yeast one‐hybrid screening, and identified a bHLH TF, MabHLH6, which binds specifically to *MaGWD1* promoter (Figure 6). MabHLH6 expression and protein accumulation were found to be enhanced markedly by ethylene and fruit ripening (Figure 7). More importantly, further experiments revealed that MabHLH6 could bind directly, *in vivo* and *in vitro*, not only to the promoter of *MaGWD1*, but also to those of other starch degradation‐related genes including *MaLSF2*,* MaBAM1*,* MaBAM2*,* MaBAM8*,* MaBAM10*,* MaAMY3*,* MaAMY3C*,* MaISA2*,* MaISA3* and *MapGlcT2‐2*, via the E‐box motifs, and activate their activities (Figures 8 and 9). Although bHLH TFs were reported to function in various biological processes, such as plant growth and development (Yadav *et al*., 2005), abiotic stresses responses (Peng *et al*., 2013) and flavonoid synthesis (Xu *et al*., 2015), few cases have been reported for their role in starch degradation. To the best of our knowledge, the current study is the first report of bHLH TFs involved in starch degradation via controlling a series of starch degradation‐related genes.

In summary, a total of 38 genes that encode starch degradation‐related proteins were isolated and characterized from banana fruit. Among these genes, most were increased dramatically at both transcriptional and translational levels, which are in good agreement with the decrease in starch content and increase in total soluble sugars during banana fruit ripening. Moreover, a bHLH TF, termed MabHLH6, was identified, and its expression and protein accumulation were markedly induced by ethylene and ripening. More interestingly, MabHLH6 activated *MaGWD1, MaLSF2*,* MaBAM1*,* MaBAM2*,* MaBAM8*,* MaBAM10*,* MaAMY3*,* MaAMY3C*,* MaISA2*,* MaISA3* and *MapGlcT2‐2* expressions via directly binding to their promoters. Taken together, our findings demonstrate that the complex regulatory network of starch degradation in banana fruit was mediated by the action of multiple genes and/or proteins related to starch degradation, possibly controlled by ethylene, and that MabHLH6 may act as a transcriptional activator involved in starch‐to‐sugars conversion by directly regulating starch degradation‐related genes. Our works provide new insights into the regulatory network of starch degradation during banana fruit ripening.

## Experimental procedures

### Plant materials and treatments

Preclimacteric banana (*Musa acuminata*, AAA group, cv. Cavendish) fruits at 75%–80% maturation were harvested from a local commercial plantation near Guangzhou, China. Three postharvest treatments, including a control (natural ripening), ethylene‐induced ripening (100 μL/L ethylene, 18 h), 1‐MCP‐delayed ripening (0.5 μL/L 1‐MCP, 18 h), were performed as described previously (Shan *et al*., 2012). After each treatment, fruit were held at 22 °C and 90% relative humidity until completely ripened. At each sampling time, ethylene production and fruit firmness were recorded. All of the samples were frozen in liquid nitrogen and stored at −80 °C for further use.

### Measurement of starch, soluble sugar, maltose and glucose content

The total starch in the banana pulp was quantified enzymatically using the Starch Assay Kit (Sigma‐Aldrich Shanghai Trading Co Ltd, Shanghai, China). Glucose is phosphorylated by adenosine triphosphate (ATP) in the reaction catalysed by hexokinase. Glucose‐6‐phosphate (G6P) is then oxidized to 6‐phosphogluconate in the presence of nicotinamide adenine dinucleotide (NAD) in a reaction catalysed by glucose‐6‐phosphate dehydrogenase (G6PDH). During this oxidation, an equimolar amount of NAD is reduced to NADH. The consequent increase in absorbance at 340 nm is directly proportional to the glucose concentration.

The total soluble sugars were estimated by anthrone reagent. An aliquot (1 mL properly diluted) of supernatants was taken in the test tube, mixed with 5 mL of anthrone regent and the mixture was heated in boiling water bath for 10 min followed by cooling. Optical density was read at 620 nm, and the results were calculated using standard curves for sucrose. With the Maltose Assay Kit (Sigma), maltose is converted to two glucose units via a‐D‐Glucosidase. Glucose is further oxidized, resulting in a colorimetric (570 nm) product, proportional to the maltose present. Glucose was measured using the Glucose (HK) Assay Kit (Sigma) according to the manufacturer's instructions.

### Isolation of starch granules

The isolation of starch granules was performed according to Ritte *et al*. (2000a). Banana fruit pulp were sliced, frozen in liquid nitrogen and then grounded in a mortar prior to homogenization. From each tissue, 15–20 g was mixed with 50 mL extraction buffer [100 mm N‐2‐hydroxyethylpiperazine‐N‐2‐ethanesulfonic acid (HEPES)‐KOH (pH 8.0), 1 mm ethylene diamine tetraacetic acid (EDTA), 5 mm dithiothreitol (DTT), 0.5 mm phenylmethanesulfonyl fluoride (PMSF), 0.05% (v/v) Triton‐X‐100] and homogenized for 20 s using a waring blender. The homogenate was passed through a nylon net (100 μm mesh width), and the filtrate was centrifuged for 5 min at 1000 ***g***. Non‐starch materials layered on top of starch pellet were partially removed by rinsing with buffer. The remaining pellet was then suspended in 10–15 mL extraction buffer. Subsequently the crude starch suspension was further purified by centrifugation through a cushion of 95% Percoll (GE) and 5% 0.5 m HEPES‐KON (pH 7.0) as described by Ritte *et al*. (2000b). The pelleted granules were washed twice with extraction buffer, dried under vacuum and stored at −80 °C until use.

### Scanning electron microscopy

The dried starch granules were mounted on stubs by double‐sided tape and then coated with a 10‐nm‐thick platinum layer in the JEOL JFC‐1600 (JEOL, Tokyo, Japan) coating system. The samples were not metalized and examined on a JEOL JSM‐6460LV (JEOL) scanning electron microscope. Scanning electron microscopy was operated in secondary electron mode at 20 kV under ultra‐vacuum conditions. Micrographs at 2000×, 5000× and 10 000× magnification are presented.

### Gene expression analysis

Total RNA was extracted from the pulp of at least five individual fruits using the hot borate method (Wan and Wilkins, 1994). The extracted RNA was used to synthesize cDNA by PrimeScript™ RT reagent Kit with gDNA Eraser (Takara, shiga, Japan). Quantitative real‐time PCR (qRT‐PCR) was carried out on a Bio‐Rad CFX96 Real‐Time PCR System using the GoTaq^®^ qPCR Master Mix Kit (Promega, Madison, WI) following the manufacturer's instructions. The expression levels were normalized to the expression of the reference gene *MaRPS2* (ribosomal protein 2) according to our previous study (Chen *et al*., 2011). Primers are listed in Table S2.

### Extraction of proteins bound to the surface of starch granule

Starch granules were isolated from the samples representative of banana ripening (0 day and 3 days after ethylene treatment). The isolation was performed according to the method described above, except for the volume of extraction buffer which was larger (10‐fold) in the step related to tissue homogenization to isolate the granules, and non‐starch material layered on top of the starch pellet was thoroughly removed by spoon and rinsed with buffer, followed by an additional washing by buffer. The proteins bound to the surface of starch granules were extracted with phenol and purified by ammonium acetate‐methanol precipitation as described by Saravanan and Rose (2004).

### iTRAQ analysis of proteins bound to the starch granule surface

The concentration of proteins bound to the starch granule surface was measured with a Bradford protein assay kit (Bio‐Rad Laboratories (Shanghai) Co., Ltd., Shanghai, China). Each protein sample was reduced, alkylated and subjected to tryptic hydrolysis. The 3‐plex iTRAQ Labeling, Strong cation exchange (SCX) and RP HPLC‐MS/MS were performed by Fitgene Biological Technology Co. Ltd (FITGENE, Guangzhou, China). The peptides were identified by the Peptide Prophet algorithm (Keller *et al*., 2002).

### Preparation of MaGWD1 polyclonal antibody and immunoblotting

The N‐terminal (1‐400 aa) coding sequence of MaGWD1 was cloned into pET28a(+) (Novagen, Darmstadt, Germany) generating a His‐MaGWD1‐N construct and transformed into *Escherichia coli* strain BL21(DE3). The recombinant proteins were induced and affinity purified using His60 Ni Superflow Resin (Clontech, California, United States). His fusion proteins were separated by SDS‐PAGE. Bands of interest were excised and used as antigens for antibody production. Antibodies were produced in rabbit by HuaAn Biotechnology Company (Hangzhou, China). The primers used for vector construction are listed in Table S2.

Dry starch was mixed with protein denaturing buffer (62.5 mm Tris‐HCl (pH 6.8), 2% (w/v) SDS, 10% (w/v) glycerol, 20 mm DTT and 0.005% (w/v) bromophenol blue) by the ratio of 30 μL per mg dry starch, and the suspension was heated for 5 min at 95 °C according to Ritte *et al*. (2000a). After a 2 min of centrifugation at 11 000 ***g***, the supernatant was applied to 7.5% SDS‐PAGE. Western blotting analysis was conducted using anti‐MaGWD1‐N antibody, with secondary goat anti‐rabbit IgG peroxidase antibody (Thermo Scientific, Illinois, United States). Detection was carried out using the chemiluminescent substrate SuperSignal West Pico (Thermo Scientific) for horse‐radish peroxidase and imaged on a ChemiDoc™ MP Imaging System (Bio‐Rad Laboratories).

### Promoter isolation and analysis

Genomic DNA was extracted from banana leaves using the DNeasy Plant Mini Kit (QIAGEN China (Shanghai) Co., Ltd, Shanghai, China). The promoters of starch degradation‐related genes were isolated according to the sequences in banana whole‐genome sequence database (D'Hont *et al*., 2012), using the primers listed in Table S2. The amplification products were cloned into the pGEM‐T Easy vector (Promega (Beijing) Biotech Co., Ltd, Beijing, China) and sequenced. Conserved E‐box motifs of the promoters were predicted using the Plant‐CARE (http://bioinformatics.psb.ugent.be/webtools/plantcare/html) databases.

### Promoter activity assay

For the promoter activity assay, the target promoter region (~1.5 kb) was amplified by PCR using the specific primers listed in Table S2. The PCR product was inserted into the vector pBI121 by replacing the CaMV 35S promoter to generate the construct containing the GUS‐coding region under the control of the promoter of interest. The resulting recombinant construct *pro*::GUS, positive control 35S::GUS and negative control empty::GUS were transfected into banana pulp subjected to 0 (control) or 0.2% (v/v) ethrel (ethylene releaser) treatment, using the agrobacterium‐mediated method according to Singh *et al*. (2011). Transfected bananas were incubated at 23 °C for 60 h. GUS activity was examined histochemically by placing sliced banana pulp in the appropriate staining buffer (50 mm phosphate buffer (pH7.2), 0.5 mm K_4_Fe(CN)_6_.H_2_O, 0.5 mm K_3_Fe(CN)_6_, 0.1% (v/v) Triton X‐100) containing 0.5 mm X‐Gluc and incubated in the dark at 37 °C for 12–24 h. The tissue was decolorized in 70% ethanol for 1 h for proper visualization.

### Yeast one‐hybrid library screening assay

Yeast one‐hybrid library screening assay was performed with the Matchmaker™ Gold Yeast One‐Hybrid System (Clontech). The *MaGWD1* promoter was cloned into pAbAi as the bait (Primers are listed in Table S2). Plasmid was linearized and transformed into Y1H Gold strain to generate a bait‐specific reporter strain. Positive yeast strains were then transformed with pGADT7‐AD, which contained cDNA libraries (prey libraries) of banana fruit. The protein–DNA interaction was determined based on the growth ability of the co‐transformants on SD/‐Leu medium with Aureobasidin A (AbA), according to the manufacturer's protocol. When a prey protein binds to the bait sequence, the GAL4 AD activates expression of AbA^r^ which allows the cells to grow on media containing the AbA antibiotic.

### Dual‐luciferase transient expression assay

The promoters of 26 starch degradation‐related genes, including *MaGWD1*,* MaPWD1*,* MaSEX4*,* MaLSF1*,* MaLSF2*,* MaBAM1‐MaBAM4*,* MaBAM7*,* MaBAM8*,* MaBAM10*,* MaAMY2B*,* MaAMY2C*,* MaAMY3*,* MaAMY3A*,* MaAMY3C*,* MaISA2*,* MaISA3*,* MaPHS2*,* MaMEX1*,* MaMEX2*,* MapGlcT2‐1*,* MapGlcT2‐2*,* MapGlcT4‐1* and *MapGlcT4‐2*, were cloned into pGreenII 0800‐LUC double‐reporter vector, while MabHLH6 was cloned into the pEAQ vector as effector, as described by Hellens *et al*. (2005). All primers used for generating constructs for transient expression assay are listed in Table S2.

The constructed reporter and effector plasmids were transiently expressed in tobacco (*Nicotiana benthamiana*) leaves as described by Hellens *et al*. (2005). The dual‐luciferase assay kit (Promega) was used to analyse the transient expression in tobacco leaves after 2 days of infiltration. Absolute LUC and REN were measured in a Luminoskan Ascent Microplate Luminometer (Thermo Scientific) according to the manufacturer's instructions, with a 5‐s delay and 15‐s integrated measurements. The transcriptional activation activity of MabHLH6 to starch degradation‐related genes’ promoters is indicated by the ratio of LUC to REN. At least six biological repeats were assayed for each combination.

### Protein expression and electrophoretic mobility shift assay (EMSA)

The C‐terminal (276‐478 aa) coding region of *MabHLH6* was PCR amplified and cloned into pGEX‐4T‐1 (GE Healthcare Life Sciences (China), Beijing, China) to fuse in‐frame with the glutathione S‐transferases (GST) tag. The recombinant GST‐MabHLH6‐C construct was transformed into *Escherichia coli* strain BM Rosetta (DE3). Protein expression was induced in a 500‐mL culture using 0.8 mm isopropyl β‐D‐1‐thiogalactopyranoside (IPTG), and cells were collected after 6‐h induction at 30 °C. The recombinant protein was purified using a GST‐Tagged Protein Purification Kit (Clontech) following the instructions. The purified protein was checked for size and purity by SDS‐PAGE and Coomassie Brilliant Blue staining. Protein concentration was determined using a RC/DC Protein Assay Kit, based on the Lowry assay (Bio‐Rad).

The fragments of ~59 bp containing E‐box (CANNTG) in the promoters of starch degradation genes were synthesized (Sangon Biotech, Shanghai, China) and labelled with biotin at the 5′ end by Pierce™ Biotin 3′ End DNA Labeling Kit (Thermo Scientific). The same but unlabelled DNA fragment was used as a competitor, while a version in which the E‐box (CANNTG) within probe changed into mE‐box (ACNNGT), as described previously (Ji *et al*., 2014), was used as a mutant competitor in the assay. EMSA was performed using a LightShift Chemiluminescent EMSA kit (Thermo Scientific) according to the instructions. Biotin‐labelled DNA was detected using the Chemiluminescent Nucleic Acid Detection Module Kit (Thermo Scientific) according to the manufacturer's protocol. The probes used in the EMSA assay are listed in Table S2 and Text S1.

### Preparation of MabHLH6‐specific polyclonal antibody and detection of MabHLH6 protein during banana fruit ripening

The full‐length coding sequence of MabHLH6 was cloned into pET28a(+) (Novagen) generating His‐MabHLH6 construct and transformed into *Escherichia coli* strain BL21(DE3). The recombinant proteins were affinity purified, and antibody was produced as described above. The primers used for vector construction are listed in Table S2.

Total protein from banana fruit pulp was extracted with phenol and purified by ammonium acetate‐methanol precipitation. Separation of proteins was performed by SDS‐PAGE using 30‐μg protein per lane. After electrophoresis, protein preparation was electro‐transferred onto nitrocellulose membrane (0.45 μm, Thermo Scientific) using a Bio‐Rad transfer apparatus. Western blotting analysis was conducted using anti‐MabHLH6 antibody, with secondary goat anti‐rabbit IgG peroxidase antibody (Thermo Scientific). Anti‐GADPH was used as a reference control. Detection was carried out as described above.

### Chromatin immunoprecipitation (ChIP)‐qPCR analysis

Chromatin immunoprecipitation was performed as described by Han *et al*. (2016). Chromatin extracts were prepared from unripe and ripe banana fruit pulp cross‐linked by 1% formaldehyde. The chromatin was sheared to an average length of 500 bp by sonication and immunoprecipitated with the specific anti‐MabHLH6 antibody. ChIP‐qPCR assays were repeated with three biological replicates. The DNA immunoprecipitated by anti‐MabHLH6 antibody was analysed using real‐time qPCR in triplicate. IgG was used as an internal negative control to the ChIP‐qPCR enrichment signal. The primers used for ChIP‐qPCR analyses are listed in Table S2.

## Conflict of interest

The authors declare no conflict of interests.

## Supporting information


**Figure S1** Expression of 38 starch‐degradation‐associated genes in banana fruit pulp with three different ripening treatments.
**Figure S2** Phylogenetic tree of 38 starch‐degradation‐associated genes in banana.
**Figure S3** Phylogenetic tree of MabHLH6.
**Figure S4** Sequence logo of the bHLH domain in MabHLH6.
**Figure S5** Subcellular localization and transcriptional activation of MabHLH6 in tobacco leaves.
**Figure S6** Transcription activation of MabHLH6 in yeast.
**Table S1** Genome IDs and accession numbers of 38 starch‐degradation‐associated genes in this study.
**Table S2** Primers used in this study.
**Data S1** Nucleotide sequences of the promoters of starch degradation enzyme genes.
