# Supplementary material for: A comprehensive investigation of starch degradation process and identification of a transcriptional activator MabHLH6 during banana fruit ripening
Source: Plant Biotechnol J. 2017 Jun 30;16(1):151–64. doi: 10.1111/pbi.12756 (PMC5785343; doi:10.1111/pbi.12756)
Supplement: Supplementary file 1 — Figure S1 Expression of 38 starch‐degradation‐associated genes in banana fruit pulp with three different ripening treatments. Figure S2 Phylogenetic tree of 38 starch‐degradation‐associated genes in banana. Figure S3 Phylogenetic tree of MabHLH6. Figure S4 Sequence logo of the bHLH domain in MabHLH6. Figure S5 Subcellular localization and transcriptional activation of MabHLH6 in tobacco leaves. Figure S6 Transcription activation of MabHLH6 in yeast. Table S1 Genome IDs and accession numbers of 38 starch‐degradation‐associated genes in this study. Table S2 Primers used in this study. Data S1 Nucleotide sequences of the promoters of starch degradation enzyme genes. [file PBI-16-151-s001.pdf]

A comprehensive investigation of starch degradation process and identification of a transcriptional activator MabHLH6 during banana fruit ripening

Yun-yi Xiao, Jian-fei Kuang, Xin-na Qi, Yu-jie Ye, Zhen-Xian Wu, Jian-ye Chen, Wang-jin Lu*

Supplemental Fig. S1 Expression of 38 starch-degradation-associated genes in banana fruit pulp with three different ripening treatments.

Supplemental Fig. S2 Phylogenetic tree of 38 starch-degradation-associated genes in banana.

Supplemental Fig. S3 Phylogenetic tree of MabHLH6.

Supplemental Fig. S4 Sequence logo of the bHLH domain in MabHLH6.

Supplemental Fig. S5 Subcellular localization and transcriptional activation of MabHLH6 in tobacco leaves.

Supplemental Fig. S6 Transcription activation of MabHLH6 in yeast.

Supplemental Table S1 Genome IDs and accession numbers of 38 starch-degradation-associated genes in this study.

Supplemental Table S2 Primers used in this study.

Supplemental Text S1 Nucleotide sequences of the promoters of starch degradation enzyme genes.

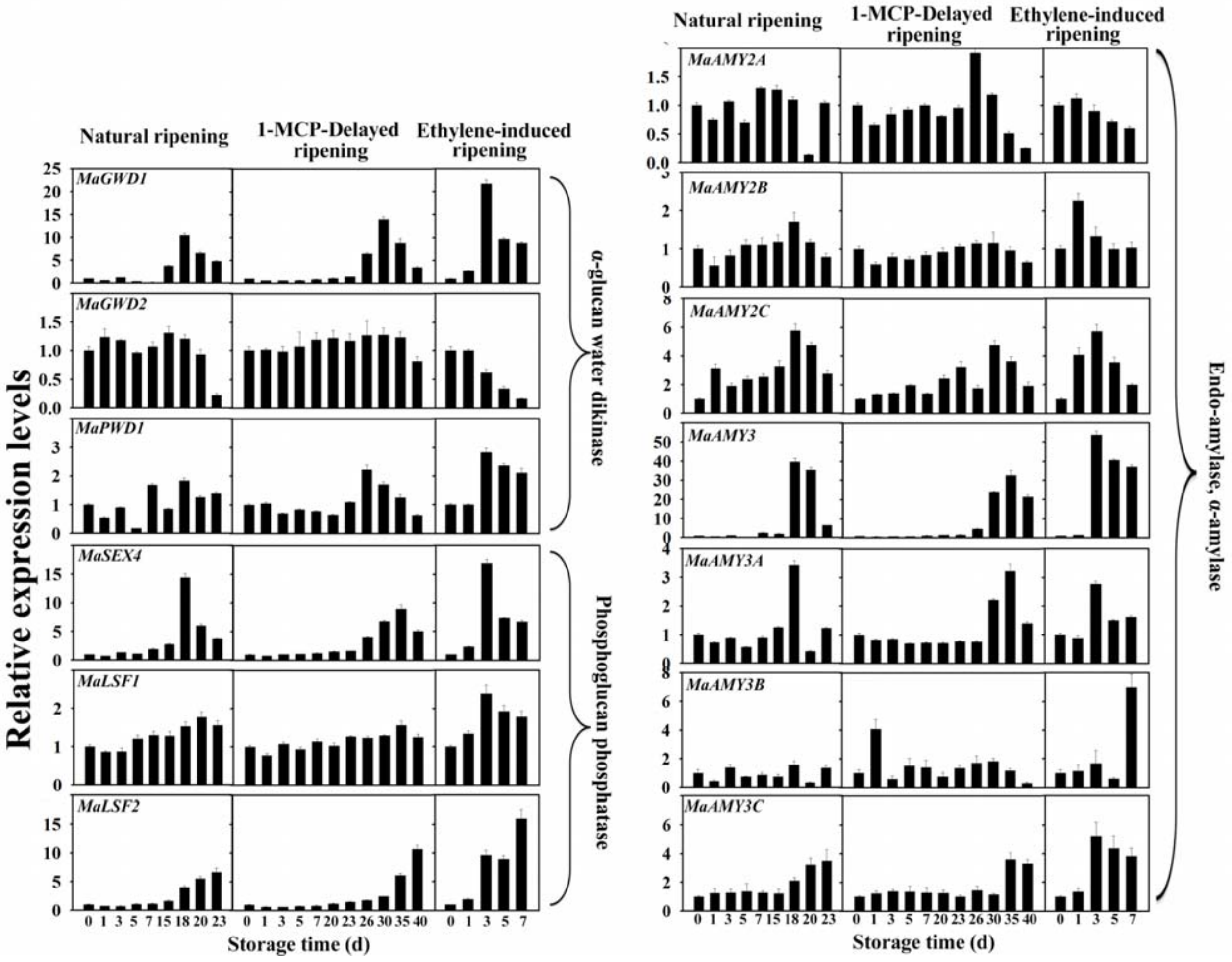

Supplemental Fig. S1 *To be continued*

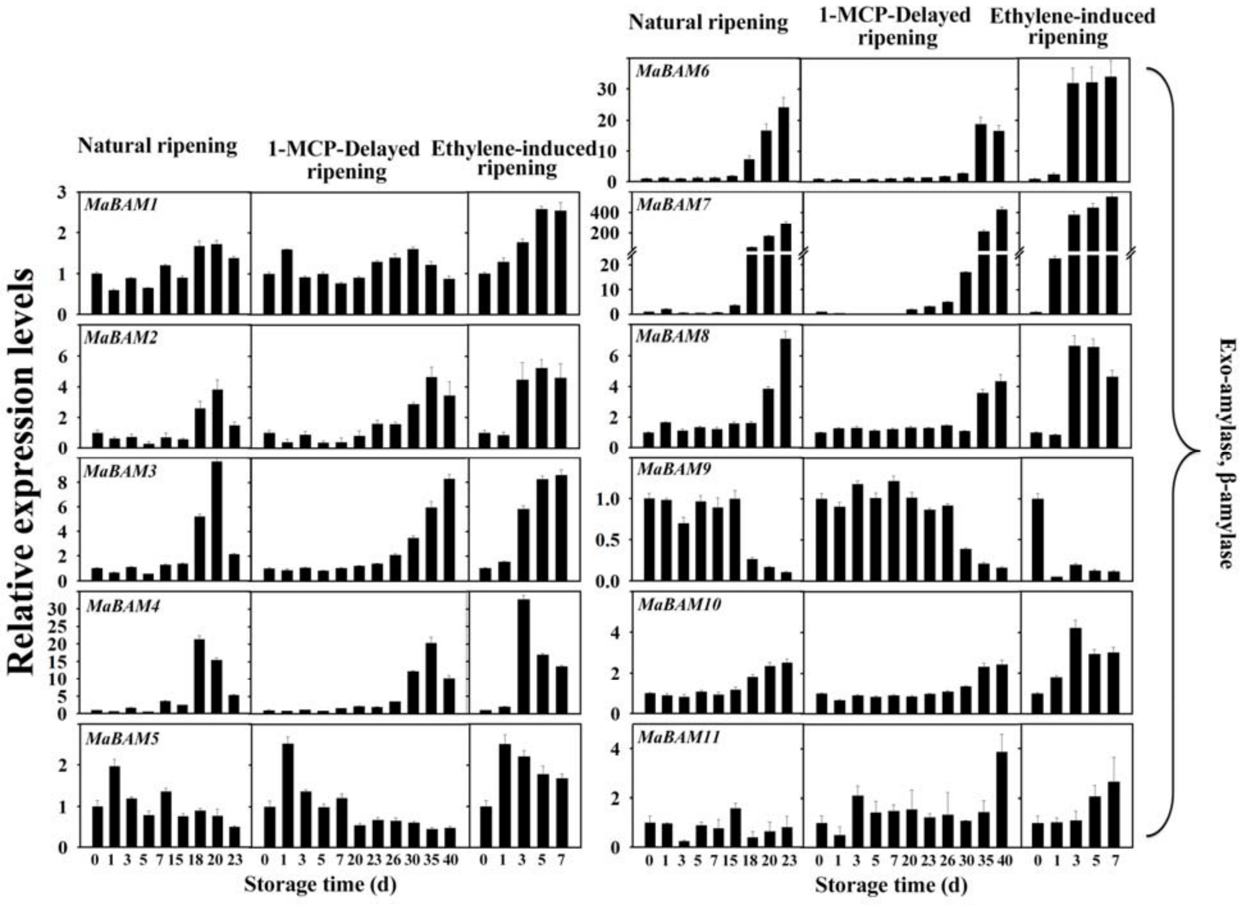

Supplemental Fig. S1 *To be continued*

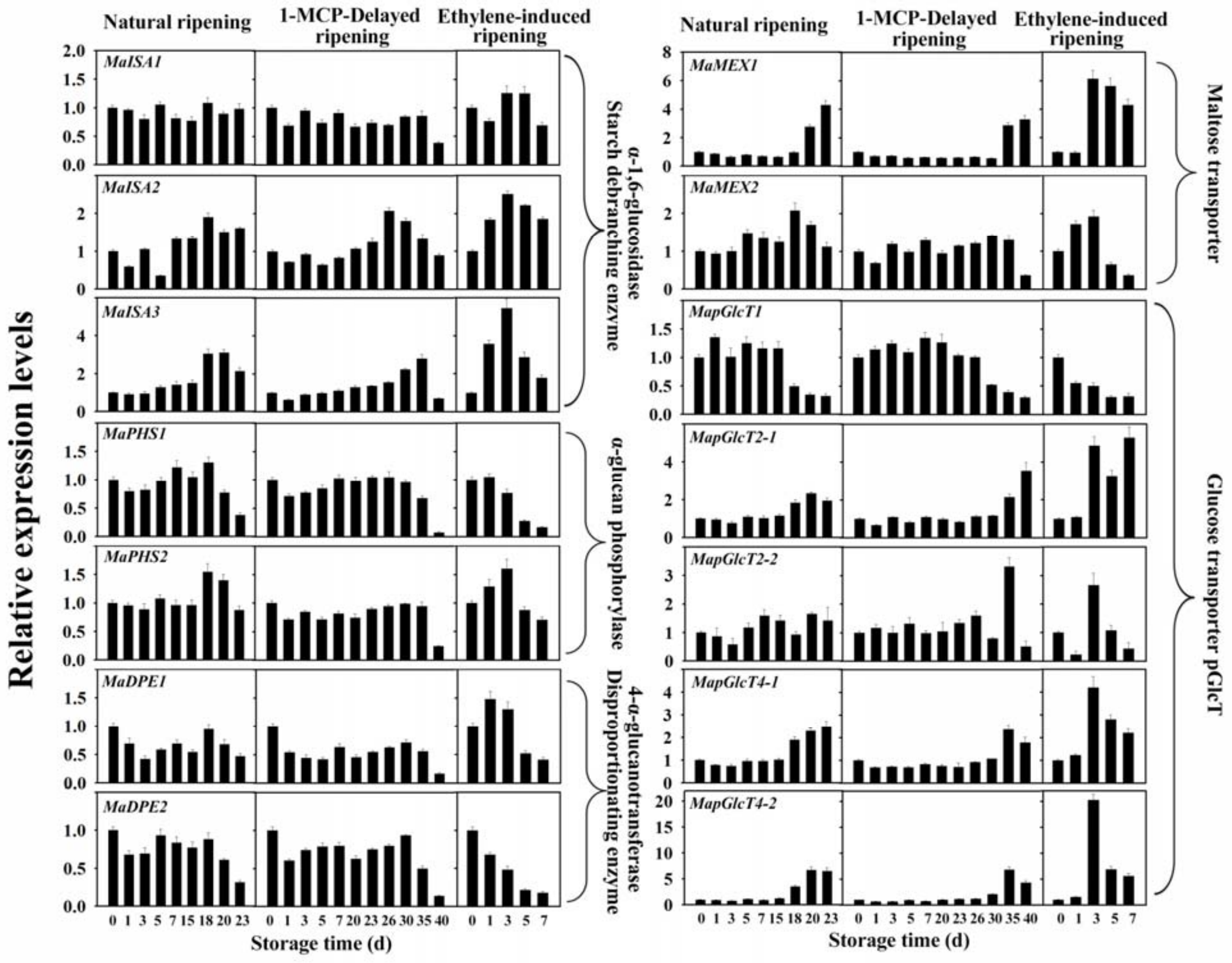

Supplemental Fig. S1 Expression of 38 starch-degradation-associated genes in banana fruit pulp with three different ripening treatments: natural (control), ethylene-induced, and 1-MCP-delayed ripening. The expression levels of each gene are expressed as a ratio relative to the harvest time (0 d of control), which was set as 1. Each value represents the mean ± S.E. of three replicates.

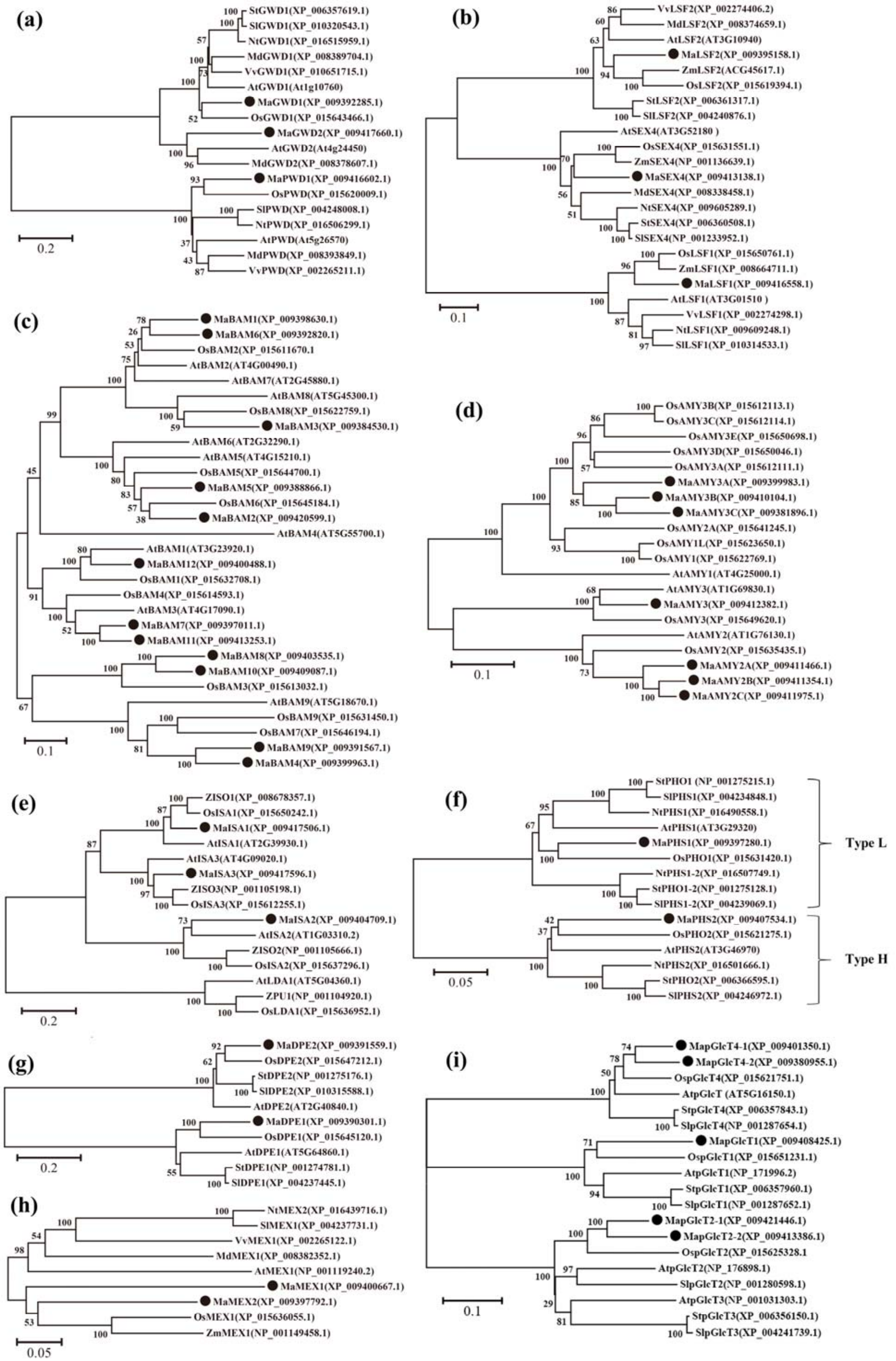
(a)
StGWD1(XP_006357619.1)
SlGWD1(XP_010320543.1)
NtGWD1(XP_016515959.1)
MdGWD1(XP_008389704.1)
VvGWD1(XP_010651715.1)
AtGWD1(At1g10760)
MaGWD1(XP_009392285.1)
OsGWD1(XP_015643466.1)
MaGWD2(XP_009417660.1)
AtGWD2(At4g24450)
MdGWD2(XP_008378607.1)
MaPWD1(XP_009416602.1)
OsPWD(XP_015620009.1)
SlPWD(XP_004248008.1)
NtPWD(XP_016506299.1)
AtPWD(At5g26570)
MdPWD(XP_008393849.1)
VvPWD(XP_002265211.1)
0.2
(b)
VvLSF2(XP_002274406.2)
MdLSF2(XP_008374659.1)
AtLSF2(AT3G10940)
MaLSF2(XP_009395158.1)
ZmLSF2(ACG45617.1)
OsLSF2(XP_015619394.1)
StLSF2(XP_006361317.1)
SlLSF2(XP_004240876.1)
AtSEX4(AT3G52180)
OsSEX4(XP_015631551.1)
ZmSEX4(NP_001136639.1)
MaSEX4(XP_009413138.1)
MdSEX4(XP_008338458.1)
NtSEX4(XP_009605289.1)
StSEX4(XP_006360508.1)
SlSEX4(NP_001233952.1)
OsLSF1(XP_015650761.1)
ZmLSF1(XP_008664711.1)
MaLSF1(XP_009416558.1)
AtLSF1(AT3G01510)
VvLSF1(XP_002274298.1)
NtLSF1(XP_009609248.1)
SlLSF1(XP_010314533.1)
0.1
(c)
MaBAM1(XP_009398630.1)
MaBAM6(XP_009392820.1)
OsBAM2(XP_015611670.1
AtBAM2(AT4G00490.1)
AtBAM7(AT2G45880.1)
AtBAM8(AT5G45300.1)
OsBAM8(XP_015622759.1)
MaBAM3(XP_009384530.1)
AtBAM6(AT2G32290.1)
AtBAM5(AT4G15210.1)
OsBAM5(XP_015644700.1)
MaBAM5(XP_009388866.1)
OsBAM6(XP_015645184.1)
MaBAM2(XP_009420599.1)
AtBAM4(AT5G55700.1)
AtBAM1(AT3G23920.1)
MaBAM12(XP_009400488.1)
OsBAM1(XP_015632708.1)
OsBAM4(XP_015614593.1)
AtBAM3(AT4G17090.1)
MaBAM7(XP_009397011.1)
MaBAM11(XP_009413253.1)
MaBAM8(XP_009403535.1)
MaBAM10(XP_009409087.1)
OsBAM3(XP_015613032.1)
AtBAM9(AT5G18670.1)
OsBAM9(XP_015631450.1)
OsBAM7(XP_015646194.1)
MaBAM9(XP_009391567.1)
MaBAM4(XP_009399963.1)
0.1
(d)
OsAMY3B(XP_015612113.1)
OsAMY3C(XP_015612114.1)
OsAMY3E(XP_015650698.1)
OsAMY3D(XP_015650046.1)
OsAMY3A(XP_015612111.1)
MaAMY3A(XP_009399983.1)
MaAMY3B(XP_009410104.1)
MaAMY3C(XP_009381896.1)
OsAMY2A(XP_015641245.1)
OsAMY1L(XP_015623650.1)
OsAMY1(XP_015622769.1)
AtAMY1(AT4G25000.1)
AtAMY3(AT1G69830.1)
MaAMY3(XP_009412382.1)
OsAMY3(XP_015649620.1)
AtAMY2(AT1G76130.1)
OsAMY2(XP_015635435.1)
MaAMY2A(XP_009411466.1)
MaAMY2B(XP_009411354.1)
MaAMY2C(XP_009411975.1)
0.1
(e)
ZISO1(XP_008678357.1)
OsISA1(XP_015650242.1)
MaISA1(XP_009417506.1)
AtISA1(AT2G39930.1)
AtISA3(AT4G09020.1)
MaISA3(XP_009417596.1)
ZISO3(NP_001105198.1)
OsISA3(XP_015612255.1)
MaISA2(XP_009404709.1)
AtISA2(AT1G03310.2)
ZISO2(NP_001105666.1)
OsISA2(XP_015637296.1)
AtLDA1(AT5G04360.1)
ZPU1(NP_001104920.1)
OsLDA1(XP_015636952.1)
0.2
(f)
StPHO1 (NP_001275215.1)
SlPHS1(XP_004234848.1)
NtPHS1(XP_016490558.1)
AtPHS1(AT3G29320)
MaPHS1(XP_009397280.1)
OsPHO1(XP_015631420.1)
NtPHS1-2(XP_016507749.1)
StPHO1-2(NP_001275128.1)
SlPHS1-2(XP_004239069.1)
Type L
MaPHS2(XP_009407534.1)
OsPHO2(XP_015621275.1)
AtPHS2(AT3G46970)
NtPHS2(XP_016501666.1)
StPHO2(XP_006366595.1)
SlPHS2(XP_004246972.1)
Type H
0.05
(g)
MaDPE2(XP_009391559.1)
OsDPE2(XP_015647212.1)
StDPE2(NP_001275176.1)
SlDPE2(XP_010315588.1)
AtDPE2(AT2G40840.1)
MaDPE1(XP_009390301.1)
OsDPE1(XP_015645120.1)
AtDPE1(AT5G64860.1)
StDPE1(NP_001274781.1)
SlDPE1(XP_004237445.1)
0.2
(h)
NtMEX2(XP_016439716.1)
SlMEX1(XP_004237731.1)
VvMEX1(XP_002265122.1)
MdMEX1(XP_008382352.1)
AtMEX1(NP_001119240.2)
MaMEX1(XP_009400667.1)
MaMEX2(XP_009397792.1)
OsMEX1(XP_015636055.1)
ZmMEX1(NP_001149458.1)
0.05
(i)
MapGlcT4-1(XP_009401350.1)
MapGlcT4-2(XP_009380955.1)
OspGlcT4(XP_015621751.1)
AtpGlcT (AT5G16150.1)
StpGlcT4(XP_006357843.1)
SlpGlcT4(NP_001287654.1)
MapGlcT1(XP_009408425.1)
OspGlcT1(XP_015651231.1)
AtpGlcT1(NP_171996.2)
StpGlcT1(XP_006357960.1)
SlpGlcT1(NP_001287652.1)
MapGlcT2-1(XP_009421446.1)
MapGlcT2-2(XP_009413386.1)
OspGlcT2(XP_015625328.1
AtpGlcT2(NP_176898.1)
SlpGlcT2(NP_001280598.1)
AtpGlcT3(NP_001031303.1)
StpGlcT3(XP_006356150.1)
SlpGlcT3(XP_004241739.1)
0.1

Supplemental Fig. S2 Phylogenetic tree of 38 starch-degradation-associated genes in banana. (a) Phylogenetic tree of α-glucan water dikinase and phosphoglucan water dikinase; (b) Phylogenetic tree of phosphoglucan phosphatase; (c) Phylogenetic tree of β-amylase; (d) Phylogenetic tree of α-amylase; (e) Phylogenetic tree of starch debranching enzyme (ISA); (f) Phylogenetic tree of α-glucan phosphorylase; (g) Phylogenetic tree of disproportionating enzyme (DPE); (h) Phylogenetic tree of maltose excess protein; (i) Phylogenetic tree of plastidic glucose transporter.

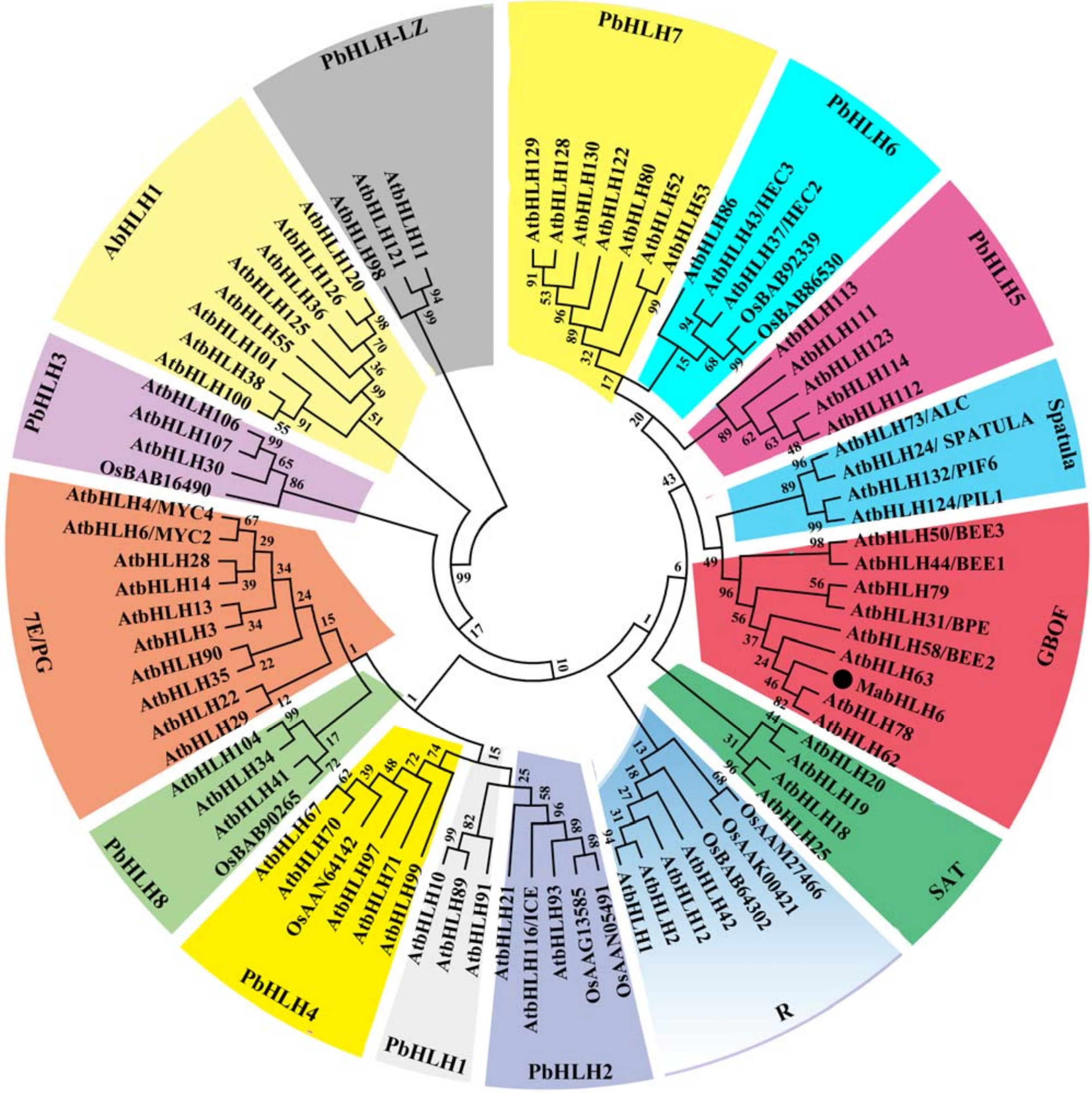

Supplemental Fig. S3 Phylogenetic tree of MabHLH6. Banana MabHLH6 (black circles) were aligned with those of the *Arabidopsis* and rice bHLH families as designated by Peng *et al*. (2013). Multiple alignment was carried using CLUSTALW and the phylogenetic tree was constructed with MEGA6.0 using a bootstrap test of phylogeny with Neighbor-Joining test and default parameters.

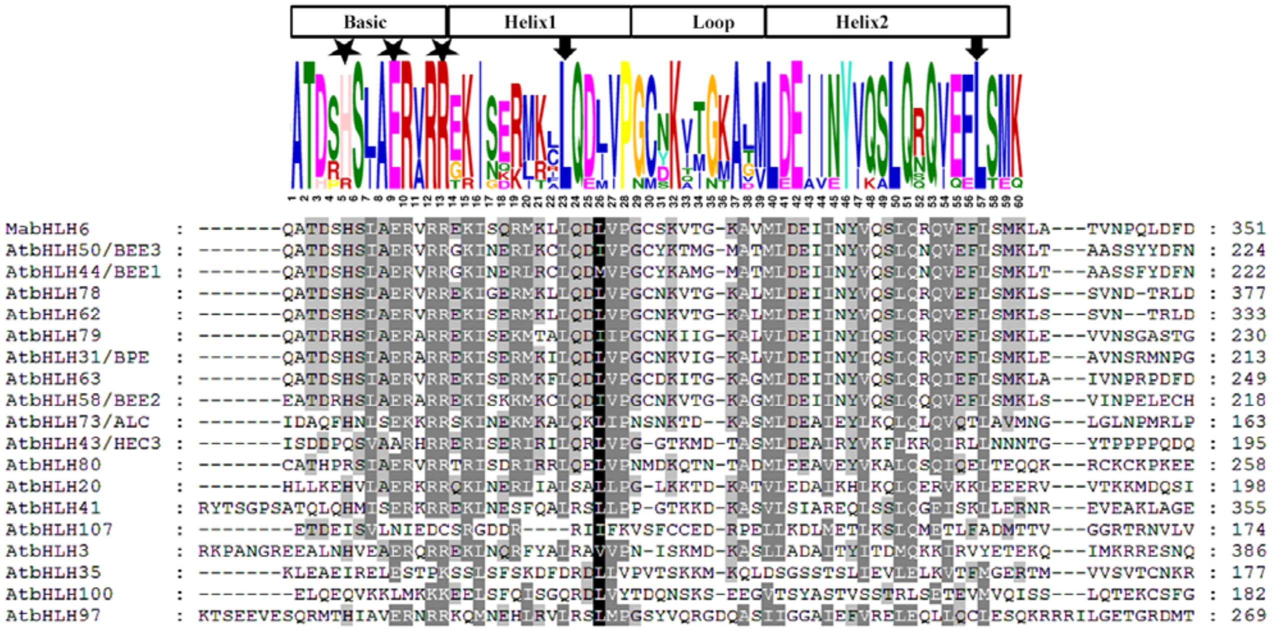

Supplemental Fig. S4 Sequence logo of the bHLH domain in MabHLH6. MabHLH6 were aligned with *Arabidopsis* bHLH proteins. Identical and similar amino acids were presented by black and gray shading, respectively. Gaps were introduced to optimize alignment. Logo of the bHLH domain was found by MEME (http://meme-suite.org/tools/meme). The H5, E9 and R13 amino acids in the basic domain that are important for DNA binding are indicated by stars. Amino acids important for dimerization of the helix-loop-helix domain are indicated by arrows.

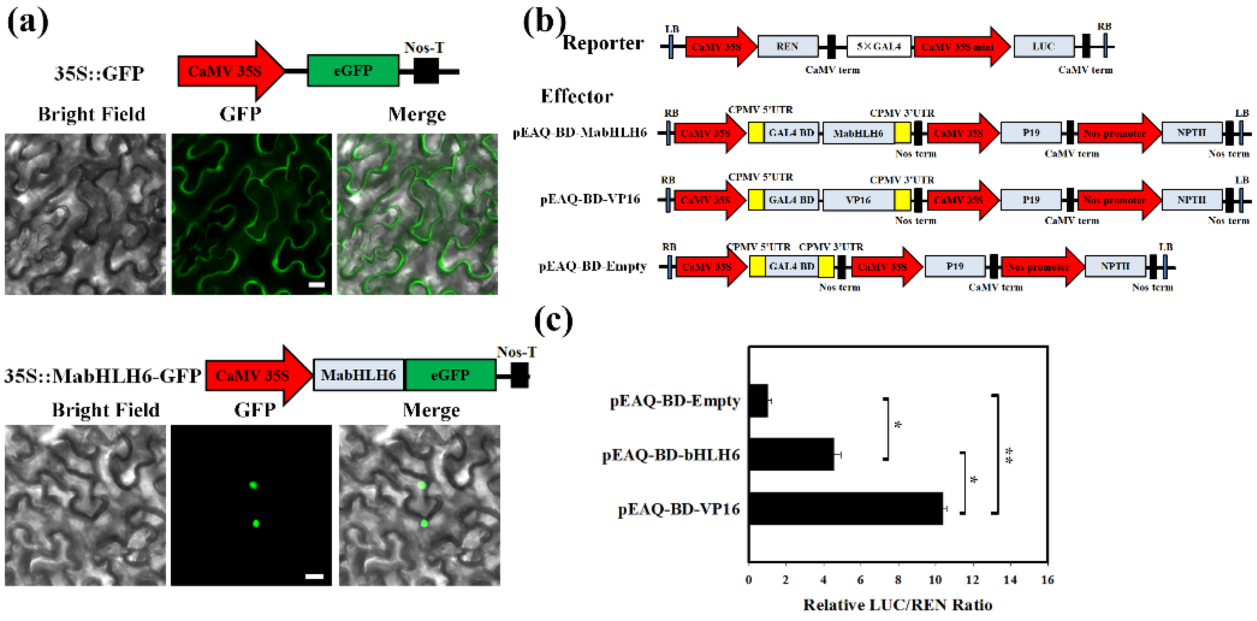

Supplemental Fig. S5 Subcellular localization and transcriptional activation of MabHLH6 in tobacco leaves. (a) The fusion protein (CaMV35S::MabHLH6-GPF) and control (CaMV35S::GFP) were transiently expressed in *Nicotiana benthamiana* leaves respectively by *Agrobacterium tumefaciens* strain EHA105. GFP fluorescence was observed with a fluorescence microscope. Images were taken in a dark field for green fluorescence, while the outline of the cell and the merged were photographed in a bright field. Bars, 20 μm. (b) Plasmid combinations of LUC reporter driven by the mini-35S plus five GAL4 binding elements, and effectors expressing MabHLH6 fused to GAL4. (c) Transcriptional activation activity of MabHLH6. The transactivation ability of MabHLH6 is indicated by the ratio of LUC to REN. Each value represents the means of six biological replicates, and vertical bars represent the S.E. The asterisk indicates a significant difference at the 5% level compared to the negative control pEAQ-BD-Empty. The ratio of LUC/REN of the pEAQ-BD-Empty vector was used as calibrator (set as 1).

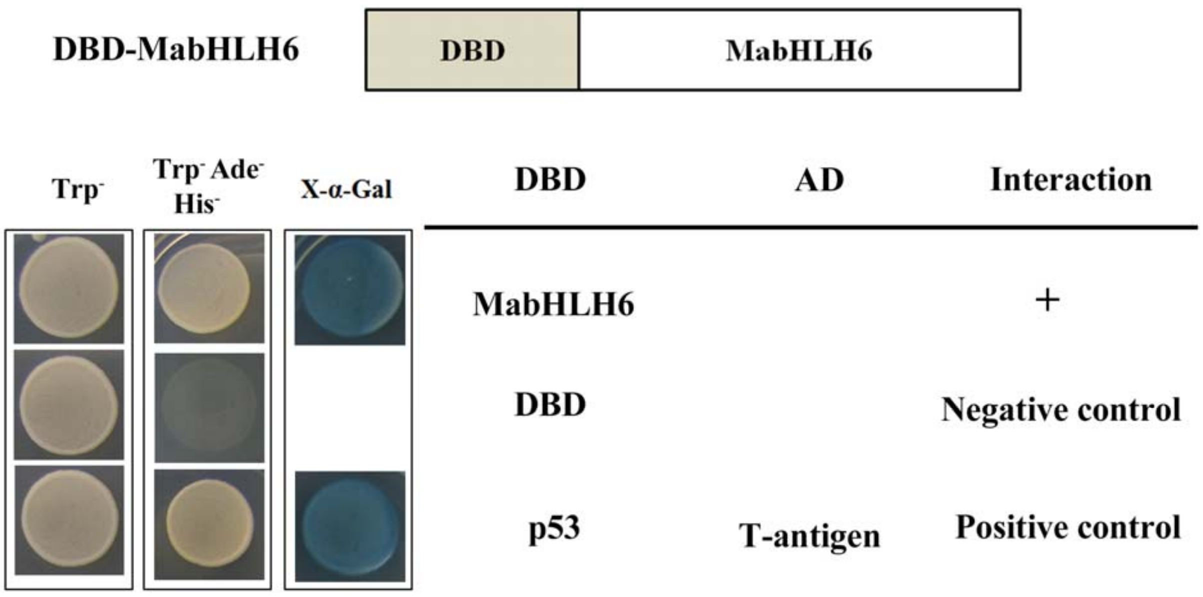

Supplemental Fig. S6 Transcription activation of MabHLH6 in yeast two-hybrid system. MabHLH6 fused with the GAL4 DNA-binding domain (DBD-MabHLH6), together with the positive control (p-53+T-antigen) and negative control (pGBKT7) were transformed into yeast strain Y2H-gold. Yeast clones transformed with different constructs were grown on SD plates without tryptophan or without tryptophan, histidine, and adenine but containing 125 μM Aureobasidin A for 3 d at 30 °C. Transcription activation was monitored by the detection of yeast growth and an α-galactosidase (α-Gal) assay.

Supplemental Table S1 Genome IDs and accession numbers of 38 starch-degradation-associated genes in this study.

Protein abbreviation	Locus (NCBI, October 2016)	Locus (Genome, January 2016)	Locus (Genome, July 2012)	Publication
**α-glucan water dikinase (GWD), phosphoglucan water dikinase (PWD)**				
MaGWD1	XP_009392285.1	Ma03_p15660.1)	GSMUA_Achr3G14470_001	
MaGWD2	XP_009417660.1	Ma09_p15070.1	GSMUA_Achr9T15680_001	
MaPWD	XP_009416602.1	Ma09_p07100.1	GSMUA_Achr9G07400_001	
**Phosphoglucan phosphatase**				
Starch excess 4 (MaSEX4)	XP_009413138.1	Ma08_p13120.1	GSMUA_Achr8T14150_001	
Like Sex Four 1 (MaLSF1)	XP_009416558.1	Ma09_p06790.1	GSMUA_Achr9T07030_001	
Like Sex Four 2 (MaLSF2)	XP_009395158.1	Ma04_p00190.1	GSMUA_Achr4T00220_001	
**Exo-amylase, β-amylase (BAM)**				
MaBAM1	XP_009398630.1	a01_p10710.2	GSMUA_Achr1G17440_001	MaBAM1 (Jourda *et al*., 2016)
MaBAM2	XP_009420599.1	Ma10_p10330.1	GSMUA_Achr10G09320_001	MaBAM12 (Jourda et al., 2016)
MaBAM3	XP_009384530.1	Ma02_p06550.1	GSMUA_Achr2G04590_001	MaBAM2 (Jourda et al., 2016)
MaBAM4	XP_009399963.1	Ma05_p07800.1	GSMUA_Achr5G08040_001	Ma-bmy(Do Nascimento et al., 2006)\MaBAM7 (Jourda et al., 2016)
MaBAM5	XP_009388866.1	Ma02_p15910.1	GSMUA_Achr2G14060_00	MaBAM3 (Jourda et al., 2016)\(Gao et al., 2016)
MaBAM6	XP_009392820.1	Ma03_p28390.1	GSMUA_Achr3G27310_001	MaBAM5 (Jourda et al., 2016)\(Gao et al., 2016)
MaBAM7	XP_009397011.1	Ma04_p18390.1	GSMUA_Achr4G17840_001	MaBAM6 (Jourda et al., 2016)\(Gao et al., 2016)
MaBAM8	XP_009403535.1	Ma06_p07470.1	GSMUA_Achr6G05840_001	MaBAM9 (Jourda et al., 2016)
MaBAM9	XP_009391567.1	Ma03_p08740.1	GSMUA_Achr3G08860_001	MaBAM4 (Jourda et al., 2016)
MaBAM10	XP_009409087.1	Ma07_p10880.1	GSMUA_Achr7G11100_001	MaBAM10 (Jourda et al., 2016)
MaBAM11	XP_009413253.1	Ma08_p15460.1	GSMUA_Achr8G14930_001	MaBAM11 (Jourda et al., 2016)
**Endo-amylase, α-amylase (AMY)**				
MaAMY2A	XP_009411466.1	Ma04_p21990.1	GSMUA_Achr1G02190_001	MaAMY1 (Jourda et al., 2016)
MaAMY2B	XP_009411354.1	Ma04_p22000.1	GSMUA_Achr1G02210_001	MaAMY2 (Jourda et al., 2016)
MaAMY2C	XP_009411975.1	Ma04_p22020.1	GSMUA_Achr1G02230_001	MaAMY3 (Jourda et al., 2016)
MaAMY3	XP_009412382.1	Ma08_p04100.1	GSMUA_Achr8G04140_001	MaAMY12 (Jourda et al., 2016)\(Gao et al., 2016)
MaAMY3A	XP_009399983.1	Ma05_p08000.1	GSMUA_Achr7G26940_001	MAmy(Junior et al., 2006) \MaAMY11 (Jourda et al., 2016)\(Gao et al., 2016)
MaAMY3B	XP_009410104.1	Ma07_p20300.1	GSMUA_Achr7G18920_001	MaAMY10 (Jourda et al., 2016)\(Gao et al., 2016)
MaAMY3C	XP_009381896.1	Ma10_p30040.1	GSMUA_Achr10G30130_001	MaAMY13 (Jourda et al., 2016)
**α-1,6-glucosidase Starch debranching enzyme (DBE)**				
Isoamylase 1 (MaISA1)	XP_009417506.1	Ma09_p13520.2	GSMUA_Achr9G14010_001	MaDBE3 (Jourda et al., 2016)
Isoamylase 2 (MaISA2)	XP_009404709.1	Ma06_p17710.1	GSMUA_Achr6G16200_001	MaDBE2 (Jourda et al., 2016)
Isoamylase 3 (MaISA3)	XP_009417596.1	Ma09_p14380.2	GSMUA_Achr9G14980_001	Maisa (Bierhals et al., 2004)\MaDBE4 (Jourda et al., 2016)
**α-glucan phosphorylase (PHS)**				
MaPHS1	XP_009397280.1	Ma04_p39130.1	GSMUA_Achr4G32600_001	(Gao et al., 2016)\Pho1 (Mainardi et al., 2006)
MaPHS2	XP_009407534.1	Ma06_p35790.1	GSMUA_Achr6G33840_001	Pho2 (Mainardi et al., 2006)
**4-α-glucanotransferase Disproportionating enzyme (DPE)**				
MaDPE1	XP_009390301.1	Ma02_p23910.1	GSMUA_Achr2G22040_001	
MaDPE2	XP_009391559.1	Ma03_p08680.1	GSMUA_Achr3G08780_001	
**Maltose transporter, Maltose Excess Protein**				
MaMEX1	XP_009400667.1	Ma01_p08940.1	GSMUA_Achr1G15560_001	
MaMEX2	XP_009397793.1	Ma04_p34170.1	GSMUA_Achr4G27620_001	
**Glc transporter, plastidic Glucose Transporter**				
MapGlcT1	XP_009408425.1	Ma07_p02140.1	GSMUA_Achr7G02180_001	
MapGlcT2-1	XP_009421446.1	Ma10_p15040.1	GSMUA_Achr10G14930_001	
MapGlcT2-2	XP_009413386.1	Ma08_p19450.1	GSMUA_Achr8G15970_001	
MapGlcT4-1	XP_009401350.1	Ma05_p26070.1	GSMUA_Achr5G23720_001	
MapGlcT4-2	XP_009380955.1	Ma10_p25440.1	GSMUA_Achr10G25480_001	

Supplemental Table S2 Summary of primers used in this study

Assay	Primer sequence (5'-3')		Restriction Site
Full length cloning	MabHLH6-F: ATGAACTCTGGGTCGTCGG		
	MabHLH6-R: TCAAAGCTCAATTTTCATTTGAG		
Subcellular localization	MabHLH6-GFP-F: gactctagagcagtcgacgATGAACTCTGGGTCGTC		Sal I
	MabHLH6-GFP-R: cgggatccaccggtcgccAAGCTCAATTTTCATTTGAG		BamH I
RT-qPCR	MabHLH6-qF: TCATCCAGTCTCTTACGGCG	MabHLH6-qR: GGTTTCGGTGAAGCAGCATC	
	MaGWD1-qF: AGACTTCCCACAACATAGAG	MaGWD1-qR: AAGTGCCTGACAGATTACGA	
	MaPWD1-qF: CAATAAGGCTGATGGGGATGA	MaPWD1-qR: AATGTCACTTTCTCCTGTCGG	
	MaSEX4-qF: GAAGAACTTACCTGAAGGACGC	MaSEX4-qR: CGACACTTGTATCGCTACTGGA	
	MaLSF1-qF: CAGACGAATGCGGGAATGTT	MaLSF1-qR: AACGGCTGGCTGGAAGATAA	
	MaLSF2-qF: AATGGGCTATTTCTGAGGGTAA	MaLSF2-qF: GCTCTTTCCACGGGTCATTCTT	
	MaBAM1-qF: CACAGATTCAGAAGGAAGATGC	MaBAM1-qR: TCACAGGATAAGATGGGTAACG	
	MaBAM2-qF: CAGGCATTGGAGAGTTTCAG	MaBAM2-qR: GTCGGCTTGTCGTTGTATTC	
	MaBAM3-qF: TGCTGCGGACCACAGGCTT	MaBAM3-qR: GCTTTCCCAGGCGGTGTTCA	
	MaBAM4-qF: GCAAGAGGCATGGGGTGAAG	MaBAM4-qR: GCTCCTGATGAACTCGGTAAAC	
	MaBAM5-qF: GCAACCCGAAGAAGCCAAGA	MaBAM5-qR: GCCCAGTTTTCGCTCTCCAG	
	MaBAM6-qF: GCTCTTTCCACGGGTCATTCTT	MaBAM6-qR: CTGGATAAGATGGGTAGCG	
	MaBAM7-qF: GCCGACGACAGCATTGACCT	MaBAM7-qR: CAGCCATCTTCGAGTTCTTG	
	MaBAM8-qF: GATGGATCTTGGAACACACC	MaBAM8-qR: CGAACATGCGGAGTATTGGA	
	MaBAM9-qF: GTGGGTACTACTGGCTTCCG	MaBAM9-qR: TGCTCCTGATGAACTCTGTG	
	MaBAM10-qF: GCCCTATTGGTGCGAGTAAC	MaBAM10-qR: GCTGTAGTGCCAGTGTATGC	
	MaBAM11-qF: GGAACATGGAACACCGATTA	MaBAM11-qR: CCTGATAATTTAGCACCCGTG	
	MaAMY2A-qF: GGTGGTGGTCTTCACGAGCA	MaAMY2A-qR: TGGTATTCCTGGATGAGTGAG	
	MaAMY2B-qF: CACTGGGTCAACTCAGGGTC	MaAMY2B-qR: CGATTTTTGCGGCATAGAGAT	
	MaAMY2C-qF: CAGATACGCAGTTTGGCACA	MaAMY2C-qR: TTAGAAGACAGAAAGCAAGACG	
	MaAMY3-qF: AGGAACAGGCTCTGGGTATG	MaAMY3-qR: AGACTCAGTGGGTGGTGGTA	
	MaAMY3A-qF: CAGTTGGCTAAAACCAGAACAC	MaAMY3A-qR: GGAAGTTGGAAGGAACGAGATT	
	MaAMY3B-qF: CGCCATTAGGACTCGCAACG	MaAMY3B-qR: GATCAGCATCAGCAACTAGA	
	MaAMY3C-qF: CGAGACTGACATCGGCTTC	MaAMY3C-qR: GGTCGTAGGCAGGCTTTCCA	
	MaISA1-qF: ATGGTTTCCCAAGGAGTGCC	MaISA1-qR: CGACAGAAACGGTAGAAATCAG	
	MaISA2-qF: GCTGGAACTGTGGCGACGAA	MaISA2-qR: GTGGAGTAGCCGCACTCATC	
	MaISA3-qF: CTCTACCAGGTGAACAAGCG	MaISA3-qR: TGCCGTGAGCGAAGACCTAT	
	MaPHS1-qF: AAAGCCTGTCGTCTTGCCAT	MaPHS1-qR: GCGGGCTACAGAATCACCTTT	
	MaPHS2-qF: GTCCCAAGGCTGCGTAAAG	MaPHS2-qR: CCAGTAGAGGGTTGTAGTCA	
	MaDPE1-qF: CAACCCGCACTTGCCTCATA	MaDPE1-qR: TCCTCCTCCCCCAAGTTTCCC	
	MaDPE2-qF: AGCATACTTCATCATTCAGCA	MaDPE2-qR: CATCAAGCATCAGTGAGTCTA	
	MaMEX1-qF: GCTTATGGATACAGTTCACCC	MaMEX1-qR: CTTGGTAAACTGGCAGAGCA	
	MaMEX2-qF: TGGATGGACTGCCACCCTG	MaMEX2-qR: CCCTGCTGATCGTATTGAAAC	
	MapGlcT1-qF: GAAGCTACCTGGGAATGGC	MapGlcT1-qR: CAGCACCTAAGGCAAACG	
	MapGlcT2-1-qF: GAGGGGACGATAGTGAAAGC	MapGlcT2-1-qR: ACCTGAAGACCCATTGATGC	
	MapGlcT2-2-qF: ATAAGCGTGGAAGAGTTGC	MapGlcT2-2-qR: CTGAAGTGGCGACCATAGA	
	MapGlcT4-1-qF: AGTGGTTGGCACAGTCCTAT	MapGlcT4-1-qR: CACCACGCTCAAGAAGTAGA	
	MapGlcT4-2-qF: TGTTGCTGCTCTCCTTGTGT	MapGlcT4-2-qR: GTGGCGATTCCGAACCTATT	
Promoter isolation	MaGWD1-pro-F: TCCACCGCCACCCCGTTATC	MaGWD1- pro-R: GTGTCCAACAGTATTGCTCATG	
	MaPWD1-pro-F: CTACTGGCTCTTTGGGTAAAGT	MaPWD1-pro-R: TCGGCGGTGGAAGTGAAGAC	
	MaSEX4-pro-F: GATCAGATGTTATGGGAGGTC	MaSEX4-pro-R: GATGTGGTCGTATCACCCCCT	
	MaLSF1-pro-F: GCGTGGGGTCTAATGCAGTC	MaLSF1-pro-R: TGGTGTGGGTCGGAGCTCGT	
	MaLSF2-pro-F: CAGAACCCTGTGCTGATGGC	MaLSF2-pro-R: GCGATTATTACGGGAGCCCTG	
	MaBAM1-pro-F: TGCCCACCACCCTTACGAG	MaBAM1-pro-R: GGCAGGGCGAGGAAGTACC	
	MaBAM2-pro-F: CGGTAGCGACCTGGTTTGG	MaBAM2-pro-R: ACTTACTGGGAGCATCACGA	
	MaBAM3-pro-F: GCTGTTGTTCAGTCTCTCATAG	MaBAM3-pro-R: GCCATCGGGCTCGACGAT	
	MaBAM4-pro-F: TCAGCCTCTGAGTTCTCCAC	MaBAM4-pro-R: CCATCAATCCTGCCTCCATC	

	MaBAM7-pro-F: CAAGATTTATGGGAAGTGACG	MaBAM7-pro-R: AGCTGCAGCCAAAACGTGAAAG	
	MaBAM8-pro-F: CTTGTTACACGACCACCAGC	MaBAM8-pro-R:GAAGACTTGGAGGAGTAGAGG	
	MaBAM10-pro-F: GGGTGATCTGACTTTGGAAC	MaBAM10-pro-R:CGCCCTCATCGCCTTCTTGC	
	MaAMY2B-pro-F: CGTTTCCATGAGTGCCCGGTG	MaAMY2B-pro-R: AACTGATAACTAGATGATTATCAG	
	MaAMY2C-pro-F: CATCTAAACGATCCATCGCG	MaAMY2C-pro-R: AACTGATAACTAGATGATTATCAG	
	MaAMY3-pro-F: TGGGTTTGCTTCCGCTGGCC	MaAMY3-pro-R: CGGCGAACGGTGGGGGAGAA	
	MaAMY3A-pro-F: GAATTGGCATCTATCAGTGCC	MaAMY3A-pro-R: AGAGTATCTGGGACTGAGCC	
	MaAMY3C-pro-F: CGTTGACTGAGTTGTGCTACC	MaAMY3C-pro-R: GAGGATGAGGAGGACGAAGG	
	MaISA2-pro-F: CAAGCATGGAACCAAAGAACT	MaISA2-pro-R: CAGAAAAGTTCACACCTACGGC	
	MaISA3-pro-F: GAGGCATGGATCCGAGGCTT	MaISA3-pro-R: TTGAGAGATCGAAGGAAACA	
	MaPHS2-pro-F: CGTCATCAAGTTGGCCTTGT	MaPHS2-pro-R: GATGGGCGATAGCGGGAACC	
	MaMEX1-pro-F: AGAGGTCAAGGTGGTGCT	MaMEX1-pro-R: AGAGGCGAGAAGGTGGAAGC	
	MaMEX2-pro-F: GCGTGGAGTTGATATGGGTG	MaMEX2-pro-R: CAAGTTGAGACGAAGGAGGG	
	MapGlcT2-1-pro-F:CAAGATATCCTTAGTACCATGCAG	MapGlcT2-1-pro-R: TCATCGCCTATCGGCTTCAG	
	MapGlcT2-2-pro-F: CGGTTTATCTCCATGATTCGG	MapGlcT2-2-pro-R: AACTAAGGCCGGGCCGCATC	
	MapGlcT4-1-pro-F: TCGCCTTCGTGTGGGGTTGA	MapGlcT4-1-pro-R: GGTACACCTCCTTTGGCAGC	
	MapGlcT4-2-pro-F: GGATACAGGAGTCACCATAC	MapGlcT4-2-pro-R: GGGCCATCTCCACCCCGAAA	
	MabHLH6-pro-F: GGTGAGAAGTTGGTGGTGGAGT	MabHLH6-pro-R: CTCCGCGAGGTCGGCAATGC	
**Promoter activity assay**	MaGWD1-pro-GUS-F: tgattacgccaagcttCTTTGGGTTGTGGGTCCGCC		***Hind* III**
	MaGWD1-pro-GUS-R: gaccacccggggatccGTTCCTGTATCATATACCAACTC		***BamH* I**
	MabHLH6-pro-GUS-F: tgattacgccaagcttTGAGAAGTTGGTGGTGGAGT		***Hind* III**
	MabHLH6-pro-GUS-R: gaccacccggggatccCTCCGCGAGGTCGGCAATGC		***BamH* I**
**EMSA assay**	MabHLH6-pGEX-F: ggttccgcgtggatccATGAGAAGAGGCCAAGCAACTG		***BamH* I**
	MabHLH6-pGEX-R: agtcacgatgcggccgcTCAAAGCTCAATTTTCATTTG		***Not* I**
	MaGWD1-probe-F: TCTAATCACATGATGGTGTGTGGCCGCCATCATTCTCTCCCAAGCAACCACGTGTAAAC		
	MaGWD1-probe-R:GTTTACACGTGGTTGCTTGGGAGAGAATGATGGCGGCCACACACCATCATGTGATTAGA		
	MaGWD1-mprobe-F: TCTAATaccagtATGGTGTGTGGCCGCCATCATTCTCTCCCAAGCAACaccggtTAAAC		
	MaGWD1-mprobe-R: GTTTAaccggtGTTGCTTGGGAGAGAATGATGGCGGCCACACACCATactggtATTAGA		
	MaLSF2-probe-F: GCAAATATCACGTGCGGGTTAAGCCCGAAATATTAGAACTCGACGACATGTGCTCCAGA		
	MaLSF2-probe-R: TCTGGAGCACATGTCGTCGAGTTCTAATATTTCGGGCTTAACCCGCACGTGATATTTGC		
	MaLSF2-mprobe-F: GCAAATATaccggtCGGGTTAAGCCCGAAATATTAGAACTCGACGAactggtCTCCAGA		
	MaLSF2-mprobe-R: TCTGGAGaccatgTCGTCGAGTTCTAATATTTCGGGCTTAACCCGaccggtATATTTGC		
	MaBAM1-probe-F: CGGCGCGCTATCATCCATGCCATCTGCGTTACCACACGTGTCGCTGCCGGAGCCGTCGG		
	MaBAM1-probe-R: CCGACGGCTCCGGCAGCGACACGTGTGGTAACGCAGATGGCATGGATGATAGCGCGCCG		
	MaBAM1-mprobe-F: CGGCGCGCTATCATCCATGCactcgtCGTTACCAaccggtTCGCTGCCGGAGCCGTCGG		
	MaBAM1-mprobe-R: CCGACGGCTCCGGCAGCGAaccggtTGGTAACGacgagtGCATGGATGATAGCGCGCCG		
	MaBAM2-probe-F: GCCACCGGATGCAAGTGGGGTGGCATTGCCGATCTTGGTTTTCCATTTGGATGGCGCTG		
	MaBAM2-probe-R: CAGCGCCATCCAAATGGAAAACCAAGATCGGCAATGCCACCCCACTTGCATCCGGTGGC		
	MaBAM2-mprobe-F: GCCACCGGATGacaggtGGGTGGCATTGCCGATCTTGGTTTTCacttgtGATGGCGCTG		
	MaBAM2-mprobe-R: CAGCGCCATCacaagtGAAAACCAAGATCGGCAATGCCACCCacctgtCATCCGGTGGC		
	MaBAM8-probe-F: GACCAATGTAAACGCTGTATGGCGAGTGCCACGTGGATTTTTTAAATTAACCAATAAAA		
	MaBAM8-probe-R: TTTTATTGGTTAATTTAAAAAATCCACGTGGCACTCGCCATACAGCGTTTACATTGGTC		
	MaBAM8-mprobe-F: GACCAATGTAAACGCTGTATGGCGAGTGCaccggtGATTTTTTAAATTAACCAATAAAA		
	MaBAM8-mprobe-R: TTTTATTGGTTAATTTAAAAAATCaccggtGCACTCGCCATACAGCGTTTACATTGGTC		
	MaBAM10-probe-F: TCAAAATATATGGTTATATTTACTTCACAAATGATATTGTTTGGGACAACATACTCTCT		
	MaBAM10-probe-R: AGAGAGTATGTTGTCCCAAACAATATCATTTGTGAAGTAAATATAACCATATATTTTGA		
	MaBAM10-mprobe-F: TCAAAATATATGGTTATATTTACTTCAacaagtATATTGTTTGGGACAACATACTCTCT		
	MaBAM10-mprobe-R: AGAGAGTATGTTGTCCCAAACAATATacttgtTGAAGTAAATATAACCATATATTTTGA		
	MaAMY3-probe-F: TACGTTGCCAACTGGATTAGACATGTGAGGGGGTCCCAATTACACATGACAGGTCCTA		
	MaAMY3-probe-R: TAGGACCTGTCATGTGTAATTGGGACCCCCTCACATGTCTAATCCAGTTGGCAACGTA		
	MaAMY3-mprobe-F: TACGTTGCacacgtGATTAGAactggtAGGGGGTCCCAATTAaccagtACAGGTCCTA		
	MaAMY3-mprobe-R: TAGGACCTGTactggtTAATTGGGACCCCCTaccagtTCTAATCacgtgtGCAACGTA		
	MaAMY3C-probe-F:ATCGTCCGCAAATGTTGAGGTACGACTGCCATTACTGCAGCCTCCATTTGAGCCTTCC		
	MaAMY3C-probe-R: GGAAGGCTCAAATGGAGGCTGCAGTAATGGCAGTCGTACCTCAACATTTGCGGACGAT		

	MaAMY3C-mprobe-F:ATCGTCCGacaagtTTGAGGTACGACTGCCATTACTGCAGCCTCacttgtAGCCTTCC		
	MaAMY3C-mprobe-R: GGAAGGCTacaagtGAGGCTGCAGTAATGGCAGTCGTACCTCAAacttgtCGGACGAT		
	MaISA2-probe-F:CGAAACGACCACCTTTCTTCGTTCACGTGATTCCCAGGTGCGTTGTCTTCCTCCTTGG		
	MaISA2-probe-R: CCAAGGAGGAAGACAACGCACCTGGGAATCACGTGAACGAAGAAAGGTGGTCGTTTCG		
	MaISA2-mprobe-F:CGAAACGACCACCTTTCTTCGTTatcggtATTCCacgggtCGTTGTCTTCCTCCTTGG		
	MaISA2-mprobe-R:CCAAGGAGGAAGACAACGacccgtGGAATaccgatAACGAAGAAAGGTGGTCGTTTCG		
	MaISA3-probe-F: TTAGGAAAGTGATAACCTGTAGCACGTGCTTGGCACGTGGGAGGAGTGGGACCCACGGA		
	MaISA3-probe-R: TCCGTGGGTCCCACTCCTCCCACGTGCCAAGCACGTGCTACATGTTATCACTTTCCTAA		
	MaISA3-mprobe-F: TTAGGAAAGTGATAACATGTAGaccggtCTTGGaccggtGGAGGAGTGGGACCCACGGA		
	MaISA3-mprobe-R: TCCGTGGGTCCCACTCCTCCaccggtCCAAGaccggtCTACATGTTATCACTTTCCTAA		
	MapGlcT2-2-probe-F: TTGGTCACGTTATGTTGTGATATTTAACGAACACATGAGATTGAGATCACACCGACGGG		
	MapGlcT2-2-probe-R: CCCGTCGGTGTGATCTCAATCTCATGTGTTCGTTAAATATCACAACATAACGTGACCAA		
	MapGlcT2-2-mprobe-F: TTGGTCACGTTATGTTGTGATATTTAACGAAaccagtAGATTGAGATCACACCGACGGG		
	MapGlcT2-2-mprobe-R: CCCGTCGGTGTGATCTCAATCTactggtTTCGTTAAATATCACAACATAACGTGACCAA		
**ChIP assay**	MabHLH6-pET-28a-F:aatgggtcgcggatccATGAACTCTGGGTCGTCGG	MabHLH6-pET-28a-R:gacggagctcgaattcTCAAAGCTCAATTTTCATTTG	*BamH* I<br>*Hind* III
	MaGWD1-qchip-F: CGCCACCCCGTTATCACCTT	MaGWD1-qchip-R: TAAACCATCGCTCCTCACCC	
	MaLSF2-qchip-F: CGGAGGCAAAGGCGAAAG	MaLSF2-qchip-R: GTGATGGAAGCACCGAACAG	
	MaBAM1-qchip-F:GCAAGAGTCCGCAGAAGAAC	MaBAM1-qchip-R: CAAAGATCTAGCAAACGACG	
	MaBAM2-qchip-F: CAGTGCTGGATTGTTGTGCT	MaBAM2-qchip-R: GACAAGAGACCGATCACTAC	
	MaBAM8-qchip-F: GATTTCTGAGGGACATTCTT	MaBAM8-qchip-R: AACTGCCTGTTACTGGTGATA	
	MaBAM10-qchip-F: TTGCTCAATCAACTCCCTCT	MaBAM10-qchip-R: GTCCCAGACGACATCATTTG	
	MaAMY3-qchip-F: GAGAGGACAGAGGCTTTGAG	MaAMY3-qchip-R: CAGCATTTCCCAGTGTGACC	
	MaAMY3C-qchip-F: TGAAACGCAATAAAGCAGGC	MaAMY3C-qchip-R: TTGGTCGGGTCTTAGTGGTC	
	MaISA2-qchip-F: GGTCCCACCGACGAAACGA	MaISA2-qchip-R: TGCTCTCTCGGTCACAAAAT	
	MaISA3-qchip-F: GGAAAGTGATAACCTGTAGCAC	MaISA3-qchip-R: ACCAATGAAAGAGCAGCGAGAG	
	MapGlcT2-2-qchip-F: GACGTATCTGTGCCGTAAATC	MaGlcT2-2-qchip-R: CTGTCACCTTCCCTGCTAAT	
**Dual-luciferase transient expression assay**	MabHLH6-pEAQ-F: caaattcgcgaccggtATGAACTCTGGGTCGTCGG		*Age* I
	MabHLH6-pEAQ-R: agttaaaggcctcgagTCAAAGCTCAATTTTCATTTG		*Xho* I
	MabHLH6-pEAQ-PBD-F: tcgccgaccggtaggcctATGAACTCTGGGTCGTCGG		*Stu* I
	MabHLH6-pEAQ-PBD-R: aaccagagttaaaggcctTCAAAGCTCAATTTTCATTTG		*Stu* I
	MaGWD1-pro-LUC-F: tatagggcgaattggCCGCCACCCCGTTATCACC		*Kpn* I
	MaGWD1-pro-LUC-R: ttggcgtcttccatggGAGTGACTCGCGGGAGAGAGAG		*Nco* I
	MaPWD1-pro-LUC -F: tatagggcgaattggGTGGTCTTAGTTTGTTGTGAAG		*Kpn* I
	MaPWD1-pro-LUC -R: ttggcgtcttccatggCGCAGATTACGAGAGGGTTA		*Nco* I
	MaSEX4-pro-LUC-F: tatagggcgaattggGATCAGATGTTATGGGAGGT		*Kpn* I
	MaSEX4-pro-LUC-R: ttggcgtcttccatggCCACATAGTGGGTGTGGCTG		*Nco* I
	MaLSF1-pro-LUC-F: tatagggcgaattggGGCACTGATTTGAGTGCTGC		*Kpn* I
	MaLSF1-pro-LUC-R: ttggcgtcttccatggTGGTGTGGGTCGGAGCTCGT		*Nco* I
	MaLSF2-pro-LUC-F: tatagggcgaattggCCGTCCGGGTTCCTTGAGG		*Kpn* I
	MaLSF2-pro-LUC-R: ttggcgtcttccatggCCCCGCCTCTTCCTTCTCTC		*Nco* I
	MaBAM1-pro-LUC-F: tatagggcgaattggGAGAAACCTTGATGTTAGAGAG		*Kpn* I
	MaBAM1-pro-LUC-R: ttggcgtcttccatggCTCTTGCTAAATTTACTATCTT		*Nco* I
	MaBAM2-pro-LUC-F: tatagggcgaattggACCTTCGGCGCAGCCGATCC		*Kpn* I
	MaBAM2-pro-LUC-R: ttggcgtcttccatggCTGCCGCCGCAGTTCTTCC		*Nco* I
	MaBAM3-pro-LUC-F: tatagggcgaattggGCCTCCAGTACCTAACATTC		*Kpn* I
	MaBAM3-pro-LUC-R: ttggcgtcttccatggCGCGCCACTAAAAAGCCCT		*Nco* I
	MaBAM4-pro-LUC-F: tatagggcgaattggTCGATCATGTCCCTTATCTC		*Kpn* I
	MaBAM4-pro-LUC-R: ttggcgtcttccatggCTCTCAAATTATAGAAGATGT		*Nco* I
	MaBAM7-pro-LUC-F: tatagggcgaattggCAAGATTTATGGGAAGTGACG		*Kpn* I
	MaBAM7-pro-LUC-R: ttggcgtcttccatggAGCTGCAGCCAAAACGTGAA		*Nco* I
	MaBAM8-pro-LUC-F: tatagggcgaattggGCTTGGAGCCCATGTCGTAG		*Kpn* I
	MaBAM8pro-LUC-R: ttggcgtcttccatggTCGAGTTGGTTCTCGAGGG		*Nco* I
	MaBAM10-pro-LUC-F: tatagggcgaattggGCTCAAGATATCAGCTAAGAGG		*Kpn* I

	MaBAM10-pro-LUC-R: ttggcgtcttccatggTCGAGCGGGTTCTTGAGTCG	*Nco* I
	MaAMY2B-pro-LUC-F: tatagggcgaattggCGTTTCCATGAGTGCCCGG	*Kpn* I
	MaAMY2B-pro-LUC-R: ttggcgtcttccatggAACTGATAACTAGATGATTATCAG	*Nco* I
	MaAMY2C-pro-LUC-F: tatagggcgaattggCATCTAAACGATCCATCGCG	*Kpn* I
	MaAMY2C-pro-LUC-R: ttggcgtcttccatggAACTGATAACTAGATGATTATCAG	*Nco* I
	MaAMY3-pro-LUC-F: tatagggcgaattggCGGAATCTGTTCTCCCGCAC	*Kpn* I
	MaAMY3-pro-LUC-R: ttggcgtcttccatggCGGCGAACGGTGGGGGAG	*Nco* I
	MaAMY3A-pro-LUC-F: tatagggcgaattggGAGGGTCCGTGAAAAGGATA	*Kpn* I
	MaAMY3A-pro-LUC-R: ttggcgtcttccatggGATGATCTTTGATTGTTTCC	*Nco* I
	MaAMY3C-pro-LUC-F: tatagggcgaattggGCACAAATAAATTATGGTGCC	*Kpn* I
	MaAMY3C-pro-LUC-R: ttggcgtcttccatggCTTCTCCTTGTCTAAACTCTTC	*Nco* I
	MaISA2-pro-LUC-F: tatagggcgaattggCACCTGTGAGCAAGGACAAG	*Kpn* I
	MaISA2-pro-LUC-R: ttggcgtcttccatggAGAGCAGCCTTTCAGGCCTC	*Nco* I
	MaISA3-pro-LUC-F: tatagggcgaattggGAGGCATGGATCCGAGGCTT	*Kpn* I
	MaISA3-pro-LUC-R: ttggcgtcttccatggTTGAGAGATCGAAGGAAACA	*Nco* I
	MaPHS2-pro-LUC-F: tatagggcgaattggTGAGTGGGATGCTACCTGAC	*Kpn* I
	MaPHS2-pro-LUC-R: ttggcgtcttccatggGGGAAAGCTCCCTAAAGAATC	*Nco* I
	MaMEX1-pro-LUC-F: tatagggcgaattggGGTGGAGAATATCTGGAGGT	*Kpn* I
	MaMEX1-pro-LUC-R: ttggcgtcttccatggTTTGGAGGGAGGGAGGCATG	*Nco* I
	MaMEX2-pro-LUC-F: tatagggcgaattggGCCACATTGGATTCAAATGTC	*Kpn* I
	MaMEX2-pro-LUC-R: ttggcgtcttccatggCAATCAGAAGTCTAGAGAGAG	*Nco* I
	MapGlcT2-1-pro-LUC-F: tatagggcgaattggCCTCAAAGATCTGAGAGCTC	*Kpn* I
	MapGlcT2-1-pro-LUC-R: ttggcgtcttccatggCGCCTATCGGCTTCAGATCC	*Nco* I
	MapGlcT2-2-pro-LUC-F: tatagggcgaattggTGATTACAACAATCTTGAGCC	*Kpn* I
	MapGlcT2-2-pro-LUC-R: ttggcgtcttccatggCTCGATCGGCGCAGAATCTC	*Nco* I
	MapGlcT4-1-pro-LUC-F: tatagggcgaattggGGCGGTGGTCGGAGGAGGAG	*Kpn* I
	MapGlcT4-1-pro-LUC-R: ttggcgtcttccatggCTTTCCCTTCTAATCCCCTC	*Nco* I
	MapGlcT4-2-pro-LUC-F: tatagggcgaattggCGGCTTGTGGGTGCTCACG	*Kpn* I
	MapGlcT4-2-pro-LUC-R: ttggcgtcttccatggCGACAACCCGTTCGATCTTG	*Nco* I
**Y2H assay**	*MabHLH6-DBD-F: catggaggccgaattcATGAACTCTGGGTCGTCGG*	*EcoR* I
	MabHLH6-DBD-R: gccgctgcaggtcgacgTCAAAGCTCAATTTTCATTTG	*Sal* I

Supplemental Text S1. Nucleotide sequences of the promoter of starch degradation enzyme genes. E-box (CANNTG) is marked by red and border. Translation start site (ATG) was shown in yellow box.

MaGWD1 (XP_009392286.1) (Ma03_p15660.1)

TCCACCGCCACCCCGTTATCACCTTTGTTTCTTTCTTTGGGTTGTGGGTCCGCCGCCCATGGGAATTTGCCCAAAGACTGGACCTTCCAACGACACCCCATCTCTCTATGCATTTAGAGAAGCTA
AGTGCGAGGAATAACGAGCCTTGTTCTTGGAACAAGCGCCATTGCAGATGGGGTGAGGAGCGATGGTTTACATGCTAACTCAGATGTATCTCGGATTCGAATTTGTCGGATCAATTATTTTAAT
TAACCTGAATTTTCTTTTTTCCTGTCATAATTATCAAGTGGTCTGTATAAGGCACTCATTCGATATCGATAGATGTTCTTCTACCCTTCCCCCCACTTGCACAAATATCACGGATAGTCACAAC
CTTCCCGTGCCGTGCCTCCCACCCCTCGTATAACAATCTAATCACATGATGGTGTGTGGCCGCCATCATTCTCTCCCAAGCAACCACGTGTAAACAAAAATATCACGCTAAGACTCCTCTATTG
TCGATCATCCTTACGGTGGGGCTCTCCTTAACTTACTCGTGTCCAGAACCAGCACCTCATCCTCACACACCAACACACCATTCTCTTCCACTACCAGCACACCACGTGGTAAATCCGGTTCACTC
CGCCCTGGCATCCTGAGGTCGTCCAGGAACCAGGGATGTCCCTCCAACGAAAGTATGCTACGTGAGCCGTACCACCCGGTTCTAGTCACGTGCGTGATCGGGGCGTTGATCTTTTCCCCGCCTCT
CTCTCTCTCTCTCTCTCTCTCTATCTCGCTCTCTTTCTCTCCCTCCTGCGAGTCACTCTGCGCCTCTCTCTCTCTCCCGCGAGTCACTCTGCGCCTCTCTCTCCTCCGTCGTCGTCGTCCTTCTT
TTTCGGTTTCTTCCGCCAATCACCGCATCGCTTTCTTCTTTTCTCCTATTAATACCCTCCGAAGTAGAGCAAGCAAGAAGCTCTTCCTTCAGGAGGAACGTCTCTCCCTCTCTCCACTTCTCACA
CCCCCGCCCCCGACTCAGCGAATCGACATTCTTGGCGTGTCAAGGAAACCTTTCTTTTATTTGCGAAGGTAAATTTCTTAAGTGAATTCTTCGGGGTTCGAAAACCATAATCTCCTCCTCGTTTA
TTTATACCAGGAACAAAAATTCAGAGATCAAAAGGAAAAAATAATTAAGTCTCAAGAAAAATTGAGCTTATTTGTTTATGGCCTTCTTCGCAATAATTCTAGGGTTCGCCCTCGAGCATTATATA
AATCTTAGTCACACTTCCATGACCATTCGTCCTCCTGCTTGATGCGGTCCCTGGAGCTGCAAAGTCCGATTTTAACGTTTCCTGCTCGTTCCGGTTCTTGGTTCCGCTGGACATGAAATAAAGCC
GGGCCAGTAGTCTAGACAAAATTTTGATTAATCTTACTTTCTTCATGTTCTTTTTGTCGGAACTGGTGGGTTCAATTGTCAATCTCAACTTCTTGATATTTTTGAATTTTGCAATGCCAAGAAGA
TCCATGTTCGATCTGATCGGATGTCCTTAAGATTAATTAGCTTCCGTTGAGTTCGTTGATTATGACATTCTATCAAGTCCATGTACTCAAAGTTCAAATATGTGATTTCTAATTATGCTTAGGTC
CCATGTCCCAGAATTCCTTACGGCCACAAACTGTTTCAGTAAAGTTTTTGTTTGCGCTTTATGAATATTTTCATATATCAGAATGGTCATGAATTTTTCGTGAGGACCAGAAATACAACTTTCTG
ATTGAATTATTGGTTTTGATGAGATCCTTCATAGCTTTCTCCCATGCAGGGTACCCTGCATAGACTTTGCATTCACCTGTCCTTTTTATTTCCTACTATATTATTTCTAGAACTAGCACCAAGGT
AACTTCTAGTTCGTGTAAAGCGCTACATTCAATTTGAGCTTCAGTCTGCATTTAATTACCTTCTGAAGTTGTTATTGCTGGGTTTAAGAGTTGGTATATGATACAGGAACATG

MaPWD1 (XP_009416602.1) (Ma09_p07100.1)

CTACTGGCTCTTTGGGTAAAGTATTGTTACATTAGGAGTGTTAATTTCAACTACTTTTTTTTGTGGTCTTAGTTTGTTGTGAAGATTAAGAAATGCTTTATTCTGCTTCTAGAATTATTTCAAGG
GCTATTTTTGGAGCTTTAGGTTGTATTGTTTTTGGAAGTAATATTATGGAGTTTCGTATACGGTTTCTAATAAGCAAAATTTTGGAATATAGTGACCAACAGATGCATAAATTACAGGAAGTTAC
TGTTGGTTTGATTAATGGCGCATACCTTGATATTGATAAATTTACCTGTATAGTAAGTGTTTTGAATCTTCCAAGTACTGATTTTTAATAGGTGCCTCATGCCATATGTTGCATAAATTTTGGTT
GATTTGCAATATCTGAGAAATGCTTTATTTTGTTGCTTTGGCTGTCCAGGGACATTGTGTGTCCGGTTAAGTTGTAATTTGCCATAAATAATTGACACAAAAGTTACAAGTTAAGTTTGAATGAG
TTCTTCAAGTTAATCGTGTAAAAACCTACTTTTGGTGAGTAACACAATTCAAAAGCAAATAGATAAAATTTGTGGACATATTTAACCAAAAATTGAAATGCTAAATTGAACTCTGTACAGCCAAA
TTAGAACTGGTAGGATGAATGATGGACCCTTTGTGCAATCCTATAATGCCAATTTACAATCGTATATGTGTTTTGCTCTATTAACTGCCAGATACAACTCAGCTCAGCTGACAGAAACCCAATAT
CGAAGTTCTTTGCAAAGAAAACTGATGATAAGGACCAGATGGAGGTAAAGCATGGTAAGTCCCTCAAGGAATCTCCGAAAAAAGAGATTTTTGATATAGCTGCGGAACTCAGTATATCATCTGA
AGAATCGCCACAAGGAGATCACTTCGATGATCTGAAAGAACATCTGGAATTCAACACACATGCAAATGCTGATGAGTCTGATCACTTCTCCCTGCTCAAGAATCCTAGCATAGAACCAGAAATT
TGTGGCACCAAAAGAGGCTCCGGGGCTATTGCACCTGATTCTGGACTAACATCTGAGAAGGGCTCTAAACCTAAAAAGAAGGCACGGCCTGTTAAGAATACTGGGGACAAGCAAGCTTCCCTCC
TATCATATTTTGAGAAGGCCTAGACGTTTTTTTGTTCAGTATATTTTGATGCTTGAACATGTTCCTTGTACAACTGCAACAAGTTTGCTTGTAATTGTCCTTTTACCTGTGAAGTCCAAACACTA
ATCAGTGACCTGGTAACTATATACTACAAGTGTTCTTAGCTCACAAGAAGCCGGAAGTGTAAGCTTTTCTCGTGTGCTTTCATCTCTTGAAGCTTCTGTACTTTGTGCTGGAGCTTTCTTTTCTC
TGTTCTCATGTCAAGCTTTCATGGTTACATGGTGTCACCTTTGCTTCTGATATTTCTGACTCTGTTTTGTAGACGAGCGTTACGCAGCGTTGTTTCGTTGTCTGGTGGCGGGCGGAATCAGCCGT
GAGGGACCCGCACCGTTGTTCTTTTTCTTGCTGTCCGTCCATTGAAGCCATCTCGTTTCTTCAAGGATGAGCTCGGTGGCCTGTCATGTCCAATGCTATCAACTTGCGGAGGTAAGCTAACCCTC
TCGTAATCTGCGATG

MaSEX4 (XP_009413138.1) (Ma08_p13120.1)

ATCAGATGTTATGGGAGGTCAATATGTCAATTGGGTAATATACCGAACGAGAGGACGATACGTTGAAAATTTAGACGAAGCGTCAGATGAACCAATGACATATCGTGTAACATAGGATTTGTAT
TTGTAATTGATTGTAACTATGCTAACTCAATTAGAAGCTCATTGAGTTTGTTCTGGGATTGAGTTGGGCTCGTTACGAGGCTCATTCAATGACTCAAAGTTAGGTCAGGTTATGGCACTGTCAGA
AAAGACGATAGTACCACTCAAACACAATCTTCCAAACTGTGTTAAACAATGGTACCACCCAGATTGCAAAGTGGTACGAATGTTTTTAAGTTAAACATATTTTCTATATTGGTTTGATCGAACGA
AGCTTTCAAATCATTGTAATTTTTACGAAGCACTAATTAATTTCTCTCCTTGGTGCCGATAAGATCATAATAGAATTATGATTGAAATAAAATTTAATTTTTTTATGATTAAAATTTGAAGTTGA
AAAACATAATTAAGGAAATAAGCATTTTTAGGTGAAACTCCACACATACACAAATATATATATATATATATATATATATATATATATACTATTTTTGTTGGTCTCATTCCGGTACTATCGTTTGATT
ATCAAGTAAAACATCTCTCATTAAAACATCTCACTATGATTATAAAAATAGATATTCACATATTTTCATAACTGAAATTAGTTAATCTTAAATCTTTACTAACAAAGATTAACCTGAATTATTTA
GATGAATATTAATATTATATATATATATAATTTTAATATTTAGAGTGATACTTGCTATATCGAAATGTATAAGAACCCGCTTTTTGTTGTATCTTATTTTATTTGTGTTGACGAACCCTTCATGA
AGTAATAGCCCGCAGAGAATCCGCATTCATCCATCCAAACATTTGTTTGGTTCAGCCACACCCACTATGTGGATG

MaLSF1 (XP_009416558.1) (Ma09_p06790.1)

GCGTGGGGTCTAATGCAGTCAAAATTATGCTTTTAAATTTCGTGAAGAGCTTATTAGGCACTTATTAAACACAAAAAATTATATTATACAAAGTTATTTTCTTAAGTATATAAAATATTCTTCTT
TCGAATCGAAGTAGAATAAAATAAAGATATTTTGAGTCACGTTAGAACATAGGAATTCAATTGTACTTGACGTTTCAATCCAATGAAAGAAACTTGCACTAAAAAGCAATATATAAGGAATAGAC
TACAAACAGGAAATAAGGTGAACTGTTGTCATCAACCAAATACGTTAGCCATTGTGCATTATAATTTCATCAAAGGCAAATGAACATCTAAGCCACATTTAGAGAAGGAAAAAAAGGAGAGAAAC
TTCTGAAGCAAATGAGTCGCCGAGTGCTCGCCAGCTCGCACATCGCCCAACGAGATCACGACGCTGGAAAAGGTTTGTGAAGGCACTGATTTGAGTGCTGCATGATGACAACGATGAGGCCAATA
ATTTGATGTCCTGTTTGATTCCTATTTCGCTTCTAATTTAGAGATTATAATTCATCGATAAGGGCTTCGACGTGACACGTGTTTAAACTTAGATTTAATGCATGATTAATAGACAATTCCGAGAA
TATTTCATATAAACGTAGCTCGATGGTGCACTTGTCATATGTCATGATTAAGATGGATACTGCTATTTGATCACAAGATGCAAACAGTGCACTCAAACATAATAATCGTTCAAGTAGGCATCAA
TTACTACAGGAGAAAGAGAGTATGATAGAAAACGACCATACACTACTTTTACTAAATACCTCCAAATTGTTTGGAAAGCATTATATTAAAATATTCTTAATATATTTTAATTCATAAGGAATATA
TATATATATATATATATATATATATATATATAGACTTTTTGTTATAAGGATGAAGAAGAAAATATATTCTGTTTCACTATATTATAAGGATTGTCTTTAGGACGAATTTTCAGAAAAAAATATTCTCT
TTTTAGAGTTTTCCGTATAAGTATCTAATATTATAATTTCTCATAATAACTTGAAATATCATTTTTTTCTCCCTTTTTCTATGAACTAATATAGTCACAATGTTTTTACACTACGTTATAATAT
TTTCAGTAATATCAAAAAATACTATAATTAAATATTTTCTTATAAATTTAGTAAAACATTATGTATATTATTTTTGGGTAATAATTATGATATTTGTTATTATTACTAAAAATATTATAGCATAA
TATAAAAATAAGATCGGTTCACCCTATGTACTGTCCCATAAGAAAAAAAAAAAAGGAAACAAAATAAAAGGAGATATATATTATGGAAAATAATAGTGTTAGGAAATTCTGAAAATTGGGATAGAA
AAGTTCGGAAAGAAAATTTTAGAAATTCACACTTGTTTTCATATAATTATATATATTAAATAATATTAAACAGGTTTGAAGGAATAAAAATGTTATTTTGGAGATCTGAAAAGACGTGTGTGTGT
GTGTGTGAGAGAGAGAGAGAGAGAGAGAGAGAGAGGTTAATTTATTTTAGAAAAAGAAAAATAAATCTAAACATAGATATTTTTTTCCGCTCATGAGAGGGCGGCGGCGCGCACTACTGGACGTT
AGGTGGGGCCCACCGTGGCACGGTCTTCCCGTTGCGTCCCGTTCCGCCCCCTCCCGCTTGTCCGTCCACGTGGCTTGTGGATCAGAGACCGACGACGGTGTCGTCTCGTCCGCCCTCCGTCCCTC
CCTCACCACGTCCAACGTAACCGACTCACGAGGAGGTACTGCGAAGAAGAACTCTTCTCCTCCTCCTCCTCGCCCTGACGAGCTCCGACCCACACCAATG

MaLSF2 (XP_009395158.1) (Ma04_p00190.1)

CAGAACCCTGTGCTGATGGCCGATCCAACCTAAAATTACACGATTGTCAGCGAACACACTAAAATTACACGATTGTCGACGCAATTCCCAACTATTCGTATTACTTTGGTGGTAAGGGAAAGGGG

TTCTTCTGGGGAGTCTTCTTCCTGGCGCTCTTCGTTGAGTTCCGGCTGGGGCTTGAGGTTTCTCAGCACGAAGAAACCAGCCAAGGTTGCTGACAAAAGGATGAGCAGCACTCTCAGGGGACACA
TCTTTCCACTTCTCTCTCGCTCTCTCTCCTCTTCCTTGAGATGGACGAACTCCGTCCGGGTTCCTTGAGGAGGATGAGAAAGAGGTTGCCAGCTCGACGGCCGCCTCCCAACACGAGTTCTTCTT
TGTTTGACTTGGAACGAAACACCCATGGGAGGGTGAGTGCGGCCCACACAAAAGGAGTGGTTGGATATGGTGGGGCGTTAGGAGGACATGAGTCCGCAGGGGCCGAGTCGAATCTTGGCGGTGTC
CAACCACAGCACTAAAAGAAAAGAAATGGATGTTCTTTCTAATGGGGGTTGAAGGAATGACGAGGGTTCCACCAAGAGACATCATGTGGCCAAAGGTGGGTCAATGGACTTTTTGTTTTAAGATG
CAACTCGACATGCTTTGCTTTGCTTTGTAAACGAGCTGTGTTCTTCTCGACTCGACGACCCACGCAACACTCGATTTAAGAGTCTGAATCACTATTTTTTTTTCTTTTCTAAAAAATAAAATAA
AAATTAAATTTATAATAAATATATTTATTGAGTATAAATTATGTTGGATGAGGTTTCATAATTAAGTTTGAAAAGAGTTATTTATACAAGCTGGATGGAGCCTTTTTCCTTGGTCAATTATACGA
ATGAATGAATGGCTTGCTGATTCAAGTTGGATTGCATTTTGCATCAGAACAATATCTTTAAAGATAAAAAAGACTTCGACACTAGAAATATTAAAAAAGATTCAATGTAATGGCTCAAATCAATG
GGCCTGTAGAATGGGCTCAAATCAATGGCTCAAATGAATGGGCCTGTAGGCAGCCCAACAAGCCATGTAATGGCGCCAAATGTTGTCCTGTCACGACGCCACCGTCAAGTTTCCCGCCAGAAAG
GTTGCAAATATCACGTGCGGGTTAAGCCCGAAATATTAGAACTCGACGACATGTGCTCCAGAAAATTCTACCAAAAATATCTCTCTCATAATTATATTTCATATTATACTTATCGTTTGCACAA
CAACAAAAATAACAAATAAGATCGTAATTTTCCAGCTATTTAAATCTATATTCTTAACTAAAATAAAAATATTTAAATATTTATGATATGAAAATTTATATGCCCGACCCTTACTAATTGTTTCA
TATATATATATATTATGTGTGGTGGTTAAAATCAAATATAGAGATAAATTATGAGGGTGAATATACTATTAATAGTAGAATTATTTATCTTTGTTCAACTAAAAGGGTCTAATTGGTTACATTAC
ATGGCTATAAAGTGACTTTACAACGTGGTCTCTTCCGAGGCGGAGGCGGCGGCGGAGGCAAAGGCGAAAGGGTCTGTGACGAGAGCGCGGCGATCGAGAGAGACAGAAAGAGAGAGAGAGAGAGA
GAGAGGAGAGAAGGAAGAGGCGGGGATG

MaBAM1 (XP_009398630.1) (Ma01_p10710.2)
GAGAGAAACCTTGATGTTAGAGAGAGTCATAGAGAAAGAAAATTAGAAGAGAGAGAAACTCTAAAGAGAAAATATAAGGAGAGAGAAAATTCGAGGAGAGAAAATTCTTAAGGGAGAAAAATTAC
TAGATCACTATAACATTGATTAATTGGCAGAGAAAGATCAACATATTGTAAATTTGAGTACTTAAAAAAAAAAGGATTGAAAGCATGGATGATGATTTATTTTGAATATTATCACCTAGATTTAA
TGGGAAAAATATTAAAGATAAAAAAATATGATAAAAATTTTTCTTGGGTCAGAATGTTTAAAAGCAATTATCTTGAATAGTTTTAAAGATGTGATAAGCAATACATATTTTAAGTTATTCTTTGA
AGAATAGAAAGAAAAGTTTATTTTTAACTAAGAAGAAAATAAAAAAGCATTGAAACTTTTCTCTTAATCAATACTAGATGAAAGTATTATTTCATGAATCTCTAAAACATGAAAAGAAACTTGGT
TGATATTAGAAGATATTTATAAAAGAGGTATATATCCTTTCATGAAGATAAATTAGAAAAATTGTATCACATAGAATTTGAAGACAATGAAAATATCTCAATTAAAGATATTAGGATTAAACAAG
ATTTAGACTACGACTTAGATAATATTAATTTTCTTAGAGTAGATGATTTTAATTTAATTAGAAACATAAAAGTGGTTTTACCTCCATATATAAAAGATTTTACTCAGCTAGTAATTAAAGATTTA
GAAGACGATAATTAAATTAAGAATTATCGAGCTAATTTTGAGGAAGAAGATAATTACAATAAATTTGAGCATAACTTTTGTATCTCATCAAATTGAAAAGTAAGAATTAGTGTAAACATTTCATC
CATCATCTTTATTAAAAATTTTATAGGGTATTAGAGATTTCCTAATAATCTCAATTATAATGTCATTTAGTCCTATATTGGGCTATCCATTTAACACACACTATCACAACACGTTAAGTGCAAAA
GAAGCTACCACGAAAGTCGCGCTCAAGCGTGCCTCACTGCCGCTGGCCCTCATGCCATCGGCGCGCTATCATCCATGCCATCTGCGTTACCACACGTGTCGCTGCCGGAGCCGTCGGATCCTCA
GCCCCTCCCGACTCATGCGAAGGCGTCCGTGCCAATCATAAACCCAAACACGCGGCCGCACACGAGCGAAACCCTAGTTTCCCCCCATACCGCCCTCCCACGGTACCCCCCGCCTCCGGCCGCCG
CCGGCACCCGTCTGCCCGCCGCCGACGCTGCCCGAGGCCCCGCTGGCGGAGCGTGTCGAGGGATCGCCTGCGTCCCGCGATCATCCGAGCCCAATCGCTAGAGGACGCCGGAGCCGCCTCGGAGC
CCCGCCGGGGAAATCTGGTCTCGTCCTCTGCCGGAGTGGAGGAGGACCTCCAGCAGAAGGATCCGGTAGTGGGGAACGAGCTCTCAACACCTCCGATTCGTCTCCCTCTCACCGATTCGATGTAT
CAAGAAAAGGACTGTCTTTTCTGATTTGTTCCGCTGAATTTCGTGACTTTGGCTTGGTTAACCGAGATCCAGTCGCAAGAGTCCGCAGAAGAACTCAAATCCACGTGGGAGATGGCGAAATATTT
TGGCAAATTCGCTCTTTTTGATTTCGTGGTACGGTTGTGCCCATGTTGTCCAAGCTTAGATCCGCCCAAAATGTAAGGGGCGTCGTTTGCTAGATCTTTGAAGCTCAGGGAAGGAGCAGAAGCAG
ATAAAGATAGTAAATTTAGCAAGAGATG

MaBAM2 (XP_009420599.1) (Ma10_p10330.1)
CGGTAGCGACCTGGTTTGGACCCAGTGCTTCTCTCAGCCCTCCGAGTCTCTCACCTACGCCGCTCTCCCCTGCACCAGTCCCCAGTGCCAGACTCTCCCTCGCTTCTCCTGCTCTCCTGATTGCC
ACTACAGCTACTCCTACGGCGATACTTCGTACACCAAAGGTGTTCTCGGCACCGAGACCTTCACCTTCGGCGCAGCCGATCCGGCGGCGATCACCGGCATCGCCTTCGGGTGCAGCACCGTGAGC
GAGGTTGGAGCGGAGAACTCACCCTTCTTCTCTAACTCCGCCGGGATCCTGGGAATGGCGAGGGGGCCATTGTCACTGGTGTCCCAGCTCGGTGAAGAAAGATTCTCCTATTGCTTCGCTTCTGA
CGACACAACCACCGCTCTGCTCTTTGGCTCTTCGGCAAATCCGAGCCCACAGGCTTCTTCCACGCCGTTCGTCAACGTCCCTTCTCCCCTATACTACCTCTCCTTGCAAGGGATCTCGGTCGGTG
CAACCCTCTTACCGATACCGAACACAACCCTTGCGCTCCAATCGAACGGGACCGGCGGCTTGGTCATTGATTCCGGCACCACCTTTACCCTGTTGACGGATCCCGCTCACGCGATGCTGAAGCAA
GCGCTTGTGTCTCAGATCGATCTACCGGTGGCGACCGTGGCCGGGTATGATCTCTGCTTCTCCTTGCCACCGGATGCAAGTGGGGTGGCATTGCCGATCTTGGTTTTCCATTTGGATGGCGCTG
ATATGGCCTTCCCGGCGGCGAACTACTTCGTTGTGAATTCCAGTGCTGGATTGTTGTGCTTGGCAATCTTCGGGTCTCCGTTCAACCTCTCCATCTTGGGCAACTTCCAGCAGCAGAACATGCAT
CTCGTCTATGACCTCGCCGGTGGAAAGCTGTCGTTCGAGCCTGCGAACTGTAGTGATCGGTCTCTTGTCGATGTAGTAACAAAAAAGTGATGTAATTGCAATTATTATGGGTTTCATGACGTAAA
ACTGTCTCATATTTGTCGTAATGCAGTATTTTTAAGTTTATTTAATTACTAATAAAGGTTTTTTTGTTGTATTAAGTCCAATTATTCAGACTTAATATGTTTCATCAAATCAAATCATTGGTTGT
TCATATCGTTCGTGCATTATTTAGTGATAGTTTGAGCCCTTCTCGAATTTGGAGGAAAGGAATATATGAGCTCGGAAAATTTTCTTGTTCGAACAATAATATGTGAACGATAAATTTTCATTTCT
TTTAGAGAAAGAAAGAGAGACAAATCCAAGTTGAGATCATCCACACCACCCATTCTCTGTTGGGTGTTGGTAAGAGTCCATGTTGAGAACAGAGCAATTGCATCGAGTTCTCCGTGCTGCAAGAG
CGAGCTATTCCTGATGACAAGCATCTCCTATGCAAGCCTACCTCATCTAGAGAGGTCATCGCCGGCGAGGACAGAGCTCCGCCTGTGGCACGGAGGGCATGAACTGGCTCCTGTGGGCGGATCGA
GAAAGATGGATAAGATAGAATTTGGTGGGAGATTCTCCTCAGATGGACCTTTTAAGAGAGTTACTGCACCTCAAACCGTGACCTCTGCTTCTCCGACCATGGTTTCTGATCTTGAGGTACGGTTT
AGTGCTACTGTCATGACAGTAAGAACTCGTCCATTCATGTCATTGCTGTAGATGTTCTGCTCGATATATACTCTGTTCATGTCTTTGTACTCAAGCCAAGCTAAGGATGGATCTGAGGCGCTTCC
GCCACACTGAACTCCATGCAGACAGCAGACTCTCGCACAGACACGGAGAAGATGCTGGCAAACTACGTGCCGGTGTTCGTGATGCTCCCAGTAAGTTGTTCTTAGTTAATTGGCCCAGCAGCAGT
GACATTACGGTCTTTGATCTGAGACTGCCCAACAGTTGGATGTCATCTCCGTCAGCAACGTGTTGGAGAAGCAGGAAGAACTGCGGCGGCAGATG

MaBAM3 (XP_009384530.1) (Ma02_p06550.1)
GCTGTTGTTCAGTCTCTCATAGACAATGGAATCGACGTAAAAGTTTATCTCGATTGATATTTTATGTGCTCGAGTACTCGATGACTGTTTTTCGGGTCAAAATTCCGAGAATTCCGGGTATCGAT
TTATCTTCAATGAGAATTTGGAGGTCCAATTTTTTGTTCTGAGTGTACTTATAATGGATTAAGGTAAGATGGAGAAAGTACCTTCGAGAGCTCAAGGTTTGTATCTGATGCCTACACAGCGAGAT
ATTGGAGAAAATTTAAATTTCAACAGTAGTTAACAGTTAATCTCCACCAATTCTCACTTTCAAAACCCCAGATGCTGATGGCTGTGGACTATTATTGTTGAGCCACTGAATATAAGATATTAGAG
GAAACTGAACCCTCCTTTTGATCGAGGTTCATGTTCCTCCAATTCTTACTTTCGGTGCCCTACATTGCTTATTCAAGTTTTTTGGCATGAACATTAGAGAATGTTGATCTGATTGTCTGATGTGT
TTGATTGCCTCCAGTACCTAACATTCAGAAAAAGTCAACCAGGGGAATTTTCATCCATTGTTTTCTTTTCTTATTTTCTAAGGAGACTCATAAAAAGGGTAAGGAAAAAAGAATGTAGAAAACAA
CAAACCCTAAGTCTCTTGAGAGTTTGGGGGTCCTATCATCATCTACTCTGAAGAGGTCTTTGTGGGCTGTAGTTGACTTTGTTCTGTAAAACTGCTCCATTTATAGATGATTCTTCACAGAGAGA
AGAAGTTCCATGATTCCGCTTTGCTACATACACCTTGCCCCATGCCAAGCTCATGACTTTGTCTGTCCTCTTTTGACTCTGGGTTGAAGGAGGTTGAAGTCACTTTTGATTGTAGATTGATACCA
AACCTGGGATTCCCAGGAACGAGAATGAGAGGTTAACGCCACACGTAATGCTTTGTTTCCATGCAATACCGTAAGCAAGTTTTGCAGCACCAAATGCTTGACGAATTCATGCTTTCCTGACCAAC
AAATCATAATCCCACTAAGCAAGATTAAAATTATAACTACGTTACTGGAAAATTAAAGCACAAAATCTTCCCTCTTATATCATATTAAATACACATTTTCAAGTTCAAATCTACTATTCTGAGTG
GAAAAAGATTGCCATTGCAGTCTTATTAATGCACTCTACCATATGGAATATTTAATCTTAAAAATTCTTCTCCAACCTAATAGAAAAAAAAAAAGAAGGAAAACATTCATATTTTCTACCTAGAA
AATAATTTTAATTTCTTAATATAACTCTAAAAGTTAATTTTTAGAGCTAGAAAGCTCATGAAAAAGCAAAGAAAGACCCAATTCGAAAGGTTGAATTGTAGTTCATGAATAATTAATTGACCAAA
GTTTCGAAAATCGAACAAAAAAGATTTTTAGTCAAAGAAACAGTAAACATCCATAGTCAATAAGTACCCTCAATCTAGTTAAGCCTCTATTTTTAGTCGGAGGACCCTCAATCTATTTTAAGTAT
CTTTCAATAACAATAACAAGTGGATTGATGGAAATGTGCGACAGTATATAGTGGTAAGGCGATCCTAAAATGAGGGCTTTTTAGTGGCGCGATG

MaBAM4 (XP_009399963.1) (Ma05_p07800.1)
TCAGCCTCTGAGTTCTCCACCTGACTATTCTATTTGACGTTATATTTAGCCATTGTTTGTTCTTTTGATAATGTCTAGTTATATATTTCTCCAATTCTGTTTATGTTGTTCATATTTAAGTGTCT
CGAACATGCATTACAGTAATCATTTGATAAGTAAATTATTAGGCTTTCTTTCTTTTTTTTCCTGTGTTTTTTCTGTTCCTTTTAGTATCTTGGTCTGAATAATCTTTCTACACCATCAATAAGCT
ATGTATGACAGCTTGACCAAAATATATGAATGTAAGTTTTTGAGTTCCAATCATGATATAATTTCTATATTAAGGGAATTATGAGAACATTTATATTGGAATTTTTGCCATGACCTAAAGAAGCA
ACTCTATGATCTGATTCTTACTACCCACTATAATATTTTTTTCACTAAAATAGAAATTTGCTCATGCAATTTCAGTATGGGAATACAATGTAGAATCACAAAAGTAAACTGAGCTAAATGTTTAC
CCCAACAATTGTTAGGCATCCAATACATTTTTGCAAAATTTGTAAGTGACTGCATCTCTATTAGAAGTCTGATGTTACGTTCACATTCCTAGGAACAACACTCGATCATGTCCCTTATCTCAGGT
CTAATGTATGAATCCTTGTCATATCATTCATGTGTTCATCAGAGAAATAATAGTGTTTCTTAAGATACATAGATTTTAAAGAACCATCACATTCTAATATGACACAGAAAATAACAAAATCTTGG

ACCGATAGGAAAGTATGGTTTTCCGTCACACCACACGCAAACTGAAGTGTTTGTCGTTGATGATTCTGCATCAGATTGGACGACGAGGGTTTTGGCACCACTTGTTATGTGGATAACTTGTACGC
CTGAACCAAATCACCAAACTCGATGATCAAAATGCCAACCATGAAGAGAGAAGCAACCCTAATGCGGACGACCAAATTTGCTGTGCAAAATGCCGTGGATGCATCACGAACAGTTCCCTACCGAG
TCCTACCTCGAATTGCGCGAGTGAGATGCCACCGACACGTCCACCTCCCCACATGGAGGAAGAGGAGAAGTCGCACTTGTCTTCCCCTCCTTCTCACCGGCCATTCACGTAATTTTTCGTCTCG
GCAAGAACAGTAAGTTGTTCGATCTGGGAATCGATCTTTCCGTACACTTGGCTGGAATGGGACTCCTTGGTGGACCGAGTAAGGAGGAGAACGAATCGGTCAACCTGCGCCGCTTCCGTCGGAGC
ACCCGCGCGCCCCGTTCCGCGCCATCCTGCGGGTTCTCCCCACCGGCTGCCCTACCGGATCATACGAACCCGCGCGTCCGATTGGCATCGGTTGGATATCGCTGTACGGTGTTGAGAGCCATCAG
GCTTCCTGAAGCTCCCGCTTCCCCTATCCTCAGCGCTAATACCCATAAAGTTGAAGAGAATCACCAGAAAGCCCCGCCACTTTCCATAGAGAAGGGGACTTCGCGGTGGGGGCATATGCGACAT
TTGGCATAACGAAAACGGGATTTGTGGATGAGGAAGACGAACCCACAGAAATTTGGAACATTGAGCACCGAGGTTCCGTTTATTTTGCGGCTGTAGATGAGCGTGAAGACGTGGCACGACCTTTT
CGGCCTGCATTCTAATCTTCGCCTCCCTCTCCCCTATATAAGTGCCTCCCTCCTCCCTTCTGAAATCAAACCATCCCCAAATCCCAGCAGAAGCAGCTCAATCGACCGACTCCTCCTCCTCC
TCCTCCTCCTCTTGTTCTTCTCATTCTAATATAATTATCGCTTTTGGTGTGTACATCTTCTATAATTTGAGAGGATG

MaBAM7 (XP_009397011.1) (Ma04_p18390.1)

TAAATCAGTGAGAGTGAGAATCTTAAATTTTAATCTTGATATTTAGGATATATAGATTGATACTTCTTTATCATTTTATATTTAAAAATTTATTATTCTTCAAATCACTAACTTGATAAAGTGTT
TGAAAGAACGTTTTGATTTATCTATAAGAATGAGATTATGATATAGGAATCAAAAGGATCCCTCACTGCTTTCAAGATTTATGGGAAGTGACGATTCAAAAAAATGGCATGATGAAATAAAAGAT
GAGTTTAAATCAATAGACCAAAATGATGTTTGAGAATTTATTAAATTACATAATAATTATAAAAAAATTTGTTATAAATGAGTTTTTAAAACTAAATATAACTTAAAGGGCAATATCGAATAATT
AATTCCCTAAGGCCTTACATCTTAGAGTATTTAAAAGAATATGTTCTTATGATTAGATTTATGAGTAATTTATAAAAGATATAATGATATATGTTTTAATGGATAATAATTGATATATATATATA
TATATATATATATATATATATATAATGATTAAATATGATTGTCTCAATAAGATAAATTATAAGCATCATTTTAGACTATACTAGAAAGAATAAATAATATTATGGCACATAGAAGGAAGTAAGAT
ATTGATGGCATATGAATGTTATGACTTATATTGATAGTGTAACTTAATATAATATTATTATATTTATTAGTCTTATTGGTCAATTTTATAAAATTTATATGCATATGTAAAATATATTTTTAAG
AGTTTAGAATTTAATTGATTAAAATTATTTATAAATAATCTTTTAATTTATTTATTTTGATTTTTGATTTTTTATGTCTTATTAAACCAAGTGAAAGAACGTTAGATTATATGATTCTTTTTAAT
TATGTAAGATATATTATAGATTTGATTGAATTTTAATTATGATTATAAGCTAATAGAAAGAAAGTCAAATTATGATATAATTCCTTATAAGATGACCGAGGGGCTCTGATACTTATTTATTCTAA
ATTCTTTACTTTCACTTATAAATAAATAGGACCTATTATATATTAAATATTATAATATCATGTCCCCTTAGTCCTTATTTATTATTTATAGAAAGAAGTCTAATTGTACAAAGAATAGAAAGAAA
TGAGTCTAATTCTTCTTATAAATCAAAATTATTATTTCTTTCCAATCCGACGATCTACAAATAAGATATTCTGAATAATATCAAAAAGTACGTAATACTTATATTGATCTATCATTATTTGGTTT
AGATTTTGATATTAAAATTTTTATATAATAAAAATATGATTATGCTTACGTGTCACTTTTTATATCCAACCATGTGATAAATGTGAGGCAGAAAAGCTACTCATGAGTTCCCTCCGATCACTTTT
TTTCTACTTTTACATTGAAGATATTGAGCCCACCACATTTGTCTATTTCAAAGATTCAAACCTTCTTGGTTGATCTTTTTCTTTTATAAATCAGTAGCCTCTCTCAACATTATATCCTCAAACCT
GTCTTCCTTCTCTCTCTCTCTCTCTCTCTCTCGTCTTAACAACATAATTCTGCACAGGATATAGTTAATTTTCGAGCTTTCACGTTTTGGCTGCAGCTATG

MaBAM8 (XP_009403535.1) (Ma06_p07470.1)

CTTGTTACACGACCACCAGCCACTTCCGCAGTGACTCGTAAGCCCTGCAGAATAAGAGTGAACAGTGTCGTCTTGTTGCGATGGTGTGACTTGCTTTGTATCTTTGAAGTTCGAAGCTCATTTCC
TTGTGTATTGCATCGAGTCTTTTTAGTATCTCCACTGTTTTCTTCTTTGAAGTCAATGTTTTATCAAGTAGGTTTGCATGTTCTTCATGAAGGAAGTGTCCACTTTAGATGAACATAGCAGATCT
AAACCAGACCAGTTTCGCCCGATCCAATCGGGTCTGAGTTGATCCGATTGAACCTAACTCACGCACGAATCTGCAATTCCTGTCGGGTTTTTGGGTGGGGTTGGATCATATCAAGAAACGTGTGC
GTTCAAAATTCGAGATCGGGTGCGTCGGGTTCTCGGTAGGATTAGACCAGATCAAATATTATGCGGTCAAAGTTCACGTTGGGTTCAATGTAGGTCGAGCTTGGAGCCCATGTCGTAGTTCTGAA
TCCAAATCCTTCGTTCTCCAGCCTTGGCTAGTCTTTCCTTCTTCCTCCTTCTCTCAACTCATAGTGATACATATGATAGTACAATCAACAATGAGAAGACATACTTATCGATAATTTGGGTTTAA
GAAATATTTTGTCCATGAATATTTCATTGAATATTCTCTTTTATTAACCCACTATAAAAAGATACCTAAATATGATAATCAAGATTAAATTTGTTTTATTCTCCTCGAGATTGACTTGAGTATCA
GAAAGATCAAGTCGAAAAACCTATCTAACCTCAATCTTTGTCGAGGTCAATCATGATCGTTCCTTGGCCACTTTAACGATCAACTCAAATCGTGTCAAACAATTTATTTAAAAAAAAAATCTTCA
TCTCACTGTCATGTATTATGAACATGATTTTTAAGTTGTGAGCAAATAATAATGTGTTCTTTTATATGATGGAAGAAACAACGTAGATACATGACAAAACAACGCTATGGATAAAGAGAATTAAG
AGAGTGGCCATCCCCATTTTTCTCATACTTATCTCATCGTTCTTCAACCTAAAGACTATTTTAGAGAAGCCTCATTTTAACTATGATTTGTACTGATCTGTCACTTCAGAACTGCATGAGAGAGA
GAACGACTTGTTTGGAAGTCCATCAACCGGTGGTCTTCCAAATGTCACTCTCAACCCACTGACTGCTGAAGAAGATTTCTGAGGGACATTCTTCTAGACACCTCTCCGTCAAATTTGATCTCTTA
TGTTTGATGCTTTCTCTGAAGAACTTATATTTGCTTACAAATACAATGAAACACAATGTGTTTTGACTCATTTGAGATGAGCTCGGATTGACCAACTACATCTGGGATAATTGAAGAACAGTTT
TAGATGCATGCAAGCAATAACATGGACTCCTATATCACCAGTAACAGGCAGTTCAGTCCTTGTAAATGGAGAAGAAGAAGAAGAAGAAGAAACTTCCATTTTGAGTACTGGACACTGCATCAGCC
GAACTGTGGATCTCAAAGCCACACTGTCGTCTTATGAAGCAGAGCAACGCGGTTGACTCCCCTTATCGACCGATGTGGTGACCATGGGGTGCTTTCCCAGCTTGGTGGAGTAACGTAGGAGACGA
CGTGCAAGCGAGGATTTCCTGTCCTAATGTGCCCAAAGTTCAGGGTTGGACCGCTGACAATGCGACTAATATTCTGACACGTATAACATCAAATAAGTATTTTATTTTCCTCGTTGCGAAGGACC
CTGTTCCCTGTCTGTGCGTGACAGAGAACAATTTAACTGGACCAATGTAAACGCTGTATGGCGAGTGCCACGTGGATTTTTTAAATTAACCAATAAAACTTTTTAAAGAGAAAAAAATAAGACAA
AAAAGGTGGCTATAAATGTCTCTCCGTCAATGAGGACAACAGAAATGTGAAGTTGGAGAGAGATAGGGAGAGAGAGAGAGAGAGAGGAGGGCGAAAAGAACTCTTGCCAGATCATTGCTCCTCTC
CCTCGAGAACCAACTCGAATG

MaBAM10 (XP_009409087.1) (Ma07_p10880.1)

GGGTGATCTGACTTTGGAACGAAGAGGTTGGATCGAGGAACGAGGGTCTCCCTACTGCAGGGTCATCTCATAGCGATAGGGTCGTCTTTTAGTTATCGCTAGTCCTACATAATAGGTCTATATTA
GGGGCTTCTCGGCTCGATCTCTCAATTGTTTAAGCCAATAAGAGAATAGAGAGAGTATTTGTCTGCTTCTTGAGCTTGATTTAGGGTATGACTTTTATACCTATGAGCCGAGAAGTCTAGTCATG
TATTAGCCAAGGCCCCCTTACTCTAAACGACAAATGCATTAATGAGGATAGTTTGCTGTCAGTCATTAATATAGTGGCATAGCTCAAGATATCAGCTAAGAGGTATCAAGTTGAGGCTTTTTGTT
ATTAATGTTCGCTGGTTTTATATCTTTATTGTTTATTAGTCCACCTAATGGAGAGAGCTATAAAGTATCAATATTATCATCCTTATCATTCAGGAAAGTTGAGGTATGCTATCATTAATGATCAC
CGATTTACGTGCCTTTATTATTTGTTAATCCAGGAGAGAGCTCAAGGGTATTAATATTATCCCTATCAACCATCCTCTCCAAAAGCCTAAGGGACAAAGGGCCTTGGTTGCTCAATCAACTCCCT
CTAGATATTCGTCTTCAATTTTATAAGATAGATTAGTAATTAAAATATATTAAAAATGAGATTAGATAAGGAAAGTAACCGGGGATCAATCCTTACTTATAGTCTCCCTTTATTAGAAGTATTAT
GGAGGAATCGATCCCTTAGACGTTTCGAATGCCAACATTAAGTTATACGAAGAAAGAAGCTCAAATCCTTCTAAATATATAGTTATATTTACTTCACAAATGATGTCGTCTGGGACAACATACTC
TCTTATTTGCTAAGCATTCTCTGAGAGATGATAAAAGATTGATTCTTTAAATTACCCTTAGAGTCAGGTTGTCAACCCAAGCTCATATATTATAATTAATACTTGCATTCATCACTTTTAAAAGG
TTTTTATACTTGGTCACCTCCAACTATATTCCCATAGTTACTATAAATTTTCCCAATGTCAATAAACGAATTTAGACACCTAACATTAAAAAGGCAACCAACTAGGAAGTCGACTTATAAGTATT
TCCTTTCATGCCATGCCATGCCATGCCATGCCATGCCAATTCTGACGCTTCTCGCATCGACTGGAATGTGTTTTGAAAAACTCATGCTACAACACTGCACGTTCTTTGTTTTTGCTGCAAGCTTC
TACACAGGAGATGAGGAGGTGTTACTAAGGGTGTTGTAAGTCTACTAGAAAACAGATTGGGTTTTCGATTTTAGTTTTGTGAAACATGGCACGAGGACGACAGGCTCAACAAAGGAGATGGTAGA
AATGCTAAATGCTGTCGTCGTCTTCTGAGTTAATAGAAAGCCTTTTTCTGTTTCGGGAAAAAATAAAATAAAAATAAAAATAAAAAGGCCCAATAAATTGGCCTCCATTAAAAAGGAGACGAAGC
TCAAGAAGAGGCCAAAACAAACCTCACCAAATTACTTGTCCTCCCAAGACCCCTCCCCAGCGACTCAAGAACCCGCTCGAATG

MaAMY2B (XP_009411354.1) (Ma04_p22000.1)

CGTTTCCATGAGTGCCCGGTGATTAGGAGAATATTATTCTTCTTTTTTTTAAAAGCTACACGATGATTAGGGGAATTTTTCTTTTTTTCCATGTGCGCCAGGTGATTAGGAGAATTATTCTTTC
TTTTTTTTTAACAAGATATACGGTGATTAGAGAAATTTTTCTTCCTTTCCATGAGCGCAGGGAATTATTCTTCCTTTATTTTGAAAAGCTACGCGGTGATTATGAGAATTTCTTCCTTTTCCATG
AGCGCCGCTGCTTCGATTCCTCGACCCGCGGTCGAATTCGACCGGGACCATACGAAATCTCCGGTCAGAAAAGCGGGACCATTCGACCGGGACCATACGTAGGTCCCGCTTTTCTGACCAAAGAA
GAGCTCGCGAAGAGTGGATGCTGTCTCGGCCCAGACGCGAAGGGCACGAAAAGCAGAGCGCGAGGGGGGCAAAAGAACAAACGCCTACCGGGCGCACAGGAAACCGATAAAGTTCTCCAAGGACG
AAGACGGAAAGAGACGTGGGAGGTTTGATGTTTTCAGGGGAAAACAAAGGGGAGAAACGATCGCTTACGTCGCTCCTCGACGTCACCTCTCTCTCTCTCGCTTACGTCGCTCCTCGACGTCACCT
CTCTCTCTCTCTCTCTCTCTTGTCTTTTCATCATATTCGAATTCAGTGGCTTGATGGTTCACAGCCGAGCGAGAAGAAGAAACGAGAGATATCGGAATCGGATCCATTTCACGCATTAAAATCGT
TTTCTGCTGTCACGGACAATGATTCCCGAGCTGGAATTCGTCGTCCTTCACGCCTATAAAACGCTCGAGACAAGGCGACGGAGAGATCACAAGACGGCACACAGCGAGCCCTTGATTTGAATTGT
TCTTAGGTTTGTTTCGCCTCCAGCGTCTTTGAGATGATGATTGAGTCGTTGCACCAAAAAGAAGTTCCTTGTTTTGTCTTTTTGTTGGCTAAATCTTTTGCATCTTTAGTTAGCTGTAGGTGCAT
TATATGGTCGTGGAGGCGATTGCTGGGAAACGTTTAGCTAAAACCCGTGAGACTCATTGCTTGGTATTGAGAAACGACATGCACTCTTTTAGCCCACAATACTGGAAAGAGTGCCTCAAATTCGA
GGATTGTTTACATGGGCGTTTCAAGGTTTAAATGAGCTTATTCTAACCATAATTGTATGTGGTAAAAGTAAGGTTTGCCGTGCTAGGATCGTCATACCTATTATTTGAAATATCTGGCTGTGATA
TTTGCTATTTCATATTGCAAATTACTTAATGTTGTAAAGCAGAGTTCTGGAAGATGTTTTAGGAAAACTTAAATGCTATAGGAATATGTTGATTTTTGTTGCAATTTATGATATTGTTCTTCTAA

TTACTATTCTTCTTTTTCCAATCATTTCACTGTTGAAGTTTTATTCTGAGCTCAATGCATTACATTTATAAATGGCTGTCACTGCAGGGATAGAACCTCTCTGCATCTGCTTAACTGAATCAGAG
GTCACAGTTATCTGATAATCATCTAGTTATCAGTTATG

MaAMY2C (XP_009411975.1) (Ma04_p22020.1)
CATCTAAACGATCCATCGCGTATGAAAATATATTCCCTTACCATATGTCAACATGAATCTTTAATTGACTATATAGCATAATAATAATAATAATAATAATAATAAATAGTAGTCCAATGGAATTG
CCGACGGGATTGCCGACGTGCTTTGAAAAGGAGTACAATGGAATCGATGCCGTCGCGACGGTGCATGTAAGCTGTCAGCGGGGGAATGATTTGATCACGCATAGCCGAGATTGAGCTGTTCAAAT
GCGGCAGCCGTTTATGAACCATACGACACAAAAGCTACGCGGTGATTAGGGGCCATATGTAACATCCGGTTTGGATCGATGTGGATCCCGCTTTTCCAACCAAAGAAGAGCTCGCGAAGAGTGG
ATGCTGTCTGGGCCGGTCCTCGAAGTGGTGATGGGTCGACAATTTCTTATGTTATCGTTGTGAAGAAAAGGCCGGAGAGAGGTTGGGGCACAAAGCAGACGGCGACACGAACCGTGTCGCCAGCC
ATCTGCGCGTGCACGCCTCTTACTCTTCCGCCTTCACTTTGTCGACCCCGCGCGTGGGCCAAGATTCTGACGCACTCGTTGGTCGATTCCCAGCCGTTGGTGCCGAGAAGCGAAGGGAAAGACGA
TCAGAGCGCGAGGGGCGCACAAAGAACAAACGCCTACCGGGCGTACAGGGAACCCGATAAAGTTCTCCGAGGACGAAGACGGAAAGAGACGTGGGAGGTTTGTTTATGTAATGGTTTTAGGGAAA
TAGAAAGGGGAGAAACGATCGCGTACGATGCTCCTCGACATCACCTCTCTCTCTCTCGCTTTTTGTTCTTTTCATCATATTCGAATTCATTGGCCCTTCTTTCTTGATGGTTCACAGCCGAGCGA
CAGGAAAAAGAAAAGAGAGATCCCGGAATCGGATCCATTTCACGCATTAAAATCGTTTTCTGCTGTCACGGACAATGATTCCTGAGCTCGAATTCGTCGTCCTTGACGCCTATAAAACGCTCGAG
ACTAGGCGAGGGAGAGATCACAAAGACGGCACACAGCGAGCCCTTGACTTGAATTGTTCTTAGGTTTGTTTCGCCTCAAGCGTCTTTGAGATGATGATGGAGTCGTTGCACCAAAAAGAAGTTCC
TTGTTTTGTCTTTTCGTTGGCTAAATCTTTTGCATCTTTAGTTAGCTGTAAGTGCATTATATGGTCGTGGAGGCGATTGCTTGGAAACGTTTAGCTAAAACCCGTGAGACTCATTGCTTGGTATT
GAGAAACGACATGCACTCTTTTAGCCCACAATACTGGAAAGAGTGCCTCAAACTTGAGGATTGTTTACATGGGCTTTTCAAGGTTTAAATGAGCTTATTCTAACCATAATTGTATGTGGTAAAAG
TAAGGTTTGCCGTGCTAGGATCGTCATACCTATTATTTGAAATATCTGGCTGTGATATTTGCTATTTCATATTGCAAATTACTTAATGTTGTAAAGCAGAGTTCTGGAAGATGTTTTAGGAAAAC
TTAAATGCTATAGGAATATGTTGATTTTTGTTGCAATTTATGATATTGTTCTTCTAATTACTATTCTTCTTTTGCCAATCATTTCACTGTTGAAGTTTTATTCTGAGCTCAATGCATTACATTTA
TAAATGGCTGTCACTGCAGGGATAGAACCTCTCTGCATCTGCTTAACTGAATCAGAGGTCACAGTTATCTGATAATCATCTAGTTATCAGTTATG

MaAMY3 (XP_009412382.1) (Ma08_p04100. 1)
TGGGTTTGCTTCCGCTGGCCGTCGAATTCTACGACGACGTAGGGGCTGGAGGTGCCGTGGCCGTCCTTGGGGAGGAGGTCACGGGCGTCGATGACCTCGACGGCGAGCTTGCGGATGACCGGAAG
CGGTAGGCTCGTCATGTTCTCATGTGAGGCAGAGGATCTCTTGCTTCTTGTTCTCTGATGAGGGAGGAGCAAAGGCGAAAGGAGAGGCGAGGATGCTCTCTCCTTTTGCTTTTGCTGCTGGATTC
TTCTACCATGGATGGTTGGGTTGCTTGACATCGACGCTCCTCTCCATCCTGCTCTTGATTCCTTTCTTGCTTGTCGTTCTCCTTCTGCGTTAGCCGTTGGGTTGCTTCCCGCCCTTTTCCAAAGT
GGATTCGGGACATAGAGAAGTCAATCTAATCCTCAACCAGTAAGGCAGGCAGCTGACCATGTCGACGCAGCGTTCATCGACTTCTCATGAGCTCTATACATTGGCGGTAGGTAATCTCGATGAAA
ACTGTCGCTGGTGGTGTGGTGGCGGTGGAAGACGGAATCTGTTCTCCCGCACTTTTATGATCATGTTTCCAGCCACTTGTGTTCCTCAAGCTGAGAGGAACAAAGGAATAAAGGCCACGATGGAT
GATTTGCCGCAGCAGCTTCTTGTGCTTCAAACGGTGGTCATTTGCTTCACGCCTTTTGGATCTCAGCCCATCGATAAGTCGACCAACATTTTTGGGCCAAGCTCAGATTGGTTGCCAACAATTAG
ATCCATCGCACAGCCCAATCGTCGAGCAACATCGGGCCATCCTCAACACAAGTCACTCACTACATGACATCAGCCTATCGCTCTCCCTTCATCCTTTTCCTCGCCCACCTTTTCACGGAACAAGC
AAGTGGATCAGGTGGGCCAGTAGAGAGGACAGAGGCTTTGAGCCTATACGTTGCCAACTGGATTAGACATGTGAGGGGGTCCCAATTACACATGACAGGTCCTATCCAAATCCACTCCTCCATT
TTTGTAGATCTCTTTCCACATGAAAGGATCGGCATTAATATCCAAAAAGATAAGAATGAATTCGTCTTCGCCTTTTTGACCTCTGTTTCTTCTCTTCATTGCCGATCCGGGCGTCCCTATCCTTT
TCCAATGGTCACACTGGGAAATGCTGCTACATTTATCTTAGTTCTTATCATTATTGGAAGCATGTGTCTTGCATCCCTATCCCCCGTAGACGAATGAATCATTGTCTGAATCATCAATAGAAACC
ATCTATTTTATGGGAGATAGAGAAACTCCCGCGGTGGAATCACGGTGTCCCCGTTGGTTTCGCCACTGTGCTCCAGTTAGAAGCGGATCAAACTTTTCCTTATCCACGTGAAATGTGTCGTCGGT
CGCCCGAGTCATGTCGAACGATTTCTTCGCTTGCGGACTCACGCCACACCACAGCCGCCCCTGACGCAATTAGCGTCGAACTCTTCCACGTAACCGCCGCTTTTGGCGGTACGTCGCAAGATCAT
ACCCCCGCGAGCGCCGAGGGACTGTCGGATCCCGTTCGAGAACGCGTGTAGCCAGTGCTTCGATCCTCCAATGAATAGCCTCCGATCCGCCACGTGGGTCGTTTTTGAAACCTCCATCGAACCA
GCTGATGTAGCAGATCCCGCTTCAGGTTGGCTATTAAAGGAGCCATGGCTCCGTATCTTCTCCACGTAATTGAATGAGGAGTGGAGGATATCCCCAACGCTGCTTTCATAGAGGCATCTTTTCCT
GGTCGGTGTTGCGCTTCTTCTCCTGAAAAATCTCTTCGCTTCCTTCTCCTCCGTCTCTCGATTCTCCCCCACCGTTCGCCGATG

MaAMY3A (XP_009399983.1) (Ma05_p08000.1)
GAATTGGCATCTATCAGTGCCAAAGGCCTTTGGTTTCGGGGGATTATTATGCCATGTTACTGGAGCTGGTGGTTCTCACTGATGCTTTTCAGTTAAGAATATGAGGGTCCGTGAAAAGGATATGC
ATGTGATGCACATGTGGAGTGGAATCATTTGTAAAGCAAGAAATGACTAAGAGCAATTCATATCTTATCAATGTTACTATGTGATGCAAATAGTCGTTGATTTTTGGCCCACAAATCATCTCTT
GTGTCGACCAAAAGAAAAATAACTCATATCTTTACGTCACGTAGAGATGCATGCATGCATTTGAAATTTGGCTTTCAGATGCATACATTCAATTCTAACACTCATTTGGCTTTCAGATGCATTC
AATTCTAACACTCAAAAGAAGAGACTATGATTAATCATACCAATTGTAAACCAAAAAAAAGGAAATACTCTGATTATTGCGAAGAATTTGTATTTATTCGAAATTATTTTAAGAGGAGATGTTAG
TGTATTGTTGGAATAAGCTAACACTAATAATCTTCATTAACGAATCAAAAGGGAAGCTTTCCGTAAGTACGTACTACTACTCAACCATGCATGCATGCATAAGGCCGATTGATACCATGCAATAT
ACTAATCGTGTGACTTGAGAGAAAAAAAGGGGAGGGATTAAATAACATGGTTTATGTCTCCTCTCAGATGGCCTCATCGACGCCAATGATCAAAGTAATAATGCATCTCAATATCACGGTCGATT
TCTCCATTATTGACTGCACCAACACTCAAACATTATGAATCTTATCTATACCTCTCTCTTACAACTCATGCTTTATTGTATATACACAACTTAATGTGTCTCAGACCCAATTTCCCAGTCAAGCT
TCTCTATTTCTCTAAAGAAAGTACTCATCTCAGAATCAATAGGATGGGGCGGACAAATCACAAGATGAAAAGGAGTTCCCAGTCAAGCTTCTCGACTGCACATTATTTTGTCATGAAACCATGGA
AAGGGACAGGAGAAACGTGCATGTAACAACATCTGAGAGGAGCACAAAATAATCACATCAAAGTGGAGGGAACGGGCGCCACCCAGCCCGTTGACCCATGAAACCAAGCTGGTTTTGAAGGCAAT
CATTGAAGAACTGAGTCGAACGAGGAGTAGGAACTCGATGTTCCTCCCATCAAATGTATGGAAGTGGAACGTCAGTGGTGTGGTTGTTGACTAGAACGAGAAAGAGGAGAAGCTTAAAAGAGATA
GTTAAGAGCGTTTCTTTTAATTATGCATTTAGCTAGATCCAAACAGAAGAGCGGATCAGTTCATGTCTGCTGGGCCTGTCGATCTCGATCCGTAATCATCGAATGAAATGATCCGACCCGTTTAA
TTTGACTGGCGGAATTATGGGTCGAGTATACAGACCCGCTAGCAAGTCGGGTCGGATCTAAACACAACTTTAAACGTTTTAGGGGGGCCGAGGCAGTGAATAGGGTTTCTGTGAAATATTTAGCT
TGTTTGGGGATCTAAACACAACTTTTAACGATTAGTTGGTCTTCATACTTGTGTCATTATCCTAAATTAGGTTTGCTGTGAAATATTTAGCTTGTTTGGGGAAACAATCAAAGATCATCATGCGA
GGCTGTTTCTCGTTTGAATTGCGTGTGCTTGGTTTGATATGTTTTTGTGGTGTATGGTGACTTGTTATTTTGTTTCTTTTCATGTAGATATCTGTTTATAATTCGTTTGCATCCTCTTTTTTTTC
AGATCTGAAAGTGAAATCACTTGGTCCTTGGATG

MaAMY3C (XP_009381896.1) (Ma10_p30040.1)
CGTTGACTGAGTTGTGCTACCCAAGCATTTATAATCCACCCATTTGGTATAATAGTCAATGATGACCGGAACTCGTTGACGTGTGAGATTTCCGTGTGTCAGTAAATTGGATCATCAAGCCACCG
TTCTCAAAGGCTCGCTCGATCCAAAACTATAGTCGCCATTGTGCTGTTGGCCTTCTTCATGAGACTAACAATCGGTACTACTCATCTATCATGATACATGAAATCAATTAGGGATGGATTGCCTG
AAGGAAGGAAGCCCACTACTGACTGAACTCGAGTCTCGGCTTGGTGCCACGATCTTCTGCCACTGGTTCTTAATTGGTCGTAATGTGTTCTTGGTGAGTGAGACCCGTAAAACGGGGAGTTGAGC
ATGCAGGGAATGGTGGAAGAACTTCCTCCAACAGTTGCTTGAAAGGCTTCTGCATTAAATCCATAATCTATCCGAGTAACAATTTGTCTTCTCGTGATGTAACGAATGACTCCATTTTTTTTCGC
GTAGATTCCTTTTTTGGCTATGCACAAATAAATTATGGTGCCATGTTTATTTGTTTTTTTACAGGATAAATAATAAAGACGAAATTCGAATTCAGAATTTTTTTTGTGATAAATTGTTAAGATCA
ACTAAGTTAGCTGAAAATTTCTATTGATTAATGAACGATGTCACAGCTCACACTTCAACTGAGTGTAATATGATTGCATCGGTCAAAATTGCTTAGAGTTCATACAATCGAGTGAGTGTAGTAAC
TATGACATCTTATTAAGGTCTGTGCGTTCCTGTTCTTTAATACTTAAAATAACAATTTATAACGATATTATTTTTCCAAGATAATTACCCTAATTAAATATCTCAAATTCACACAATCAAATATA
ATATCTCAAATTCACATAATCAAATATAATTATAATAATTGTGGTTTTTTTAATTGGTACCCTCGATTTTATTGTTTTCAAATAATTACCCTGATTAAAAAAATAGTCATATTGCCCCTCAAAAT
TTTATTTTGACTTATTACAATGTTTTGATCATCACAATAAAAATATTATAAAGCTTACGTGAAAATAATATAAATAAGCTAAATAAAATCAAAACATAATAAAAATAATTTGATATATCAAACTA
AGATTCAAATATGCATTTATAATATTTTATGAACAACATCCTCTAAATAGAAATAAAAAATTAGATTATTATAATGTTTTGACCATCATAATAAAAAAATTATAAGATGCACACGAAAACATAGT
AAATAATTTAAATGGACTCGTAATCTCAACTAAACCAATTACAAGCTTAAAAATATGATGCTTATTATTAATGTCTACGAACAGTGATGGTGAAAAATAAAGTTAAATGATATTATAATATCACT
TATAATAGTCTATGATATTTTCAATAAATGCCTATAAACACCATTGAAATTCACTATAAATAAGTTAAATGAAAATTTTATTATAACAATTTAAAATTATTAATAAATAAATAAAAATTTTGGTG
AAATTTTTTTTTAGATGTATTTTGGATATTTTTTAAATTAGGGGTATTGTTTCAAAAAAAAAAAAAAGCAAAAGATGGGATACCTTTTGGGCAATATCCAAAATCTGAATATTTTCTAGAAAATTA
TTCTTATAACATCTCATTCAAATATTTTATTATTATTATTTATTTATAGATTATAATTATGTATTGGGTTCTTAGGATGAATCAATATAATATATCCTCGTCTATAAGGATATGTTTGTCCGATC
GTAATGATACTTTACATAAAAACACGTCCGGATAAATCGTCCGCAAATGTTGAGGTACGACTGCCATTACTGCAGCCTCCATTTGAGCCTTCCACAGACACGTCTGTTTCCGCCCCCTTCTCCG

GGAAACTGAAACGCAATAAAGCAGGCGACGTCTATCCACAACTGTAACTGAGACGCTGCGGCTGCAAAAGTCTGTGTCACGTTGCCAACTGCCTTCTCCCCTTATCCTCGCTTCGGATTACCAT
TGGCGCTGGCTTCGATGATAGGGACTCGACCACTAAGACCCGACCAACCATGTATATAATGAATGGCCTTAGCTTTCTCATCGTAACCCAATCCGTCCCATTTTGTTCTGAAGAGTTTAGACAAG
GAGAAGATG

MaISA2 (XP_009404709.1) (Ma06_p17710.1)

CAAGCATGGAACCAAAGAACTTAAGTTAATATAAGAAAGTATATATCTGAAAGTGAATCTTTAAGACTCTGACATGATCCCCACATCTTAGGTTCTTCAAAGATGGCTTAAATTAAATAGGAATG
TTGCTTAAATTAAATAGGAATGTTGCTTAAATTTAGTCTAAATATCTTGAGGAAATTTGAAAGTTTAACCAAGGAAGAAAATCATCTTTGTCTCTTAAAAAAGGGATAAGGTGGCCTATGTAAGG
CCATCTGGAAATCACTTCTCCACTGATCTCTCACTATCGTTATATCGTCTTTGCACATTGCATTAAATCTCAGAGAATAATAAGTATGATACAGAAGCTACATGCGCGCCAATTCATCAAAGCTT
GATGTTTTATGAGATGGCTAAATTTCAGGCAGCCTACACTTTCGACGCGGGGCCCAACGCCGTCTTGATTGCACCAAACAGAAAATCCGCTGGTCTTCTGCTTCAGCGCCTGCTATTCTGCTTCC
CGCCACCAGCAGACAATGAATTGACTAGGTCACTGGGTTTTCAGGTTCTACAACACCGATACATTGGTTTGATCTGATGTCAAATTTAATCGCGTATCTTCCTTGCAGTTATGTAATAGGAGACA
AATCAATTCTGCATGAAGCTGGTCTACAGTCCATGAAAGATGTTGAAGCCCTGCCACCACCACCTGTGAGCAAGGACAAGTATCCATCTCAAAAGTTCCCCGGCAAGGTCAGCTATTTCATCTG
CACCAGGCTTGGGAGTGGACCAAGGGTGCTCACTGATGAAAGTCTAGCTCTGCTAAGTCCCACTACTGGACTCCCAAAGTGAGTTTGCTTACAAGAGAACATGATGGTCATTGGAACTACTTTGT
GAGAGATGCTCCCTTTATAATTCTTGGGTTTTGCCGGAATATCTTAGTATGATCCTTTTTGGATCGTTCCTCTTCTCTCTTTTCGCTTTGGGAAGAATCTATGCTTTTATTTTATGTGGGGATTG
ACGGACTTCTTGCACTAGCTATTTCTCTGCATATGGTGAGGTCAAGATATAAACAAGTTGCTCAGGTTTTGCTTTCGATTCTTGCCAAGATTGCTATTTATTGTTGTTGAGAATGTCTATGATGG
CAAACGTAATCCAACTTGAATCGTATTTTCATTGGGAAGAAATATATAAGCCGTTGGTATATGATATATTATGCTATCTACTCGTGGCGGGTCCTGTGCGATCGAGAGTTGGGCGCGGACAGGGA
ACAGGGAATTAGATATGAGTGTAGCCCTCTGCTGGTCCGTGGTGTTCCAGTGAGCCGATGGAAAAGGCTCCCTGTCATGAGTCAGACACGTGATCCTCGACCTTGTGGGTCCCACCGACGAAAC
GACCACCTTTCTTCGTTCACGTGATTCCCAGGTGCGTTGTCTTCCTCCTTGGAATCATCCCCATCCTCTTCTTAAATCAAAAAAATCCAGATTCTTATTTTGTGACCGAGAGAGCAAAACAGTC
TCCGTCAAACATATCCCTCCTCCTCCTGCTTTGTTGCCTTCACCATCTCCCGTCCCTCAGACCGTTTCGCTTCGCCTCCATCTCCGTCCTCCGAAAGAGCCACGTAAAACCCCTTCCCCTCACCC
TCCCCTCCACCCAATGCTTTCCCTTATTGATTAATCCCGAATCAGACTCATTTCTCACAGCAGGAGGCCTGAAAGGCTGCTCTATG

MaISA3 (XP_009417596.1) (Ma09_p14380.2)

GAGGCATGGATCCGAGGCTTGCAGAACATCGTTTTGGGTTTCAATGGCGTCGCCTGTGTGGGCATGGCCACATCCTTCCCCCTTGTCACAACGACTACTCCGGCTACGTGATGCCACGGACTTCT
TTGTCGGGGATTGTTGCATGAAGTGCTTCTTGACGTGATCTGATCTGATGACCACAGTGATGACTGAGTTCTTCATGGCCGAGGGGAAAGGCGACAAGGACAAGTGGAATCCATGAGCTCATGG
CAGGTGTTTGATAGGTGCTGCCACTGATTCCACAGTAAAGATACTTCTCATGCTTGCATTGTTACTGTGTGCAAAGGAAATAATCAACATGGACGTGTCCCAACTTTGTGGTTCGTCTGCTACTA
AACTTATCAGGAACAAGAGGAGGAAGAAGAATCACAATAATTAATACAGGATTCTGACATGAAGAGGTTTAAGCCGAAGACCTTCCTTCTTACTGCTCAGCAGTAGTCTTAGAGGTTTGAGTCGA
GAGTAACAGAGGTAGTGAACCGTGAATGCTACCATGAGTGTTGAGCTACTTAAACTACGTAGGGTGAAGGTGATGTGCATCATGGTTCATTGATTACTGAGCATTACTTGTTTGTACCATATAAA
TGCATCTATATTTTATGGATCGATGGTTCTAGTCGTGTCTATTGCATGAGATAGAAAACGACGTCATGACGCTGAATGAACTCTGGAAGCCATGTTTATATTTCTAAGGTCCTCTAGAAATTACA
TGGAAGGTAGGAACAACAATGTCCTCTCACAATGCTGGAGACCAGCTTCCAAATTACATCCAAAACAATTACTCTCCCTCTCTCTCTCTTAATCCCTATCTACGGGTTTAGGAAAGTGATAACCT
GTAGCACGTGCTTGGCACGTGGGAGGAGTGGGACCCACGGACGCACTGGGAAGAATCATTCACCTTCCGATCACGTACGCGCGAGTCGAGACAGGTGGCCGCTCTCGCTGCTCTTTCATTGGTC
AAATTTCACAGGGCCCCGCATTTCCCCATCCTTCACACTCGGGTAAGAACTGACTCCCGTTCACCCCCTGACGTCCGCAATCTTGATTGGTTCCCCTTATTGGGACCCACACGTCCCGAAGCGTT
CACGAGACAAATGCCATGTTCTTTTAGAGAGAATTGCGTTTCACACGGAAATATTATACACACAAATGTACCCACATGATCATTACTGTAGGAATTTGCTGCCACTGAGATGGGTTTCTTTTCT
ATTGGTGTCTGAGCCACCGCCCGGTTCACGATAAACACAACCACATGAAGATGACAACGGAGATAACAGAAAGCAGGGAGGGGAGTCGTTGCGGGCCGCACATGTGTCGAGTTCCCCACATCCC
GAGTCCGCTCACGGCGCAGGTGAAGTCGCTCATCTCTCAACGAAGACGAAAGATCACTATTAAGTGGCATTTTCGGTGATCGGAAGCCACCGTCTCTCTCCCCTTTCATCTTCTTTTTGTTTCCT
TCGATCTCTCAATGCAGTGATCGGAATCCACCGTCTCTCTCCCTTTTTATCTTCTTTTTGTTTCCTTCGATCTCTCAAATG

MaPHS2 (XP_009407534.1) (Ma06_p35790.1)

CGTCATCAAGTTGGCCTTGTGTTTATATCATCCTTACATCGTTCTTAAGTCGATCACAGCTTTGTGTCGACTGCACCGTTCCTTCGAGTCAGCCGAAGAAGCTCGACTTGATTGAGTGGGATGCT
ACCTGACCTTTTCATGTTGGGAGTACGTCGGTTGTTGCAAGAAGATGACTGTAATTGGATGCCATTGAAACACAACCTGATGGTAAAATCTTTTCCTTATGGAGTTTTCCATCTCTATTACCAGA
CCGTACATGCTTTGACGTCAATATTAATAATTAAACTATAATAAGCGTTGACGGTCATGATCACACAAATTTGACCAGAGCCCGATCCACTTTGGTCGGGTCAACCTACGCCTAATCAAACCCGA
CCTATCTTGTTCGGGTCAGCCCATGACTGATTAAAATGCAGCCTACCATGATCAGGTCAGCCCAAACCCGACTCAAGCTTAACCGGACACGCACGATGTATCTATTCGCTTCCTCGGATACAATA
TATCCACTCAAGACATCAGCATATATAATCACACACGATCAATATGACAACTCTAATTGCAACGAACGGTTCATATCTCAAGCCGTTCAAGTCACGGTCCCCGTGTCAACTACCACCAGAAATAG
CATTAGGTCCAATTTTTCACGTTTTGAGCATCCAAAATATCCTATTGTTCTTTTTTACCCTTTATCGGGAGGAAAAGAAAAATAAATATCTGATTCACTCGTTCACTCCTTGATTGAGAATAACA
AAAGTTCCGTATTTTCTTTTTATGATTAGAGAAACTTCGATATTTTCATCATCCTTTTCCCCCCCCCCCCACCCCTCATTACTTCTCGATATATTTTTTTCTTTTATCGAAGGCTTCTGCCCCT
TCACAATACGCCAAAATCCGCCACTTTTGTCCGCTTCTCTCTGAAACCGTTCGTTCCCGGGACCCCACGTCTTTCGCCTTTTCATCGAGTCACGGACGCTGCACAAAAAATTCTCGCTTCCATCT
CTTTTTTCTCCTCCTCGGGAAGCATTCTTTTTCCCCCTTCAATGCATTCTCATCTTGGATCCCCGGCTTCCCGCACGTGACTCACCGCCTAGGATTAGTAGGCTCTTTTGATTGAAGGCCGCGCC
TTCGCGAAGAGAGGCGAACCCCATTCTCGAGTTTTTGTGGCCTCACTCGGCTGTCGTACACAGTGGTTACTTCTCTTCCAACTCAATTCTGTTTTCCTCTTCTTTTTTTCTCTCAGTTCGCGTGG
TATGATTTTGATTGTTTATAATCCATTCCAACTTCATTGTGTTTTCTGATGATTCGAGTTCTTTATATCGTAGCCGGCCATTTCCTTCTTTCCTTTTTCGGGTCTTAATCCGTATTCGAAGCTTT
TGTCGAAAAGCAAAAGACAAGAAATACTTTAGAACAGCTAAAATTATAATTGAGATACATATTGCTGAGCTCACATGTCGTGGTTATGGATTCTTTAGGGAGCTTTCCCATG

MaMEX1 (XP_009400667.1) (Ma01_p08940.1)

AGAGGTCAAGGTGGTGCTTGTGGTCCAGTGCAAATCTCTCTGGTGGAGAATATCTGGAGGTAGCAGCTTCAAGAAACAACAGCAAGTCACATTTAAAGGTGATTTCCTTTTTCAGAACAGCAATT
ATAAAGTTCTAACTCTAATACCCTTAATTAAATCAAAATCATCTAAATATTTATTTCTAGTTTGATACTTTTGGCCTTTAATGATGTCACACTTTGTTCTGTGAAGGAGGAATAGCAGGTGATTC
TTGTTCATAGCTATTACAGATGTTAAGAGCATCAGTTTCCTCTTTTCAAATGGTGAATAATAGAAGCTCTTTTTCTGCTTAATTCTACAACAATTTTAGGGCACTGCCAATTGGAGGCGATCAA
ACTCAGTTCAATGGCTCGATCATTGCTGATAACGGAAGAGGTTGTGGAATGTTGTAATTGGTATATTTTTTTCCCCCACCTTTCAATTACTGAAACAAATCCTTGTGTCAATGACCATGTTAAGC
TTTGAGATAATTTGTTTGTTTTTGTATCTATAAACTGCAGGTGAAGGCTGTGAAGTGAATGACAATTAGCATCTCTCTGCTAAGGTATAATCCCATTCTTGATAATGCTTGGAACTGCCCACTGC
CTCCAAATATGAAAAGAAAACATTAAAAAAATGTATTTTATTTTTCCATACATGAGTATTTTACACTTATAATGAGACTTAAATCATCAATCATATAACCATCCATCCCAAGACAAAATAAAAAG
AAATAAAAATTATATTCCTTCCATAGATAATGATAATGATAATGATAATAATAATATGTGCATGTGGAGGACAAGCTCTTCAATCCTCTCTTGAAACCTCCTAAAGCCGGAACACAAGCTCGGCC
ACCACAAAGGCTCCTCTCAATCTCATCCAATATCCGTCCTGTCACTATAGCAATTTAGGTGAATTAGGGATTCACAAAATGTGCCCCTATTTGAGAATTTTCAAAAGAGCATAACTTGCATTTCA
ATTCTTTATATAGTTTCTTTTCTTATTTTTCTGAAGTTGAAAGGCTATTTCATTATTTTATACATAATTTTGTAATATCCTTTTTATATAAATTATAATATTTTATAGTAAGTTTCAAACTCCAC
TCTGTTCTTAAAAAGATTTTACATACTCAATGACACAAAAAAAAATGTTATTTAAAAATTTTACAATAAATTTGAGCATAAATACTAATGCATACCATAAATGTACCTATATGTGTTGTCTATAA
TGGATTTGTTTGTAGATTGGATCTCCTCCAAGCTTTGCACTGCTCCCATCTCAACCGTTCATCCATTATCTCTTTTCTCCAGGTATGGGTCCACCATGGATGAACAGTTGAGATGGGAATTGTAC
GATTTTATTTACCCTGCATGAATTGGAGAGCATCTGCCACGGAGTAGATTCTCCTCATCCCGTCTCGCTCCTCTCTTTCCCTCGGTTCTCCTCCCCATGCCTCCCTCCCTCCAAAATG

MaMEX2 (XP_009397793.1) (Ma04_p34170.1)

GCGTGGAGTTGATATGGGTGACAATACTTTCAACGTAAGCATCACTGTTCTTACTGTTCTGCTTAACATGTTTAATACCATGTTTCTTCTTCTTGTTAATGGTCATGACATAGCATATATTTTTC
TTGATATTCTAGTTATTCAAATGAGAAGTCTGAAGCAAGAAATTCTGATGCCACATTGGATTCAAATGTCAAATCAGTGTCTGCCGCCAGTGAGGTATTGCAATCTATAGTTTCTTCGAAGCTT
GTTGAAGTCATCATACAGATGGTCAGGGGATACTTCTGAATGCTTTTAATCTTTCCCATATAATATTTGCAGGAATCAACCAAATAATTCATACAAGCTAGCTGTTGGATTGGCATGAAATATCG
CTAATCGGAAATGTAACCGAAGCTGCATCAGGATCATAAAACGCATGTACAAATTGTGTATGTGGTAGGTAAATCGATAGTACAGCCACATGGTGGGAAGATAGTGTTTGCTTTCCATGCTTGTA
ACCGGTGACACTAGTGCCGATTCTTGCATATGTTGCACAAGTTTTGCAATGGGTTCGATTTTTTTTCCAATTATTCTCTATCATTTATTATTTATTCACTTGCATGAATGCCGACCGAAGATGT
TAAAAACCACCTTATTTATTGGCCATATAATCGCATATCTTCATATTTCTGTTTAAAAATCAGAGTGAACCGGCTGATTTATTCACTCCTGGTTTTCGTATAACATCATAAATCAAATAAAATCA

TTTGGTTTACATGATATTCTTAAACTTGCTCAAATGTCATATAATTCGATCTTTCTATTATTTCTTTCTAAAACATTTGTTTCCTTCGATTTTCTTATTCGGTTCTCTCCCTCTTATTTTTCGC
TTGTTGCTCCTCTCCCTTCAACCCTTCTTCATTCTTATTCTGATGGGATCTTCTCCCATTCGGTTCCTCTTTTTTCCTTTCAAGCTTCTTTAAGTACAAGTAAGGTTCTCTTTTTTATCAGAACA
AGAATGTTTCTATCAAAGTCATTCTTCTCCTTCTGACAAGATCTTCCTCCTACAATGTTACTCTTTTTCCTCTCATACTTCTTTTATCTGACAGTAAGACTCTTTTGTTCACAATAAGAAAGAGA
GTGTCAATATATTGATACAAATACAAGTATAGGAGAGATCAAGATATAAAAAATCATAATTCAATTAAGCTTATCATTCTAAATATAGTTATCATGATTGAATTGAAGAACGATTCGATGGAGCC
TAAGATGAATAGATCTGACTGGATAAGTGGATTAATTGGTTGAACCGTATGCTTAATATAATATATATATAATTTAGAATTATATATTAATATAAAACTAAAAAACAATTATTTTTAAACATAAATG
GCCACAAAATATCAATCATACAAAATAAGTTAAAAAGAATATTTTGAAAAATAAATTATATTGATCCAATATAATAGTTAGGAGAAGACCATATTTATATCTATCTATAAACTAGATCTTACTTG
ACTAACCTTATCAAATAGACATACACCGTAGAGATAAGAAGGGCAGAGACTCGAGTAATAAACGCCGTGTTGGGATCCGAGCCCTCGGTAATCCCCTTCTTTGCCTCCCCCCGCTGGTCCGTCGC
CGTTAACATCTTACTTCTGTAACCGTCCATTCCCTTTTCCCACGCGGACCCACCCCACGTCACTCTCCATCCCGTTCCGCGTCACGTGGCTCTTCTCGTGCCGTCGGTCACCCTGCTCTTCTCGT
GCTTGGAGGAAGAAGGCCCCGATGTCCTCCCTGCTTCCCATCTCGTAACTCCTTCCCTCGCCTACCTTCCTCTCTCTCTCTCTCTCTCTAGACTTCTGATTGATG

MapGlcT2-1 (XP_009421446.1) (Ma10_p15040.1)

CAAGATATCCTTAGTACCATGCAGCATATCAGGAACTGTTAGTATGTGTTTGTGCATTTTGTATTTCTTCAACGGCCTTACAATTTCGATTGTTATTTTTGCTGTATGATTTTTCAATTGTTGAT
ACATTTCAGAAATAAAGTGCTAATTGAACCAATTAATTTTTTGATAAAATTATTGTCAAACTATGATTCCATTGTTCTATATTCTCTGATATTATTTCTAATTTTCCTCCATCAGAGAGCTGGAG
CTGTATTTCCTCAAAGATCTGAGAGCTCTGCACTTGTATTTACGCCACAAACATGGCCTCTGAGATCCTATCCTCCGTCAGTACGAAGTCCTGATTATCAAAATGAAACACCTGAATCTTCAAT
AGCATCTGATTTTCCTGCTTTGAGGTGTTTCCCTATTCTCATCAGTTATCTGCAATGAACTTCATCACACCTAAGTTTTGGTTCTGATACAAAATTTATTTAATTTTTCAGTCTCACATAGATTC
AGAATGCCAATGGTATATTTGGATTCTCGGTTCTTAATATTTGGGATAATCAATATAAGCATATTTTAGAATTTTTCATTTCTATTTAACTTTAATTCAAACGTAATGATAAAAAGTTAGTTCAT
GTCCTATCGTACCTAACACTTCTCGTCTTTTTTGACCACTGCAATATAAGTATTTTGATGACTATTTAAAATTTTATTTAATATATATTAAATATTAGTTTGATGGTATAACAGACTAATTTACC
AAAGTCAGAGTTCAAAAGCTCATTTAATATATTTTTATATCTTAAATATTTTTAAATATTAATTTAAATATAATATATTTTTTAAAATATTAAATTATTTTAAATATTAATTATAAATATATTTT
AAATATTTAATTATAATTTTTTATATTTTAAATATTAATTTATATCTTAAAATATTTTAAAATATTTTATATTTTAAATATTAAATTAATTATAAAAAATATATATTAAAGTTATTTATAAAAT
TTTAACAGTTCGAAATTTCACGATATAATCCACAAGGCCTAGAACAAAGGCAACACTAAGAACACCGAACTGGAACAGCGGTGGAGTAGGTTAATTAAACTAGTTATTCACGAGTACCACCGTCC
GCGCCGCTTCCACTATGCGACAAACCACTTTGCCGGCCTTTTACACGCGTCCGGCTGTGGACGCGGCAGGCGACCTCAGTGACGTCGCCTCGTCCACACGCCTCGCGGCGCGCGGCGGACGGCGC
AGGTCACGCTGTCGGTCGCCCACGTTGCCTTACGTTACGCACGCCTTTTAACGCAAGGAAAATAACGGCCGTTACGCCCCTTCTACCTCTCTCCCGCCTTTTCTAGATGTGCGAAGAATACTTGT
AAGCCAGAAACTTGATGCTTAACGGAACGTTATAAACTTTATTATAACGGTTTTGGGGTGTCCGTTACACCGGCCAATAAATAAGCGTACCAGATCTATATGTTGGCTTTAAGGTGTCGCCAAAC
CCTTTCTTTTTTAACCACAAAAGAACGTAGAGGGATCGAAGGCCAATCTGTTACCTCTGTTATCTCTCGAGCTGGATACGATTCGATTCACTTCGATTCATCTGTCTTGTAACGATTGATGCGAT
CTTGATTGCTTGATCTCCAAGAATAAGCGCTGGAGAACGGGGGATCTGAAGCCGATAGGCGATG

MapGlcT2-2 (XP_009413386.1) (Ma08_p19450.1)

CGGTTTATCTCCATGATTCGGATCGATGACCGACTTATGAACTAATAACACTTGAATTTAAATCCTCCAAATTAGATTACGGTAATTTGCAGGGCAATTAAGTCTCATTTTGCTCAGCTACTCGT
CACAAGAACTACTCTTTTTCTTGCTGTCACAAAGAAACACTCAAATTATGAGTACTGTAGATTAGAAAAAAAAAACATCTTTTAATGGCCAAAAATTAGTGATGAATTATGGGCAGGATGATTAA
ACATCATGTATGGTGTTTGACATGTTCAAAGTTTAATGCATCAAAAGAAGCTTGGTCATAACTAATAAAAAAGACCTTATCCCCTTGATTCAGAAGCTTTGCTCGAACCATGTTTCCTAGCATTA
CAATCATTACTCTTTATTTCTGGAACTATGATTACAACAATCTTGAGCCATTTTCTGCATAATTTATTCATCTCTAACAAATAACATAAGTAAAATCTTTTTTTTTTTTTTTTTGCGTTCCACA
TAAGTTGTTAAGGGAACGGAGCCGAGTAGAATGGTAATATTACTCTTTTACAACCTTAGGGATTATTATTTAATTAATACGATTCGTGTGTCTCGGATATATTTTATTTTTTTAAGTTGATGAAA
TAAATGAAATATATAAACAAGAGATTTCAAATAAAAGATCACTTATGTTTTTTGAAACTTAGGCGGTATAATTGTTTGAGTATTTGATGAAGCATTTATATATACATACATTCATATACATATAT
ATACAAGCATTTCACATAAAAATTATTTATGCTTTTCTAAGCCTTATAAGCTTGCATTTTATACGAAGAGTCACCTCAAAGCGACGCCTCTACTCGTGAAACCAATTACTCAACCGATGTTAGTA
GTCCGTTTGAATTCACCCCAATTTGTACAGATGCACTCCTTATCTCTCATGTTGCCAGCAGAGATCAGGGCGGTGCACGAGCGACGGACATGGGGTGAGTTTATCACTCGCTTGAGTTCGTTACC
ACCGCTCACCACTACGCGACCAACTGCTTCCCCGCGCACGAGATCCCACTTTTCCTTACCAACACGTGGCGGACGATAAAGCATCAAGCTACCGCAATGACGTCCGCGGTTGGTCACGTTATGT
TGTGATATTTAACGAACACATGAGATTGAGATCACACCGACGGGACTTGCTGACGTATCTGTGCCGTAAATCAATACTTTAAAGATCGTGTCACAACAAATCAAGAACTTCCCATTATGATTTGA
AGTGATACATTACCGATTGATTCCGTTACGCGGATGACACAAAATATGTCAGTTTAGCACCGGCCGCGCTCCCTTTCCCGGTTTGTTCGGCGACGCGGGGGGAAGGGAGACCGAGCGCACATTAG
CAGGGAAGGTGACAGCGGAGGCTTCACCCATCTCTCTGTCTCTCCGGATCCAGATGTTGGCTTTAAGGTTTCTCCAAACCTAGAAATCTGTTGAAGATATTACTTCTTTGCGAATCTCTCTTTGT
TCTTGAATCGGGTCCAGCGCGGAAGTAGCTGTCGTCAGTAGTAGTTGTGATTCGTGGGAGTTTGAGTTTGATTGGAAGTCCAGTAGTAGTGAGATTCTGCGCCGATCGAGATG

MapGlcT4-1 (XP_009401350.1) (Ma05_p26070.1)

TCGCCTTCGTGTGGGGTTGAAGGGGATTTGGATGGGATGAGGAAGAAGGAAAGAGGAGGAAAAGAAGGCGGTGGTCGGAGGAGGAGAGAGAGTGGCCGACGACATGTGGCTGCGTGTGGCCGCTG
CGCCTCCGCAGTTTTCCTTTTCGGTTGTCGAACCGCAATGACAACAGAAATGACAGCAAATTATATAATAATATTATTATTTTATTTATTTTTAATATTAATAATAAAACAATAATAATAATAAT
ATTATTATTATTATCGAATTCACTTCCCAAACATATCCGATGTTATTGTATTAAATAATTAAAAATATTTTTAAAGATATTTTTAATGTGCATTCGGATAATGTGCATATTTATATCTTAAATAT
GAAGTGATGTAAATACTATTGGTTAAAATATATTTAATTTTTTAATACATATTATATAATAAAATTTATTATTATAATATTTTCAAATATTTATTTTTAGAAAATATATATATATTTTTAAAATTAA
TAAGTAAATAAAAATAAACATACTCTTAAAAAAAAATTCATATTTAACTGATAAAATTATTTGATTATAAATAGCATTTCCATGGGATAGAATCTTATCTTTATCAATTATTTTAAAAAGAAATA
ATAATAATAATAATTAATAAATAATATTGAGGAAACTATCTGTTTATTCTTGAATCTAACTACTTAAGTATTTGAATTAGATTAATCTAATTTCAATCCGATTTTATCTCCACCGAAATAGTGTA
TTGTTGAAAAAAGATTAAGGAAAATTATAATAAAACATACTTAAATTTTATAGTTAATGTTGATTGACTAGTCATTTAATTGTTATTTGACACTCTAATTGAGGTTTATATCTTTCCAAAATTTT
ATTATGATATAAAAGTACATGCATTTAACTCAAAAAAATTAATATTAGTGGTTAATATCTTTTTATCACCCAAACTAACTAATATTCTAAGAAAAAATTATCAAATGAAATATAAATTTTTTTAC
TTTTACTATAGAAATGATTAATTTATGATAACATCAATATTTACAGGGGAATATAATATTCACATGAAATTTATAAGGATAAATACACAATTTTGAAATAGATGGAAATATACGTTAAAACCTCT
AAATCATTAATAGAAACATTAATATATAACATATATTGGAGATTAATCGATCTGTCACAGATATAGTAATACCAAAATCTCGTGCGACCGTCCTTATCATGTGACACGTATGCAGTATTGGACCT
TTTCCCCTGCACTGTGGAATCCAAACCCGCCACTTGCGCTCTCTCTCTCTCTCTGTCTCTCTTCCGTCGATGAGAGCGGATCGTTCCAAGCTTCTGCTACTCTGTTTTGGAGTCTCTCTCTCTCT
CTCTCTCTCTCTCGCTCCTGTCGACGCCCACAAGGGGATTCTTTGCGCTTCGGACTAGCTTGATCGGTAACGGCTCTCAAAGATCGATCTTTTCCTCTCTTTCCTGACCCATTTGAATCGATTGT
TTCGATTATTGAGTGCCAATCTTTTGCTGTAAGGCAAACGTCGTCTTTTTGGGTGATTTCTCTATGTTGCTGTTGTTTTATTGGTCTGTCGAGATCAGAATTGTCCCCATCAATGCAGGCGCTTT
CAATCGTTCCTATTTTTTTTTACTATGTCTATTTATGTGACAGGAGGGGATTAGAAGGGAAAGATG

MapGlcT4-2 (XP_009380955.1) (Ma10_p25440. 1)

GGATACAGGAGTCACCATACTAGACAGCAGGTTATCAACGGATGGCATCGAGAACTCCGGCTGATGAGATAAGCAGTTCTGTTGAGGATAGCACATCAACTCCAAGCTTGAATTCTCCATGGCAG
GGCCTTCTAGCTCTGGAAACTTGCGGGTGAGCTCCAAGCCGGAGTCCTCAAGAAACTCTTCCATGACTACAGGAAAGAGAAAACAAGCGCCTAAAAGAAAGTGTTTGGATGAGAAAGGAGGCCTT
TAGAGTAGGCCTGAACAAGTTCTTCCACCACTATATGAATGCGATGTGTAGAGCAGCTCTCGAGCAGATCAACTTGGCAAATCGAGTTTGTGTAGTCGGCTTGTGGGTGCTCACGGAACACATCT
ACAGCTCTTGGACATATATGTGCTCCAAAATCTGACCATTAGATTCAAGCAGTAAATGAGCATTAATTTGTTCTTTTTGCTCTTGAAGGAAAGTAGAAAACAGATTGCTTTTACAGTATTTTGGT
TTGTGTCAGCTCTTCCTTGAGACTCATACTCTGCTTCAGTGTTCGTCTCCCAATCCAAAGAAAATACTGCAGAAGGGCATCTAACATAGTTCGTACAAGTTGACGATGCTGTGTAGATGGTTAGA
ACTCGTCAATCACAGGGCAGGAGGTTCACCTGTTTTAATCAGTGATCACTAAAGAGAGCCAATGAAGACAAAACCAAACCCCAAAACGAGATCTATGTAAACTTGTTAGAAAGATCGAGTATCTA
CAACGAGGACACTGATCGATGAAGATATCGCCGATTGTTACTGAAAGAACTTAGCTTGTTTTGGTGTATGAGGTAAAGCGGTTCGAGTCTCTTTTCATGCACACCTCTTGGATCTTCCTGTTGTA
CGCGGTTAGATTCGACTAGGTCGTCGCACGCGCAGCACACTTGCCTGAGACGGCGATCTGATTTCTTGACCTTTCGATTCATGACCAAAAGCAAGTTTGTCATGGAATTTAGCATGATCTTAGGG
GTGTGTGTTCCGATGACTATAACAAATCTATACACCATTTCATCTTCTGCGATCCACGCATCATAGATCTTCTACCGACATGAGCAAACAATGCAAAGGAGCGTTCGTTCATCTCGGATCCTCCC
GCCGTACGCCGCCGCAGCGGTTGGAAGGCGCGTACGGCCGTACACGTACACAGAATCCAACACCTCCCACCGACCTAAATCCAGACTCCCTCTTCCTCCTCCTTTCTCTCACTGATCTTTCCACC
ACAGGACGGACGCCCGACATGGCGTCATTCTTGCAACTCCCCGCTGCCTTCCCTACTTCTCGGTAACAAAATAATCTCCCTGTCAAGATTGCTTCTTCCGTCAAAATGGCGGCGATCGCGAAGCT
TCTGTTGCTTCATTCTCTTTCTCTCGCTTCTCGATCTCAGCAAACGGAGGTGTGGAGGATACGTCAAAAGTATTGTCTTTCCACTTCGGATTAGATCGATCGGTGGAAAATTCTGCAAGATCGCC

CCCTTTTTCTATCTTTGCAGCATTTACATCCGTCTTCTTGCCAAATTTCTCTAAAAGTCAACTTTCCATTAACAAGAATAATAATAATAAATGGAATGGCCTGTCTTCGTTGCTATTTCGTTCCT
CGAGATCCGAATTGTCCCTGTCACCAAGGCGGTTTCGATCTGGTTTCTTTCTTCTGTATTTATGACAGGAGGGGATTCGTAGCGAAAGATGGTGCACAACGGTGTGGTCGCTGCCTTTGAGTTTC
CAGGTCGCGGGACAGGGTTTAGGCTGCGCTTGGCTTGGTCAAGAAGAGGAGCGCAAGATCGAACGGGTTGTCGATG
